# Supplementary material for: Bioorthogonal photocatalytic proximity labeling in primary living samples
Source: Nat Commun. 2024 Mar 28;15:2712. doi: 10.1038/s41467-024-46985-3 (PMC10978841; doi:10.1038/s41467-024-46985-3)
Supplement: Supplementary file 1 — Supplementary Information [file 41467_2024_46985_MOESM1_ESM.pdf]

Supplementary Information for

Bioorthogonal Photocatalytic Proximity Labeling in Primary Living Samples

Ziqi Liu[1,2,#], Fuhu Guo[1,#], Yufan Zhu[1], Shengnan Qin[1], Yuchen Hou[1], Haotian Guo[1], Feng Lin[2], Peng R Chen[1,2,*], Xinyuan Fan[1,2,*]

[1]Synthetic and Functional Biomolecules Center, Key Laboratory of Bioorganic Chemistry and Molecular Engineering of Ministry of Education, Beijing National Laboratory for Molecular Sciences, College of Chemistry and Molecular Engineering, Peking University, Beijing 100871, China.

[2]Peking-Tsinghua Center for Life Sciences, Peking University, Beijing 100871, China.

[#]These authors contributed equally.

*E-mail: xinyuanfan@pku.edu.cn (X.F.), pengchen@pku.edu.cn (P.R.C.)

Table of Contents

Chemical Synthesis

Supplementary Tables

Supplementary Figures

NMR Spectra

BSA MS2 Spectra

References

CHEMICAL SYNTHESIS

General consideration

All chemical reagents are in analytical grade, obtained from commercial suppliers, and used without further purification. **AzPh** probe was prepared as previously reported[1]. Reactions were monitored by thin layer chromatography (TLC) carried out on 0.25 mm silica plates (Wish Chemical, Yantai, China), using UV light as the visualizing agent and an ethanolic solution of ammonium molybdate and heat, or ninhydrin and heat as developing agents. Preparative TLC was carried out on 0.5 mm silica plates (Wish Chemical, Yantai, China). If not specially mentioned, flash column chromatography uses silica gel (200-300 mesh) supplied by Tsingtao Haiyang Chemicals (China).

Chromatographic analysis was performed using 1260 infinity II analytical HPLC system (Agilent) equipped with a Poroshell 120 EC-C18 column (2.7 μm, 3.0×150 mm). UPLC-MS analysis was performed on an ACQUITY UPLC I-Class SQD 2(Waters) system equipped with electrospray ionization (ESI) and a BEH C18 Acquity column (1.7 μm, 2.1×50 mm). High resolution mass spectra (HRMS) were recorded on a Fourier Transform Ion Cyclotron Resonance Mass Spectrometer (Solarix XR, Bruker). NMR spectra were recorded on Bruker-500 MHz NMR (AVANCE III), Bruker-400 MHz NMR (AVANCE III) or Bruker-600 MHz (AVANCE Neo) spectrometers and evaluated using MestReNova (Mestrelab Research) software. TMS was used as internal standard for ^{1}H NMR (0.00 ppm), and solvent signal was used as reference for ^{1}H NMR ($CDCl_3$, 7.26 ppm), ^{13}C NMR ($CDCl_3$, 77.16 ppm). The following abbreviations were used to explain the multiplicities: s = singlet, d = doublet, t = triplet, q = quartet, m = multiplet, br = broad.

Synthesis of PAB-caged QM/thioQM probes

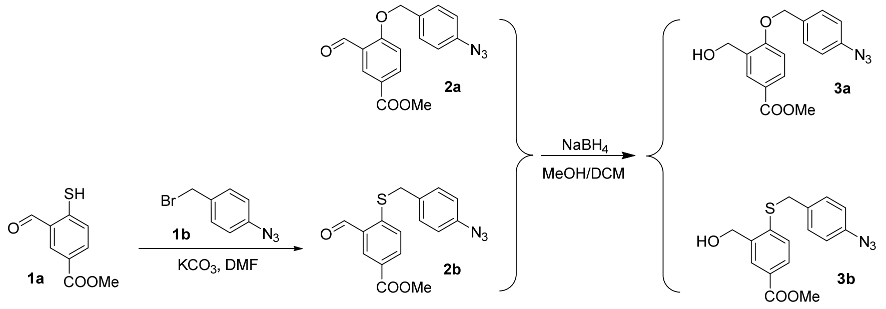

Compounds **1b**[2], **1a**[3], (**2a-b**)[2] and (**3a-b**)[4] were all prepared based on the reported procedures.

*Synthesis of **3a-b***. To a solution of **2a-b** (3.0 mmol) in 2 mL of MeOH and 20 mL of DCM at 0 °C was added $NaBH_4$ (114 mg, 3.0 mmol). The resulting mixture was then stirred at 0 °C for 15 minutes then 30 additional minutes at room temperature under argon. After the reaction was completed, the reaction was stopped by adding 30 mL of a saturated solution of $NaHCO_3$ and the phases were separated. The aqueous phase was extracted twice with 30 mL of CH_2Cl_2, the organic layers were combined, dry over $MgSO_4$ and evaporated to dryness. The crude mixture was purified by Flash Chromatography on SiO_2 gel (elution with PE/EtOAc = 1/1) to afford the **3a-b** as a white solid.

Methyl 3-formyl-4-mercaptobenzoate (**1a**):

^{1}H NMR (400 MHz, Chloroform-d) δ 10.07 (s, 1H), 8.42 – 8.34 (m, 1H), 8.01 (ddd, J = 8.2, 2.0, 1.0 Hz, 1H), 7.38 (d, J = 8.2 Hz, 1H), 5.96 (d, J = 1.2 Hz, 1H), 3.95 (s, 3H); ^{13}C NMR (126

MHz, $CDCl_3$) δ 192.36, 165.59, 144.49, 137.65, 133.45, 131.03, 130.60, 126.99, 52.42.

Methyl 4-((4-azidobenzyl)thio)-3-formylbenzoate (**2b**):

^{1}H NMR (400 MHz, Chloroform-d) δ 10.20 (s, 1H), 8.44 (s, 1H), 8.10 (d, *J* = 8.3 Hz, 1H), 7.44 (d, *J* = 8.3 Hz, 1H), 7.36 (d, *J* = 8.0 Hz, 2H), 6.98 (d, *J* = 8.4 Hz, 2H), 4.21 (s, 2H), 3.94 (s, 3H); ^{13}C NMR (151 MHz, $CDCl_3$) δ 190.67, 165.73, 147.57, 139.65, 134.71, 134.03, 133.00, 131.85, 130.41, 126.96, 126.66, 119.42, 52.39, 36.76.

Methyl 4-((4-azidobenzyl)oxy)-3-(hydroxymethyl)benzoate (**3a**):

^{1}H NMR (400 MHz, Chloroform-*d*) δ 8.00 – 7.86 (m, 2H), 7.33 (d, *J* = 8.5 Hz, 2H), 6.99 (d, *J* = 8.5 Hz, 2H), 6.87 (d, *J* = 8.6 Hz, 1H), 5.06 (s, 2H), 4.67 (s, 2H), 3.81 (s, 3H), 2.12 (s, 1H); ^{13}C NMR (151 MHz, $CDCl_3$) δ 165.69, 158.84, 139.22, 131.65, 130.09, 129.12, 128.52, 127.95, 121.96, 118.38, 110.01, 68.72, 60.34, 50.92.

Methyl 4-((4-azidobenzyl)thio)-3-(hydroxymethyl)benzoate (**3b**):

^{1}H NMR (400 MHz, Chloroform-d) δ 8.05 (d, *J* = 2.0 Hz, 1H), 7.88 (dd, *J* = 8.2, 2.0 Hz, 1H), 7.33 (d, *J* = 8.2 Hz, 1H), 7.28 (d, *J* = 8.5 Hz, 2H), 6.95 (d, *J* = 8.5 Hz, 2H), 4.80 – 4.61 (m, 2H), 4.16 (s, 2H), 3.90 (s, 3H), 2.08 (s, 1H); ^{13}C NMR (151 MHz, $CDCl_3$) δ 166.72, 141.07, 139.81, 139.46, 132.95, 130.24, 129.13, 128.81, 128.01, 127.85, 119.30, 62.98, 52.15, 37.52.

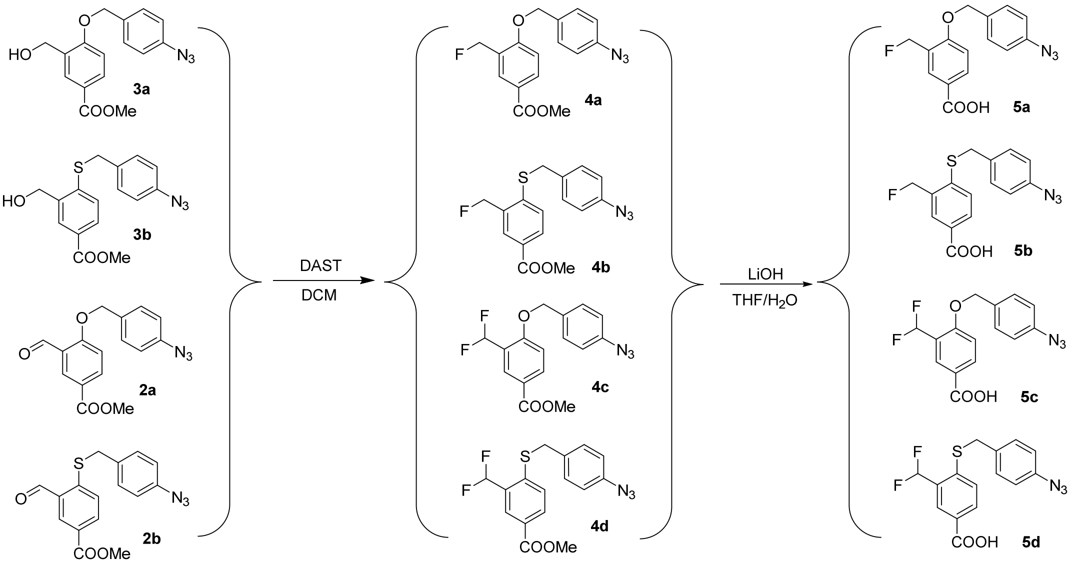

Compounds (**4a-b**)[5], (**4c-d**)[2], (**5a-b,5d**)[5] , **5c**[2] and were all prepared based on the reported procedures.

*Synthesis of **4a-b***. Diethylaminosulfur trifluoride (590 µL, 4.4 mmol) was added to a solution of 3a-b (2.0 mmol) in DCM (15 mL) at 0ºC. The reaction mixture was stirred at r.t. overnight, washed with H_2O (20 mL ×3) and brine (20 mL ×1). The crude product was purified by column chromatography (SiO_2, Hexane/EtOAc = 2/1) to afford the 4a-b.

*Synthesis of **5a-b,5d***. 1 M LiOH aqueous solution (600 µL, 0.6 mmol) was added to a solution of (4a-b, 4d) (0.2 mmol) in THF/H_2O (2.4 mL/0.8 mL) at 0oC. The reaction mixture was stirred at r.t. overnight, diluted with 4 mL of H_2O, neutralized with 1M HCl on ice, extracted with EtOAc, and dried over Na_2SO_4. The crude product was used in next step without further purification.

Methyl 4-((4-azidobenzyl)oxy)-3-(fluoromethyl)benzoate **4a:**

^{1}H NMR (400 MHz, Chloroform-*d*) δ 8.06 – 7.91 (m, 2H), 7.33 (d, *J* = 8.5 Hz, 2H), 6.98 (d, *J* = 8.5 Hz, 2H), 6.88 (d, *J* = 9.9 Hz, 1H), 5.47 (s, 1H), 5.35 (s, 1H), 5.06 (s, 2H), 3.82 (s, 3H); ^{13}C NMR (151 MHz, $CDCl_3$) δ 165.48, 158.66, 158.64, 139.11, 131.66, 131.18, 131.16, 129.81, 129.76, 127.82, 124.26, 124.15, 121.92, 118.31, 110.20, 79.49, 78.39, 68.71, 50.97.

Methyl 4-((4-azido-2-nitrobenzyl)thio)-3-(fluoromethyl)benzoate **4b:**

^{1}H NMR (400 MHz, Chloroform-*d*) δ 8.05 (s, 1H), 7.93 (dt, *J* = 8.2, 1.6 Hz, 1H), 7.36 (d, *J* = 8.3 Hz, 1H), 7.28 (s, 1H), 7.26 (s, 1H), 6.95 (d, *J* = 8.5 Hz, 2H), 5.49 (d, *J* = 3.1 Hz, 1H), 5.37 (d, *J* = 3.1 Hz, 1H), 4.15 (s, 2H), 3.91 (s, 3H); ^{13}C NMR (151 MHz, $CDCl_3$) δ 166.41, 141.28, 141.25, 139.47, 135.83, 135.72, 132.86, 130.04, 130.02, 129.23, 129.18, 128.71, 128.09, 119.29, 82.54, 81.42, 52.22, 37.83.

Methyl 4-((4-azidobenzyl)thio)-3-(difluoromethyl)benzoate **4d:**

^{1}H NMR (400 MHz, Chloroform-d) δ 8.29 – 8.24 (m, 1H), 8.01 (ddt, *J* = 8.2, 1.9, 1.0 Hz, 1H), 7.41 (dd, *J* = 8.3, 1.3 Hz, 1H), 7.26 – 7.18 (m, 2H), 6.98 – 6.90 (m, 3H), 4.13 (s, 2H), 3.93 (s, 3H); ^{13}C NMR (126 MHz, $CDCl_3$) δ 165.93, 140.87, 140.82, 140.78, 139.57, 134.48, 134.30, 134.13, 132.66, 131.63, 131.62, 131.60, 130.72, 130.24, 128.82, 127.24, 127.18, 127.13, 119.31, 113.99, 112.09, 110.19, 52.39, 38.77; ^{19}F NMR (471 MHz, $CDCl_3$) δ -113.40.

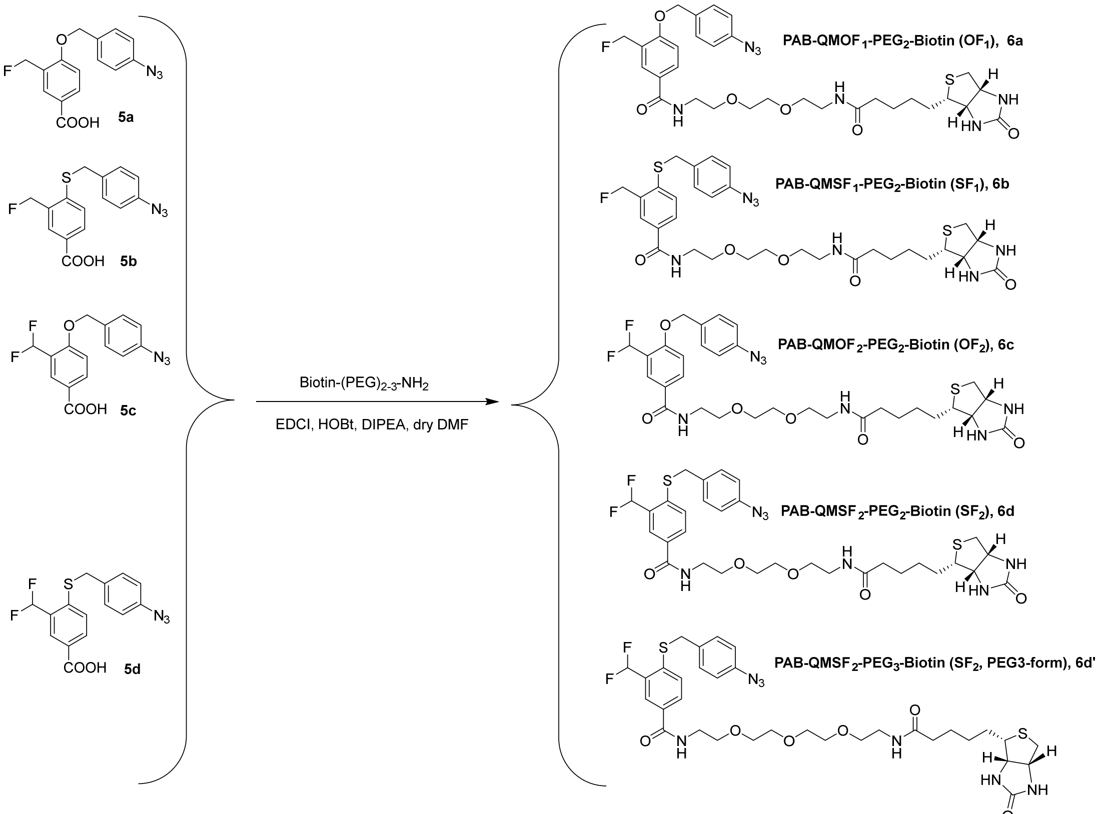

Compounds **6a-d'** were prepared based on the reported conjugation procedure[2].

PAB-QMOF$_1$-PEG2-Bio (**OF1**), **6a:**

^{1}H NMR (400 MHz, Chloroform-*d*) δ 7.85 (d, *J* = 10.9 Hz, 2H), 7.39 (d, *J* = 8.5 Hz, 2H), 7.19 (s, 1H), 7.04 (d, *J* = 8.4 Hz, 2H), 6.95 (d, *J* = 8.4 Hz, 1H), 6.64 (s, 1H), 6.33 (s, 1H), 5.53 (s, 1H), 5.41 (s, 1H), 5.10 (s, 2H), 4.42 (s, 1H), 4.23 (s, 1H), 3.71 – 3.31 (m, 12H), 3.12 – 3.02 (m, 1H), 2.84 (dd, *J* = 12.9, 4.8 Hz, 1H), 2.66 (d, *J* = 12.8 Hz, 1H), 2.14 (t, *J* = 7.5 Hz, 2H), 1.59 (dq, *J* = 14.6,

7.3, 6.9 Hz, 4H), 1.35 (t, *J* = 7.8 Hz, 2H); ^{13}C NMR (151 MHz, $CDCl_3$) δ 173.65, 167.05, 164.03, 158.52, 158.49, 140.06, 132.87, 129.70, 129.68, 128.89, 128.05, 128.00, 126.91, 125.19, 125.08, 119.31, 111.52, 80.70, 79.60, 70.08, 70.04, 70.01, 69.71, 61.80, 60.25, 55.37, 40.45, 39.84, 39.16, 39.14, 35.77, 27.99, 27.96, 25.42; **FT-HRMS (ESI)** m/z calcd. for $C_{31}H_{41}FN_7O_6S$ $[M+H]^+$: 658.2819; Found: 658.2822.

PAB-QMSF$_1$-PEG$_2$-Bio (**SF1**), **6b**

^{1}H NMR (400 MHz, Chloroform-*d*) δ 7.86 (s, 1H), 7.78 (d, *J* = 8.2 Hz, 1H), 7.38 (d, *J* = 8.2 Hz, 1H), 7.23 (dd, *J* = 16.0, 8.1 Hz, 3H), 6.93 (dd, *J* = 8.7, 2.8 Hz, 2H), 6.56 (d, *J* = 15.5 Hz, 2H), 5.51 (d, *J* = 16.5 Hz, 2H), 5.37 (s, 1H), 4.51 – 4.41 (m, 1H), 4.30 – 4.20 (m, 1H), 4.13 (s, 2H), 3.71 – 3.50 (m, 10H), 3.41 (dd, *J* = 10.2, 5.2 Hz, 2H), 3.15 – 3.06 (m, 1H), 2.87 (dd, *J* = 12.8, 4.8 Hz, 1H), 2.70 (d, *J* = 12.8 Hz, 1H), 2.18 (t, *J* = 7.4 Hz, 2H), 1.73 – 1.55 (m, 4H), 1.39 (p, *J* = 7.8 Hz, 2H); ^{13}C NMR (126 MHz, $CDCl_3$) δ 173.40, 166.75, 164.02, 139.36, 139.07, 139.04, 136.18, 136.05, 133.12, 132.52, 130.27, 129.63, 127.95, 127.93, 126.82, 126.75, 119.24, 82.89, 81.56, 70.12, 69.98, 69.88, 69.85, 61.74, 60.20, 55.50, 40.46, 39.92, 39.10, 38.14, 35.92, 28.09, 28.01, 25.51; **FT-HRMS (ESI)** m/z calcd. for $C_{31}H_{41}FN_7O_5S_2$ $[M+H]^+$: 674.2589; Found: 674.2593.

PAB-QMOF$_2$-PEG$_2$-Bio (**OF2**), **6c**

^{1}H NMR (400 MHz, Chloroform-*d*) δ 8.06 (d, *J* = 2.3 Hz, 1H), 7.97 (dd, *J* = 8.6, 2.3 Hz, 1H), 7.43 – 7.37 (m, 2H), 7.33 (t, *J* = 5.3 Hz, 1H), 7.04 (dd, *J* = 10.8, 8.4 Hz, 3H), 6.96 (s, 1H), 6.76 (t, *J* = 5.6 Hz, 1H), 6.70 (s, 1H), 5.73 (s, 1H), 5.14 (s, 2H), 4.45 (dd, *J* = 7.8, 4.8 Hz, 1H), 4.29 – 4.21 (m, 1H), 3.71 – 3.59 (m, 8H), 3.55 (t, *J* = 5.1 Hz, 2H), 3.41 (td, *J* = 5.2, 2.7 Hz, 2H), 3.09 (td, *J* = 7.3, 4.5 Hz, 1H), 2.85 (dd, *J* = 12.8, 4.8 Hz, 1H), 2.69 (d, *J* = 12.8 Hz, 1H), 2.18 (t, *J* = 7.5 Hz, 2H), 1.64 (qq, *J* = 13.3, 7.4, 6.7 Hz, 4H), 1.39 (p, *J* = 7.3 Hz, 2H); ^{13}C NMR (126 MHz, $CDCl_3$) δ 173.48, 166.38, 164.22, 158.58, 158.54, 158.50, 140.19, 132.40, 131.67, 128.96, 127.17, 125.60, 125.55, 125.50, 122.98, 122.80, 122.62, 119.34, 113.19, 112.13, 111.31, 109.42, 70.12, 70.01, 69.98, 69.88, 61.75, 60.20, 55.62, 40.45, 39.92, 39.10, 35.93, 28.16, 28.02, 25.57; ^{19}F NMR (471 MHz, $CDCl_3$) δ -115.61; **FT-HRMS (ESI)** m/z calcd. for $C_{31}H_{40}F_2N_7O_6S$ $[M+H]^+$: 676.2723; Found: 676.2732.

PAB-QMSF$_2$-PEG$_2$-Bio (**SF2**), **6d**

^{1}H NMR (400 MHz, Chloroform-d) δ 8.06 (s, 1H), 7.89 (d, *J* = 8.2 Hz, 1H), 7.44 (d, *J* = 8.1 Hz, 2H), 7.21 (d, *J* = 8.0 Hz, 2H), 6.93 (d, *J* = 7.8 Hz, 2H), 6.64 (d, *J* = 6.1 Hz, 2H), 5.68 (s, 1H), 4.46 (s, 1H), 4.26 (s, 1H), 4.11 (s, 2H), 3.73 – 3.49 (m, 10H), 3.45 – 3.33 (m, 2H), 3.10 (d, *J* = 5.9 Hz, 1H), 2.86 (dd, *J* = 12.9, 4.8 Hz, 1H), 2.70 (d, *J* = 12.8 Hz, 1H), 2.18 (t, *J* = 7.5 Hz, 2H), 1.64 (s, 4H), 1.39 (t, *J* = 7.7 Hz, 2H); ^{13}C NMR (151 MHz, $CDCl_3$) δ 173.50, 166.28, 164.16, 139.47, 138.75, 138.72, 138.68, 134.58, 134.43, 134.28, 133.27, 132.93, 131.57, 130.29, 129.79, 128.97, 124.67, 124.63, 124.59, 119.29, 113.94, 112.36, 110.78, 70.16, 70.01, 69.89, 69.78, 61.78, 60.23, 55.57, 40.46, 40.01, 39.11, 39.08, 35.92, 28.14, 28.03, 25.54; ^{19}F NMR (565 MHz, $CDCl_3$) δ -112.78, -112.88; **FT-HRMS (ESI)** m/z calcd. for $C_{31}H_{40}F_2N_7O_5S_2$ $[M+H]^+$: 692.2504; Found: 692.2495.

PAB-QMSF$_2$-PEG$_3$-Bio (**SF2,** PEG3-form), **6d’**

^{1}H NMR (400 MHz, Chloroform-d) δ 8.05 (d, *J* = 2.0 Hz, 1H), 7.90 (dd, *J* = 8.2, 2.0 Hz, 1H), 7.43 (t, *J* = 7.5 Hz, 2H), 7.24 – 7.18 (m, 2H), 6.96 – 6.89 (m, 2H), 6.60 (t, *J* = 5.6 Hz, 1H), 6.35 (s, 1H), 5.38 (s, 1H), 4.48 (dd, *J* = 7.9, 4.8 Hz, 1H), 4.27 (dd, *J* = 8.2, 4.6 Hz, 1H), 4.11 (s, 2H), 3.72 – 3.56 (m, 12H), 3.52 (t, *J* = 5.1 Hz, 2H), 3.39 (h, *J* = 4.0 Hz, 2H), 3.11 (td, *J* = 7.3, 4.4 Hz, 1H), 2.88 (dd, *J* = 12.8, 4.9 Hz, 1H), 2.72 (d, *J* = 12.8 Hz, 1H), 2.19 (t, *J* = 7.4 Hz, 2H), 1.67 (dtq, *J* = 19.9, 13.5, 7.3, 6.9 Hz, 4H), 1.40 (p, *J* = 7.6 Hz, 2H); ^{13}C NMR (126 MHz, $CDCl_3$) δ 173.41, 166.25,

163.87, 139.44, 138.59, 134.42, 133.32, 132.91, 131.64, 130.27, 129.89, 124.56, 119.27, 114.24, 112.34, 110.44, 70.33, 70.26, 70.14, 69.98, 69.95, 69.86, 61.77, 60.19, 55.47, 40.48, 39.97, 39.10, 35.83, 28.07, 28.03, 25.50; ^{19}F NMR (471 MHz, $CDCl_3$) δ-112.82; **FT-HRMS (ESI)** m/z calcd. for $C_{33}H_{44}F_2N_7O_6S_2$ $[M+H]^+$: 736.2758; Found: 736.2751.

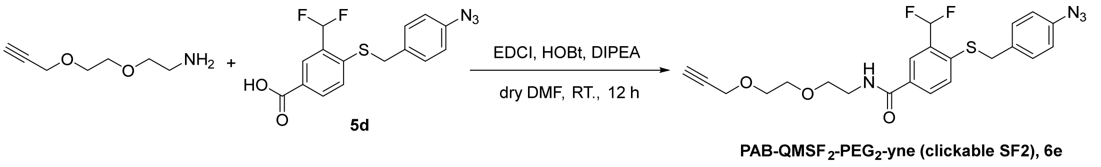

Compounds **6e** was prepared based on the reported conjugation procedure[2].

PAB-QMSF2-PEG2-yne (clickable **SF2**), **6e**:

^{1}H NMR (400 MHz, Chloroform-*d*) δ 8.03 – 7.98 (m, 1H), 7.87 – 7.80 (m, 1H), 7.41 (d, *J* = 8.0 Hz, 1H), 7.22 – 7.16 (m, 2H), 6.94 (d, *J* = 1.1 Hz, 2H), 6.92 (s, 1H), 4.18 (d, *J* = 2.4 Hz, 2H), 4.09 (s, 2H), 3.75 – 3.63 (m, 8H), 2.42 (t, *J* = 2.4 Hz, 1H); ^{13}C NMR (151 MHz, $CDCl_3$) δ 166.04, 139.44, 138.45, 138.42, 138.38, 134.93, 134.78, 134.63, 133.52, 132.98, 131.99, 130.23, 129.68, 124.46, 124.42, 124.37, 119.26, 113.85, 112.27, 110.69, 79.37, 74.86, 69.99, 69.64, 69.01, 58.39, 39.82, 39.25; ^{19}F NMR (565 MHz, $CDCl_3$) δ -112.91, -113.01.

Synthesis of ONP-caged QM probe

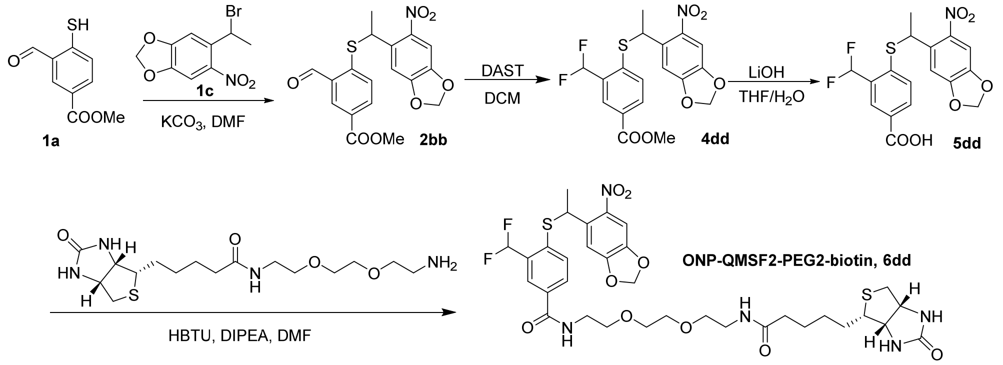

Compounds **1c**[6], **2bb**[2], **4dd**[5], **5dd**[2] and **6dd**[5], were prepared based on reported procedures.

*Synthesis of **5dd**.* 1 M LiOH aqueous solution (600 µL, 0.6 mmol) was added to a solution of (4aa-dd, 2e-f) (0.2 mmol) in THF/H_2O (2.4 mL/0.8 mL) at 0°C. The reaction mixture was stirred at r.t. overnight, diluted with 4 mL of H_2O, neutralized with 1M HCl on ice, extracted with EtOAc, and dried over Na_2SO_4. The crude product was used in next step without further purification.

Methyl 3-formyl-4-((1-(6-nitrobenzo[*d*][1,3]dioxol-5-yl)ethyl)thio)benzoate **2bb:**

^{1}H NMR (400 MHz, Chloroform-*d*) δ 10.24 (s, 1H), 8.42 (d, *J* = 2.0 Hz, 1H), 8.01 (dd, *J* = 8.3, 2.0 Hz, 1H), 7.38 (s, 1H), 7.28 (d, *J* = 13.8 Hz, 2H), 6.08 (dd, *J* = 9.1, 1.2 Hz, 2H), 5.46 (q, *J* = 6.9 Hz, 1H), 3.92 (s, 3H), 1.72 (d, *J* = 7.0 Hz, 3H); ^{13}C NMR (126 MHz, $CDCl_3$) δ 190.70, 165.61, 152.31, 147.24, 145.62, 142.80, 134.41, 134.29, 134.17, 133.62, 128.21, 127.59, 107.46, 105.33, 103.12, 52.40, 40.58, 22.59.

Methyl 3-(difluoromethyl)-4-((1-(6-nitrobenzo[*d*][1,3]dioxol-5-yl)ethyl)thio)benzoate **4dd:**

^{1}H NMR (400 MHz, Chloroform-*d*) δ 8.23 (s, 1H), 7.96 (d, *J* = 8.2 Hz, 1H), 7.36 (d, *J* = 8.2 Hz, 1H), 7.28 (d, *J* = 2.5 Hz, 1H), 7.21 (s, 1H), 6.09 (s, 2H), 5.36 (q, *J* = 6.9 Hz, 1H), 3.91 (d, *J* =

2.1 Hz, 3H), 1.68 (d, *J* = 1.9 Hz, 3H); ^{13}C NMR (126 MHz, $CDCl_3$) δ 165.75, 152.12, 147.15, 142.85, 139.42, 139.37, 139.33, 134.91, 134.73, 134.56, 134.04, 131.69, 129.33, 127.33, 127.28, 127.23, 113.99, 112.09, 110.19, 107.49, 105.07, 103.14, 52.37, 42.61, 22.42.

ONP-QMSF$_2$-PEG$_2$-Biotin (**SF$_2$^{UV}**), **6dd:**

^{1}H NMR (400 MHz, Chloroform-*d*) δ 7.99 (s, 1H), 7.76 (d, *J* = 8.2 Hz, 1H), 7.57 – 7.49 (m, 1H), 7.29 (d, *J* = 8.2 Hz, 1H), 7.11 (s, 1H), 6.73 (t, *J* = 5.7 Hz, 1H), 6.60 (s, 1H), 6.03 (d, *J* = 4.9 Hz, 2H), 5.75 (s, 1H), 5.21 (q, *J* = 6.9 Hz, 1H), 4.45 – 4.34 (m, 1H), 4.24 – 4.15 (m, 1H), 3.57 (dd, *J* = 15.7, 4.3 Hz, 8H), 3.48 (t, *J* = 5.2 Hz, 2H), 3.33 (q, *J* = 5.9 Hz, 2H), 3.04 (td, *J* = 7.3, 4.3 Hz, 1H), 2.80 (dd, *J* = 12.9, 4.8 Hz, 1H), 2.63 (d, *J* = 12.7 Hz, 1H), 2.11 (t, *J* = 7.5 Hz, 2H), 1.57 (t, *J* = 8.1 Hz, 7H), 1.33 (q, *J* = 7.7 Hz, 2H); ^{13}C NMR (126 MHz, $CDCl_3$) δ 173.57, 166.16, 164.25, 152.12, 147.12, 142.81, 137.08, 137.04, 136.99, 135.31, 135.13, 134.95, 134.14, 134.01, 132.73, 132.71, 129.77, 125.04, 124.99, 124.94, 114.26, 112.36, 110.46, 107.52, 105.06, 103.15, 70.14, 69.99, 69.89, 69.73, 61.79, 60.23, 55.64, 42.88, 40.45, 39.99, 39.06, 35.91, 28.20, 28.04, 25.57, 22.38; **FT-HRMS (ESI)** m/z calcd. for $C_{33}H_{42}F_2N_5O_9S_2$ $[M+H]^+$: 754.2387; Found: 754.2388.

SUPPLEMENTARY TABLES

Supplementary Table 1. Antibodies used in this work

Name	Host species	Catalog	Company	Dilution
Anti-ACSM2A mAb	Rabbit	ab181865	Abcam	1:2000
Anti-AIF mAb	Rabbit	ab32516	Abcam	1:500
Anti-Aldh3a2 mAb	Rabbit	ab184171	Abcam	1:2000
Anti-biotin mAb	Mouse	sc-101339	Santa Cruz	1:1000
Anti-CPT1B pAb	Rabbit	ab134988	Abcam	1:1000
Anti-HSP60 mAb	Rabbit	ab45134	Abcam	1:2000
Anti-Mouse-AlexaFluor555	Goat	A-21422	Invitrogen	1:500
Anti-Rabbit-AlexaFluor488	Goat	A-11008	Invitrogen	1:500
Anti-Rabbit-AlexaFluor546	Goat	A-11010	Invitrogen	1:500
Anti-V5 mAb	Mouse	B1005	Biodragon	1:1000
HRP-linked anti-mouse IgG	Horse	7076S	Cell Signaling Technology	1:5000
HRP-linked anti-rabbit IgG	Goat	7074S	Cell Signaling Technology	1:5000
Streptavidin-AlexaFluor488		S11223	Invitrogen	1:500
Streptavidin-HRP		3999S	Cell Signaling Technology	1:1000

Supplementary Table 2. Observed **SF2-**modified peptides for *in vitro* BSA labeling

**Peptide sequence***	**Modification site on BSA**
KVPQVSTPTLVEVSR	Q440
KFWGKYLYEIAR	K156, W158
KVPQVSTPTLVEVSR	K437
CCTESLVNR	T501
GLVLIAFSQYLQQCPFDEHVK	Y54, Q56
KFWGKYLYEIAR	K160, Y161
KFWGKYLYEIAR	W158, K160
LIAFSQYLQQCPFDEHVK	Q57
NECFLSHKDDSPDLPKLKPDPNTLCDEFK	K140
PLLEKSHCIAEVEKDAIPENLPPLTADFAEDK	E308, K309
RHPYFYAPELLYYANK	N182
CVADESHAGCEK	D80
LKPDPNTLCDEFK	K140, D142
VKLVNELTEFAK	K65

*The labeling site(s) in sequence are colored red.

Supplementary Table 3. Primers for molecular cloning of “mito orphans” candidates

Gene name	Sequence (5’ to 3’)*	
MDH2 (mito control)	F	AGAATTCGCCACCATGCTCTCCGCCCTCG
	R	CCGCTGCTGCTACCCTTCAGGGTCTTCACGAA
SLC25A5 (mito control)	F	AGAATTCGCCACCATGACAGATGCCGCTGTG
	R	CCGCTGCTGCTACCTGTGTACTTCTTGATTTCATCATAC
ACTN4 (non-mito control)	F	AGAATTCGCCACCATGGTGGACTACCACGCGG
	R	CCGCTGCTGCTACCCAGGTCGCTCTCGCC
PTPN1	F	AGAATTCGCCACCATGGAGATGGAAAAGGAGTTCG
	R	CCGCTGCTGCTACCTGTGTTGCTGTTGAACAG
SLC35A4 upstream ORF	F	AGAATTCGCCACCATGGCGGATGACAAGGATTCTC
	R	CCGCTGCTGCTACCGTCGGGCCCCTTG
TMEM160	F	AGAATTCGCCACCATGGGAGGCGGCTGGT
	R	CGCTGCTGCTACCCTCGGGTGGCGG
TRABD	F	AGAATTCGCCACCATGGACGGGGAGGAGCAG
	R	CGCTGCTGCTACCCTTGTGCCGGGCCT
DPM3	F	AGAATTCGCCACCATGACGAAATTAGCGCAGTGG
	R	CGCTGCTGCTACCGAAGCGCAGCCCCCT
PRAF2	F	AGAATTCGCCACCATGTCGGAGGTGCGGCT
	R	CCGCTGCTGCTACCGGATCCAGCCTCCTG
ISOC1	F	AGAATTCGCCACCATGGCGGCTGCGGAG
	R	CCGCTGCTGCTACCTACTTTGGAAAGCAGACCC

* Sequences aligning target gene in human cDNA are colored blue.

SUPPLEMENTARY FIGURES

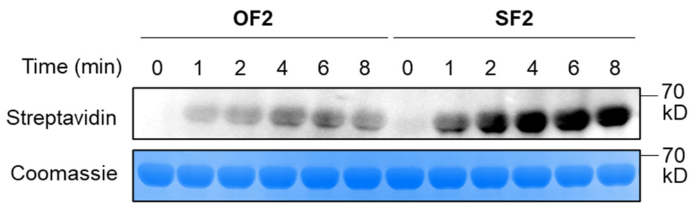

Supplementary Fig. 1. Time-resolved *in vitro* photocatalytic labeling for comparison between OF2 and SF2 probes

BSA (1 mg/mL, in PBS) was treated with **Ir1** (5 µM), QM probe (100 µM) and NADH (500 µM) for labeling (450 nm blue LED, ~4 mW/cm^2). n = 2 biological replicates.

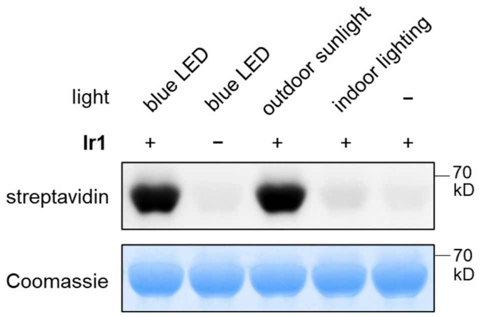

Supplementary Fig. 2. ***In vitro*** **photocatalytic labeling with different light sources**
BSA (1 mg/mL, in PBS) was treated with **Ir1** (5 µM), QM probe (100 µM) and NADH (500 µM) for labeling (5 min). blue LED (450 nm): ~4 mW/cm^2; outdoor sunlight: ~50 mW/cm^2; ambient indoor lighting (ceiling light): 1~2 mW/cm^2 white light. n = 2 biological replicates.

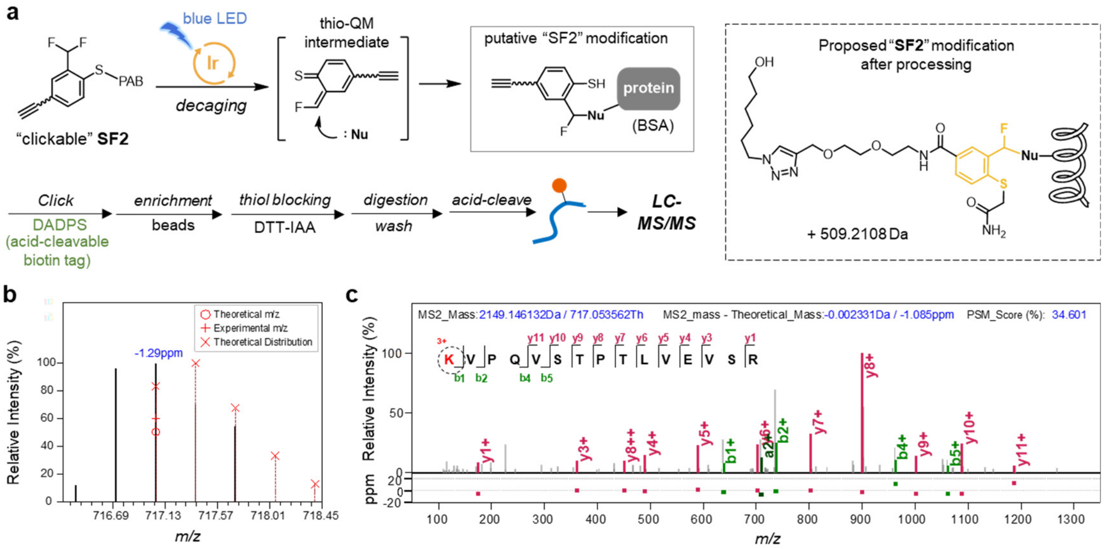

Supplementary Fig. 3. Identification of SF2-mediated modification by LC-MS/MS

a, Workflow of LC-MS/MS identification of **SF2**-mediated modification on BSA. To purify labeled peptides, "clickable" **SF2** probe with an alkynyl handle was used for protein labeling (under the same condition as Fig. 2b) followed by conjugation with acid-cleavable biotin tag, which enabled the release of labeled peptides from streptavidin beads upon acid treatment. The chemical structure of target modification after processing for search is shown in the right. **b-c**, MS1 (**b**) and MS2 (**c**) spectra of representative **SF2**-modified peptide (K437-R451), searched by using pFind (version 3.1.5) software. The modification site (K437) is highlighted in red and circled.

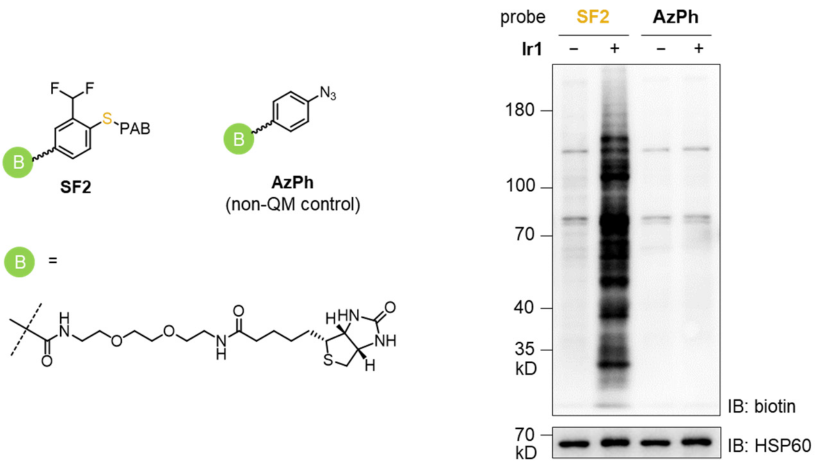

Supplementary Fig. 4. CAT-S photocatalytic labeling in living cells with additional control
HeLa cells were treated with 100 nM **Ir1** and 100 μM probe for labeling. HSP60 was blotted as the loading control. The results indicated that CAT-S labeling was dependent on thioQM moiety instead of arylazide group. n = 2 biological replicates.

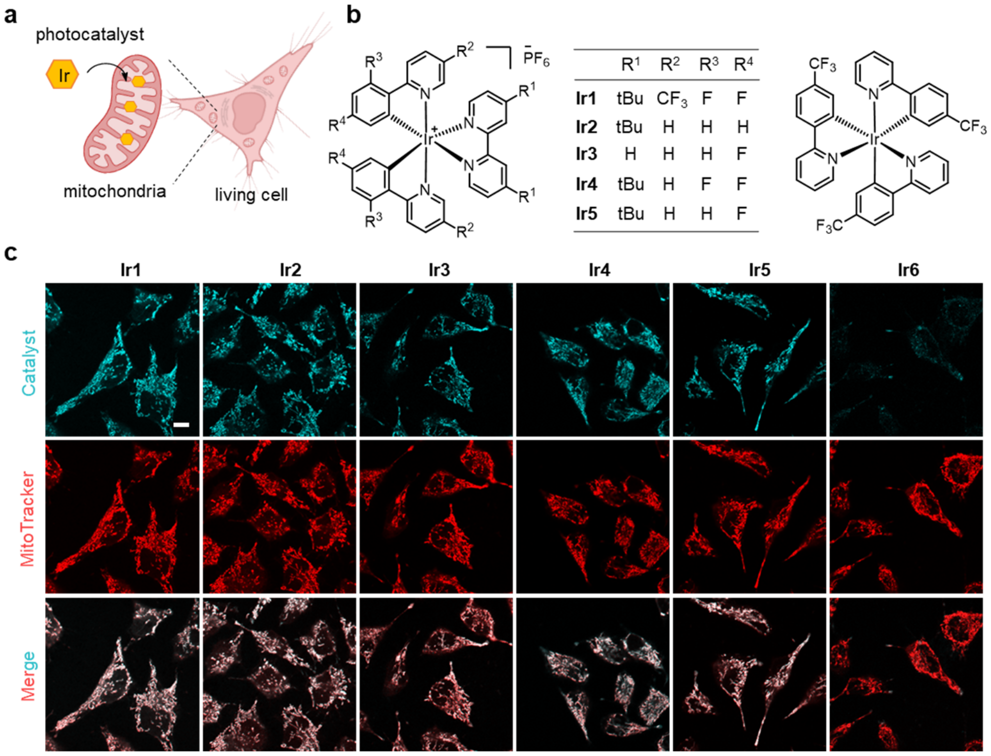

Supplementary Fig. 5. Imaging of iridium photocatalyst in living HeLa cells

a, Schematic view of mitochondrial targeting. **b**, Chemical structures of iridium photocatalyst **Ir1-6**. **c**, HeLa cells were incubated with photocatalyst (0.5 µM) and MitoTracker Deep Red (100 nM) for 0.5 h followed by confocal imaging. Blue, fluorescence of photocatalyst. Red, mitochondria stained by MitoTracker Deep Red. Scale bar, 5 µm. *n* = 2 biological replicates. Created with BioRender.com (Agreement number KQ26IJP61A).

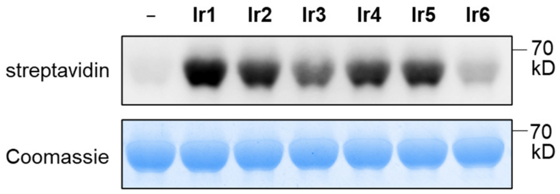

Supplementary Fig. 6. *In vitro* photocatalytic labeling for comparison between different catalyst.

BSA (1 mg/mL, in PBS) was treated with iridium catalyst (5 μM), QM probe (100 μM) and NADH (500 μM) for labeling (450 nm blue LED, ~4 mW/cm^2, 5 min). $n = 2$ biological replicates.

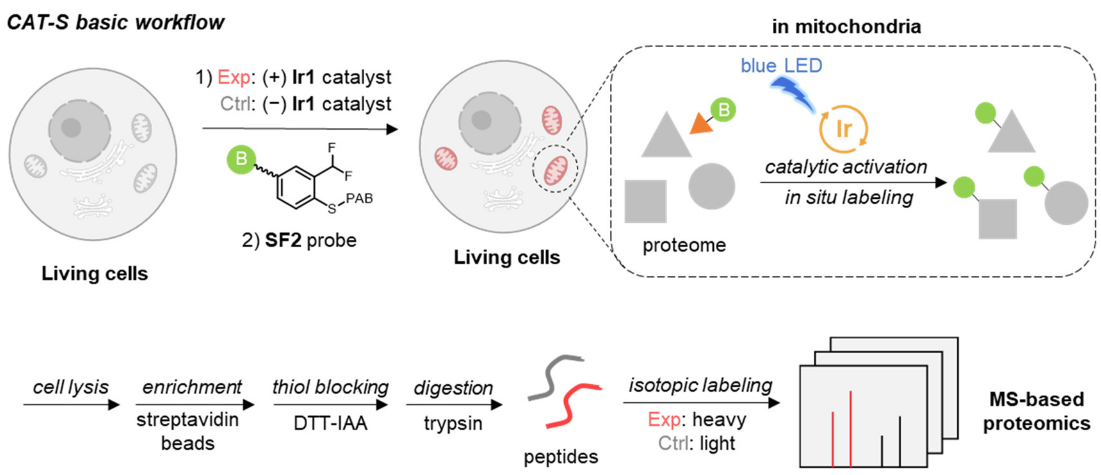

Supplementary Fig. 7. Basic workflow for CAT-S mitochondrial proteomics, related to Fig. 3 Living cells were sequentially incubated with photocatalyst **Ir1** (for control group, **Ir1** was omitted) and **SF2** probe, followed by irradiation by blue LED (~450 nm) to trigger bioorthogonal photocatalysis for labeling in living cells. After labeling, cells were lysed, followed by enrichment of biotinylated proteins, thiol blocking and enzymatic digestion. Digested peptides of experiment group (+ catalyst, + probe) and control group (− catalyst, + probe) were labeled by heavy and light isotopes respectively, and subjected to MS-based proteomic analysis. Created with BioRender.com (Agreement number HO26IJPEWM).

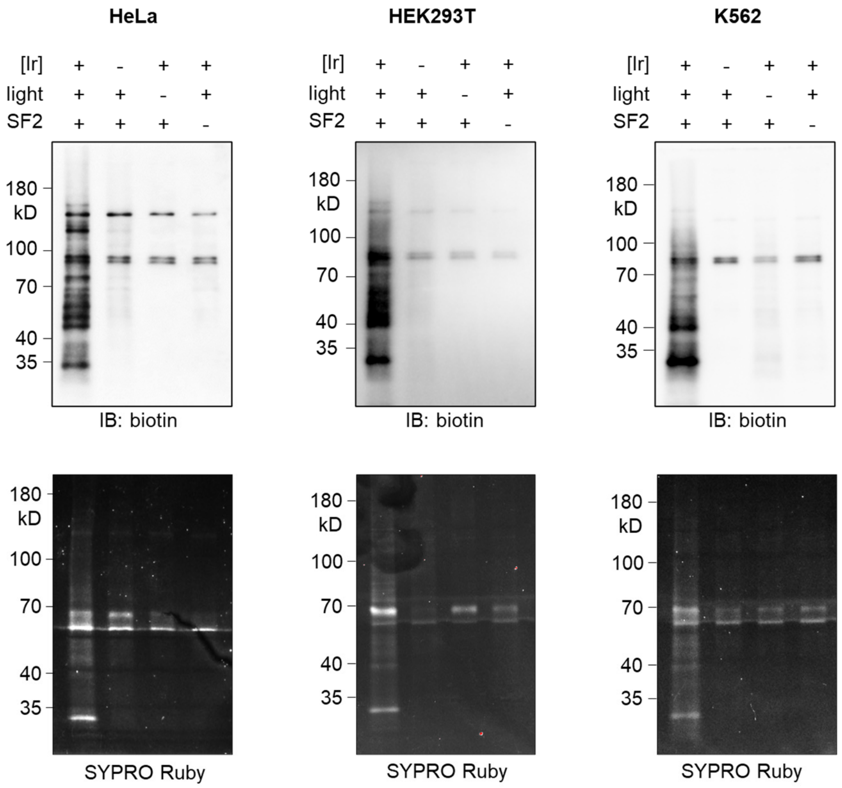

Supplementary Fig. 8. Immunoblotting and SYPRO Ruby staining of pulldown samples from CAT-S labeling, related to Fig. 3

HeLa, HEK293T and K562cells (from left to right) were treated with/without **Ir1** (100 nM) and **SF2** (100 μM) for photocatalytic labeling. Samples after enrichment by streptavidin beads were subjected to immunoblotting and SYPRO Ruby staining (a more facile alternative to silver staining). *n* = 2 biological replicates.

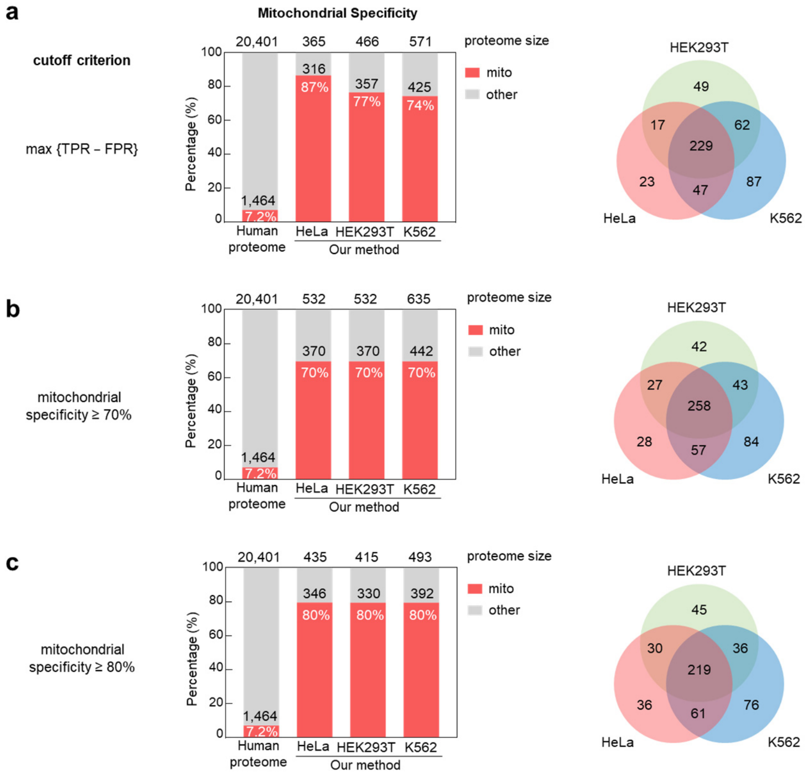

Supplementary Fig. 9. CAT-S mitochondrial proteome sizes of cell lines with additional cutoff criteria

a, Maximization of (true-positive rate – false-positive rate) (i.e., TPR – FPR). **b**, Mitochondrial specificity ⩾ 70%. **c**, Mitochondrial specificity ⩾ 80%. Left column, mitochondrial specificity analysis showing the fraction of proteome with prior mitochondrial annotation in database. Right column, Venn diagram showing the overlap between the captured mitochondrial proteomes.

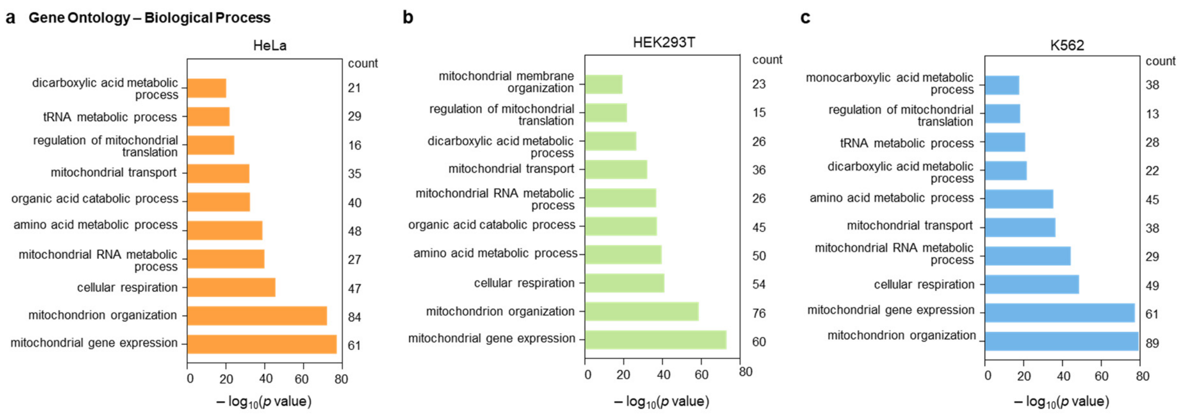

Supplementary Fig. 10. GO analysis of the captured mitochondrial proteomes from different cell lines

Gene Ontology Biological Process analysis of the captured mitochondrial proteome from HeLa (**a**), HEK293T (**b**) and K562 (**c**) cells. Top 10 enriched terms are shown, with *p* values calculated by cumulative hypergeometric test.

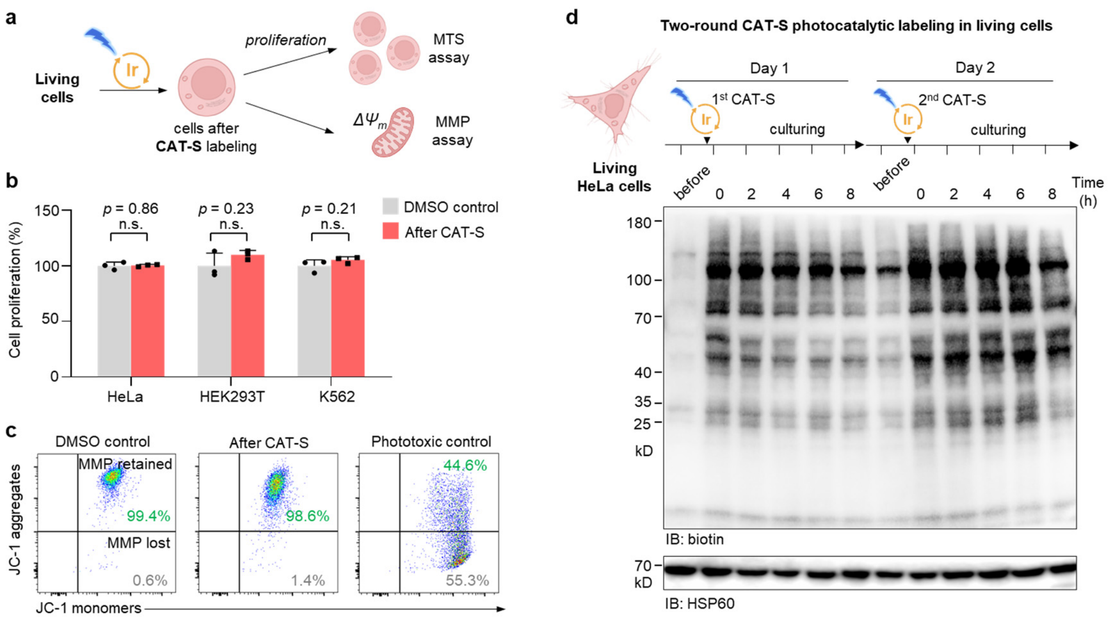

Supplementary Fig. 11. Bioorthogonality of the CAT-S system

a, Schematic view of the proliferation measurement after CAT-S labeling (under the same labeling condition for proteomics study). **b**, Proliferation measurement based on MTS assay. Data are represented as mean ± SD ($n = 3$ samples). n.s., $p > 0.05$, unpaired two-tailed t test. **c**, Mitochondrial membrane potential (MMP) characterization for HeLa cells by JC-1 dyeing and flowcytometry. JC-1 aggregate (generated in the presence of MMP) signal indicates the health of mitochondria. $n = 2$ samples. **d**, Living HeLa cells that underwent CAT-S photocatalytic labeling (under the same condition for proteomics study) twice in two days were tracked by immunoblotting to measure the extent of labeling. $n = 2$ biological replicates. HSP60 was blotted as internal reference. The 2nd CAT-S was performed 20 h after 1st CAT-S. Source data are provided as a Source Data file. Created with BioRender.com (Agreement number AE26IJPSQX).

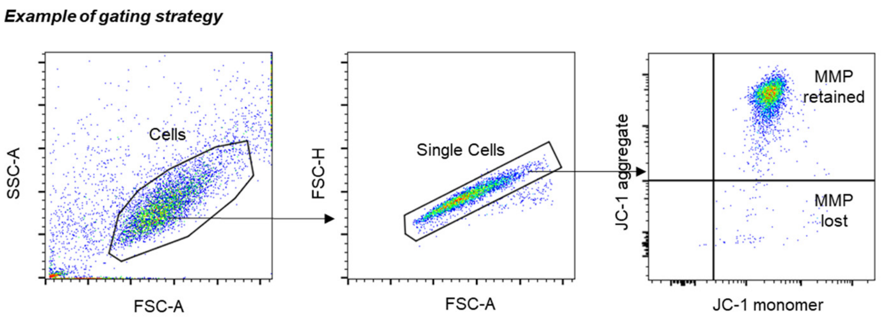

Supplementary Fig. 12. Gating strategy for MMP assay, related to Supplementary Fig. 11. An example using HeLa cells without photocatalyst treatment is shown. Single cells were gated based on their FSC-A/SSC-A and FSC-A/FSC-H distributions. “MMP retained” and “MMP loss” populations were gated based on JC-1 aggregate (PE channel) and JC-1 monomer (FITC channel) fluorescence.

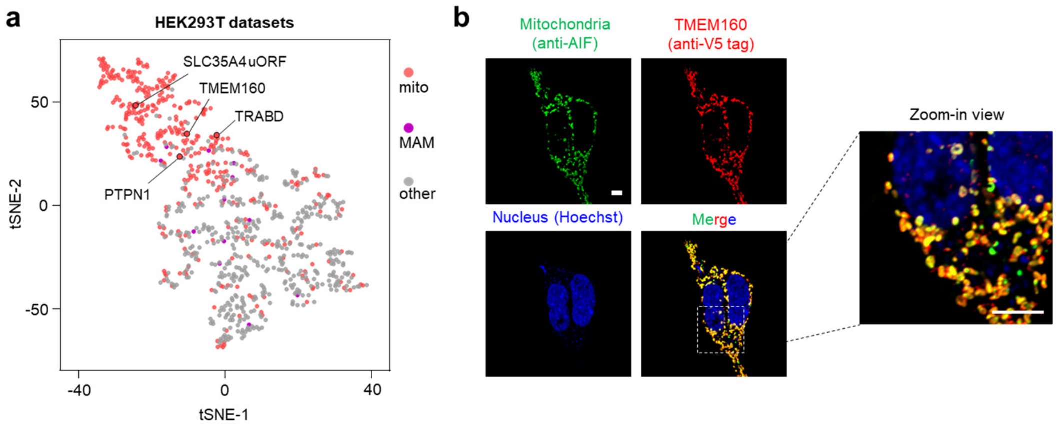

Supplementary Fig. 13. Detection of TMEM160 as a mitochondrial protein

a, Visualization of CAT-S datasets including log_2(+/− catalyst) values of HEK293T biological replicates in two dimension by t-SNE analysis. Proteins are colored based on their prior annotations in database. Red, mitochondrial localization; purple, MAM (mitochondria-associated membrane) or ER-mitochondria contact site localization. The validated "mito orphans" and TMEM160 are denoted. **b**, Imaging of overexpressed TMEM160 in HEK293T cells. Green, staining of mitochondrial marker AIF. Red, staining of overexpressed target protein by C-terminal V5 tag. Blue, staining of nucleus by Hoechst 33342. Scale bar, 5 µm. n = 2 biological replicates. Source data are provided as a Source Data file.

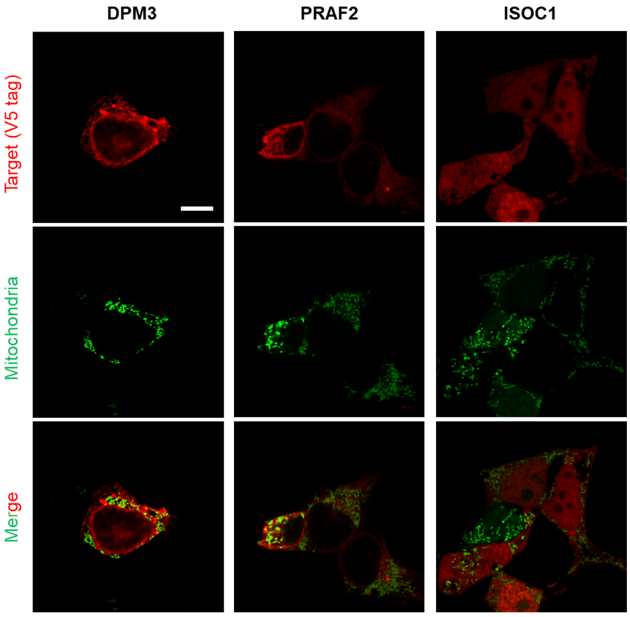

Supplementary Fig. 14. Additional imaging results for excluded "mito orphan" candidates
Imaging of overexpressed proteins in HEK293T cells. Green, mitochondria-located GFP as marker. Red, staining of overexpressed target protein by C-terminal V5 tag. Blue, staining of nucleus by Hoechst 33342. Scale bar, 5 µm. $n = 2$ biological replicates.

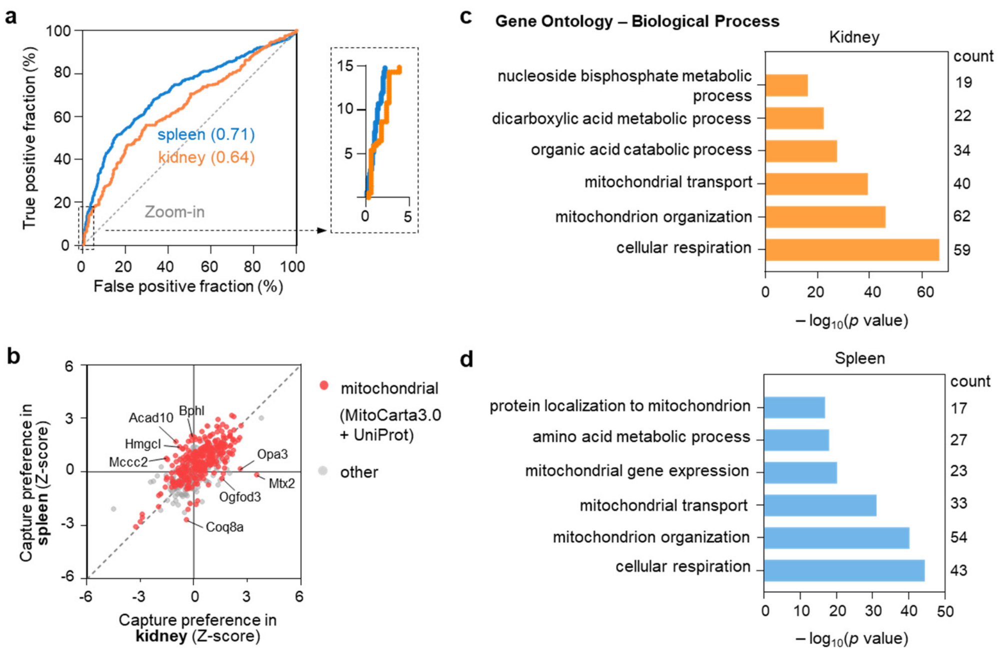

Supplementary Fig. 15. Additional analysis of the captured proteomes from mouse tissues

a, ROC curves of CAT-S data for detecting annotated mitochondrial proteins. Orange line, kidney. Blue line, spleen. **b**, Correlation of capture preferences of individual proteins by CAT-S in kidney and spleen, which were defined as Z-scored value of $\log_2$(+/− catalyst) for each protein. **c-d**, Gene Ontology (Biological Process) analysis for of the captured mitochondrial proteomes from kidney (**c**) and spleen (**d**) cells. Top six enriched biological process terms are shown, with p values calculated by cumulative hypergeometric test. Source data are provided as a Source Data file.

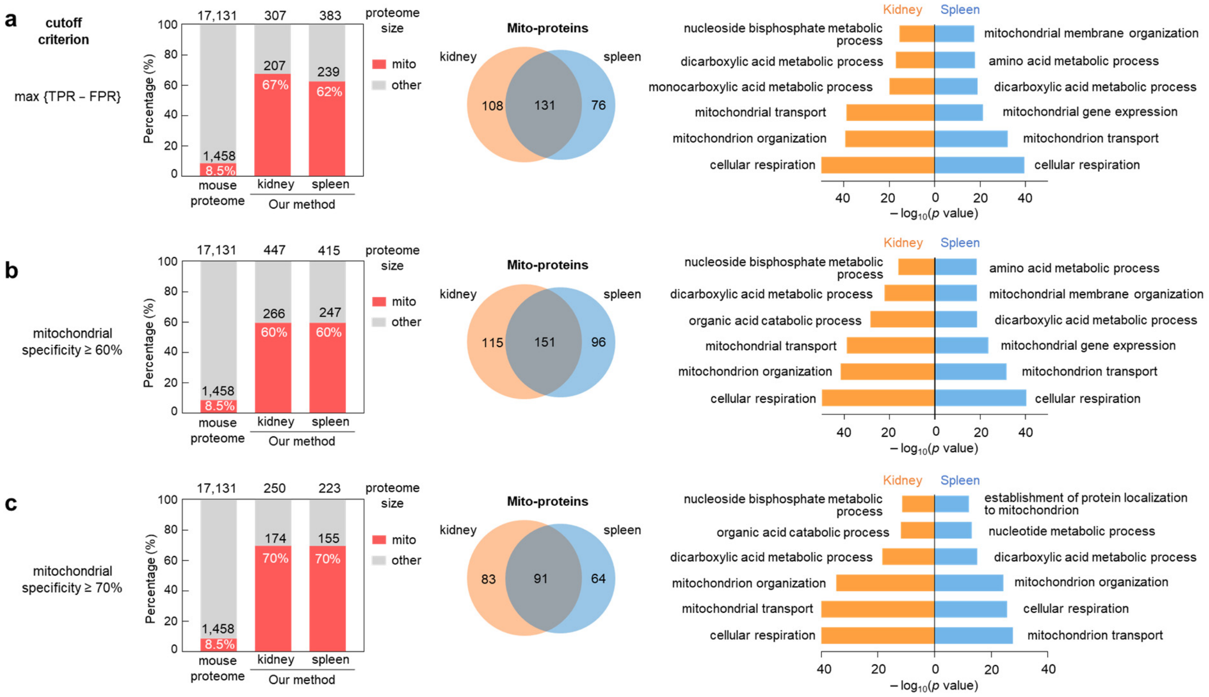

Supplementary Fig. 16. CAT-S mitochondrial proteome sizes and GO signatures of tissue samples with additional cutoff criteria

a, Maximization of (true-positive rate – false-positive rate) (i.e., TPR – FPR). **b**, Mitochondrial specificity ⩾ 60%. **c**, Mitochondrial specificity ⩾ 70%. Left column, mitochondrial specificity analysis showing the fraction of proteome with prior mitochondrial annotation in database. Middle column, Venn diagram showing the overlap between captured mitochondrial proteomes. Right column, GO Biological Process enrichment analysis of the captured mitochondrial proteomes. Top six representative terms were shown, with *p* values calculated by cumulative hypergeometric test.

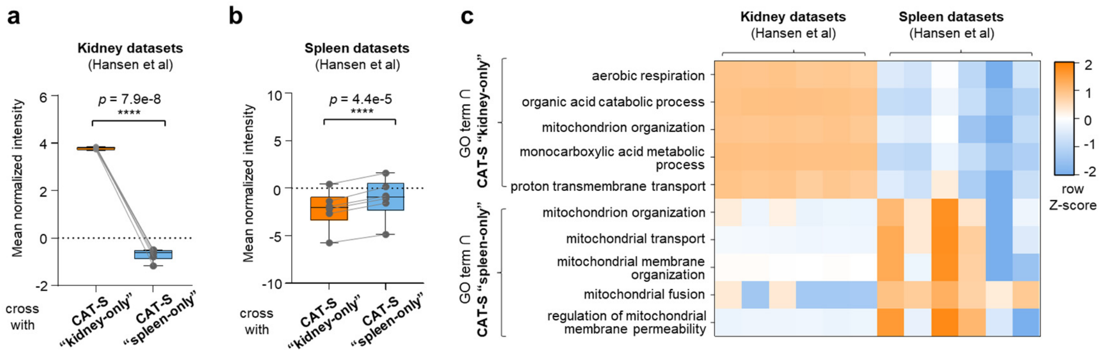

Supplementary Fig. 17. Cross-analysis with published datasets for CAT-S kidney* and spleen* proteomes

"kidney-only" and "spleen-only" refer to the proteins detected only in the post-cutoff kidney and spleen proteomes by CAT-S (see Fig. 5), related to the mitochondrial features of kidney and spleen respectively. The published datasets (Hansen et al)[7] contain the high-coverage mitochondrial proteomic data (by organelle isolation) for tissue samples from 6 mice. **a-b**, Mean normalized intensity of "kidney-only" and "spleen-only" proteins in the published kidney or spleen datasets. Overall, the results indicated that the "kidney-only" proteins are typically more significant in the kidney datasets (**a**), while the "spleen-only" proteins are typically more significant in the spleen datasets (**b**). Data points of the same sample are connected by line. Data are represented as box and whiskers ($n = 6$ samples). Center line denotes median value. Box spans from lower to upper quartile. Whiskers denote min and max values. ****, $p < 0.0001$ (paired two-tailed t test). **c**, Heatmap showing the mean normalized intensity (Z-scored) of "kidney-only" or "spleen-only" proteins (grouped by specific GO terms) in the published datasets. Source data are provided as a Source Data file.

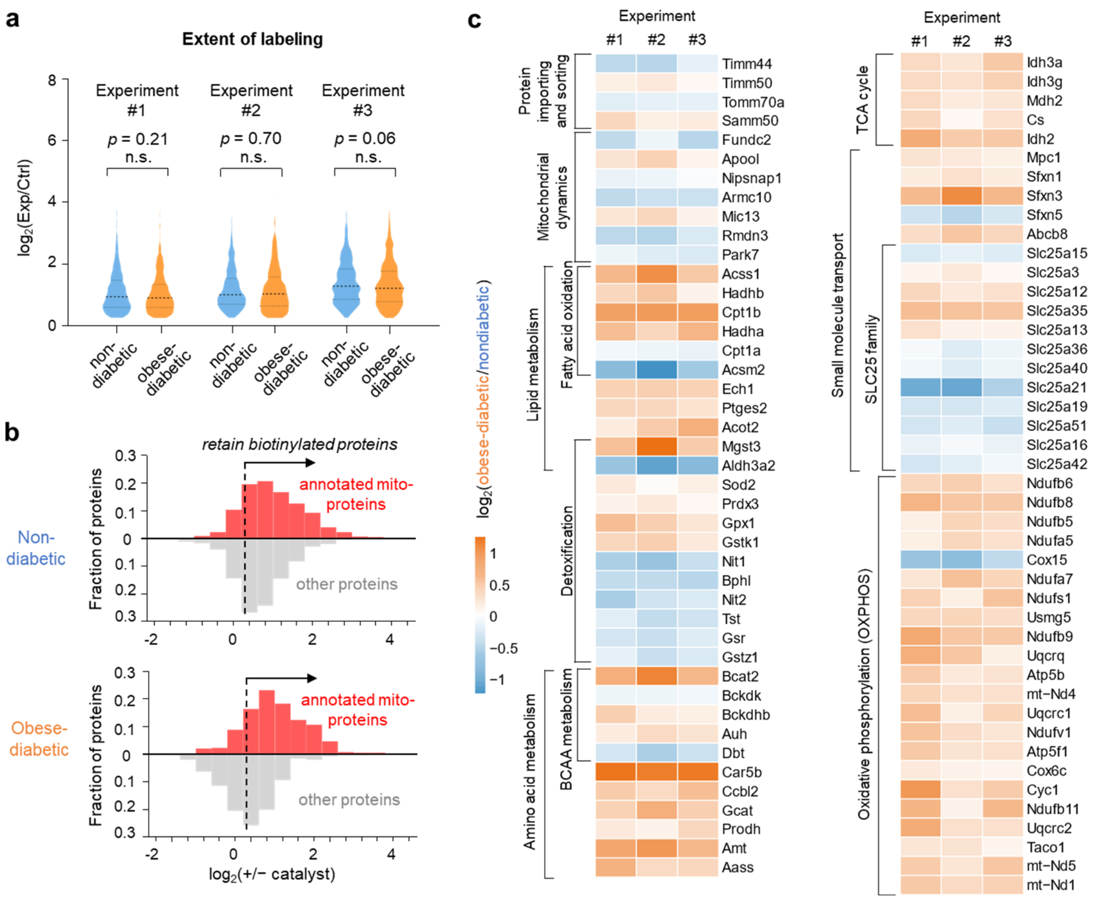

Supplementary Fig. 18. Mitochondrial proteomic signatures in obese-diabetic mouse kidney **a**, Comparison of overall extents of labeling (represented by Exp/Ctrl ratio) by CAT-S for nondiabetic and obese-diabetic kidneys, indicating the overall labeling was at same level for the both. Blue, nondiabetic. Orange, obese-diabetic. n.s., $p > 0.05$ (unpaired two-tailed *t* test). Data are presented as violin plot with median, upper quartile and lower quartile denoted as lines. **b**, Frequency distribution profiles of MS-identified proteins with (red) and without (gray) mitochondrial annotation in mouse database (MitoCarta3.0 + UniProt). A filter based on +/− catalyst ratio (set to 1.2) for further noise subtraction (non-specific proteins) while retaining the most of biotinylated proteins is illustrated. **c**, Heatmap showing regulation of proteins ($p < 0.05$) involved in major mitochondrial pathways across three independent experiments. Orange, upregulated in obese-diabetic. Blue, downregulated in obese-diabetic. Source data are provided as a Source Data file.

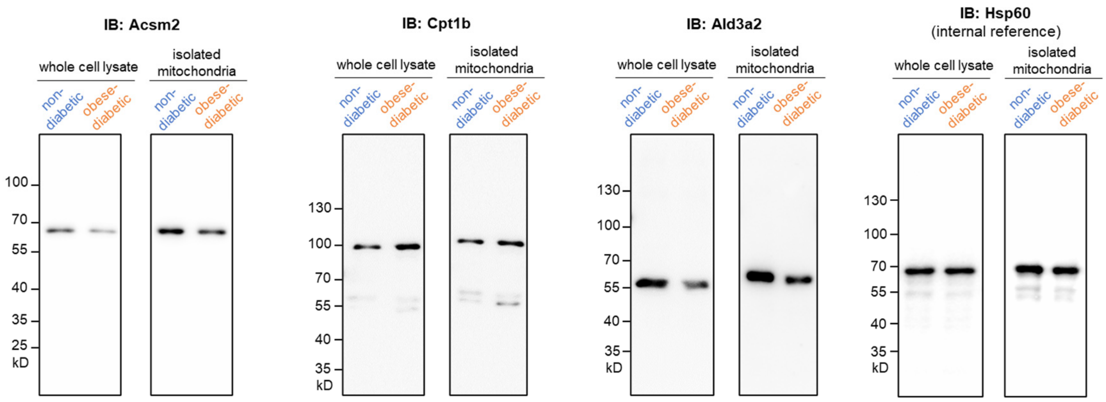

Supplementary Fig. 19. Mitochondrial proteomic signatures in obese-diabetic mouse kidney

Uncropped blot images for Ascm2, Cpt1b, Ald3a2 and Hsp60 (mitochondrial internal reference). Cell lysates and mitochondrial lysates of nondiabetic and obese-diabetic mouse kidneys were used as samples. n = 3 biological replicates.

NMR SPECTRA

1a: ^{1}H NMR (400 MHz, Chloroform-d)

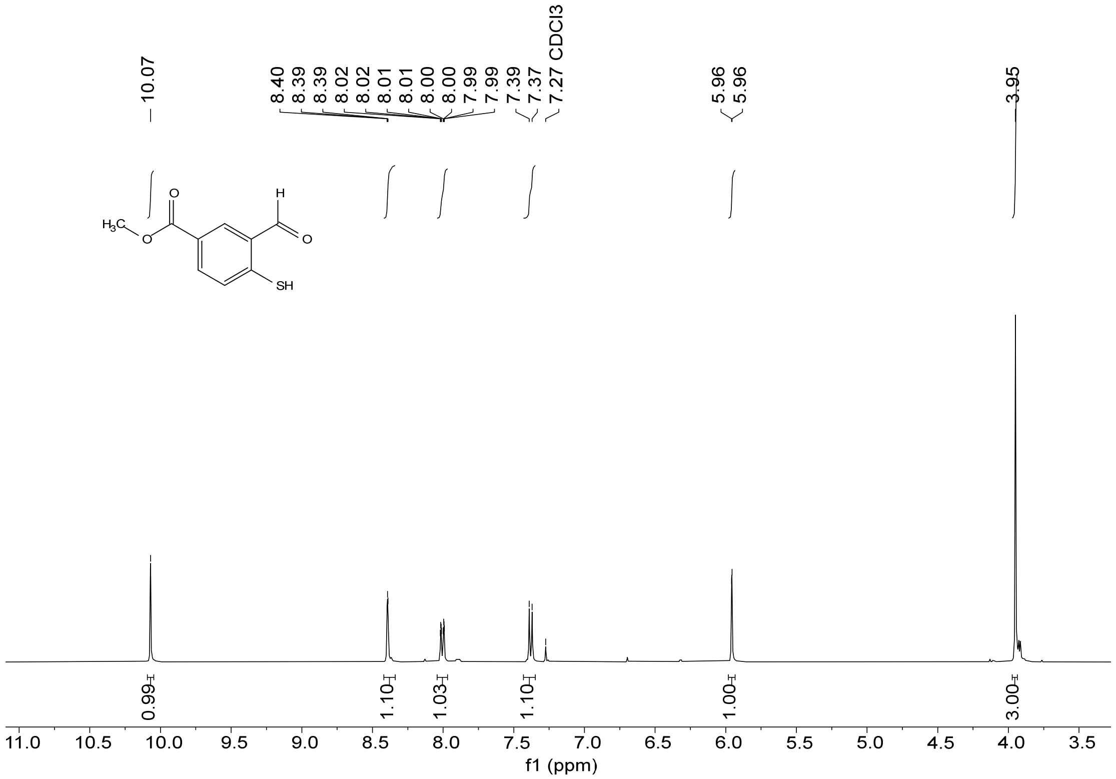

1a: ^{13}C NMR (126 MHz, Chloroform-*d*)

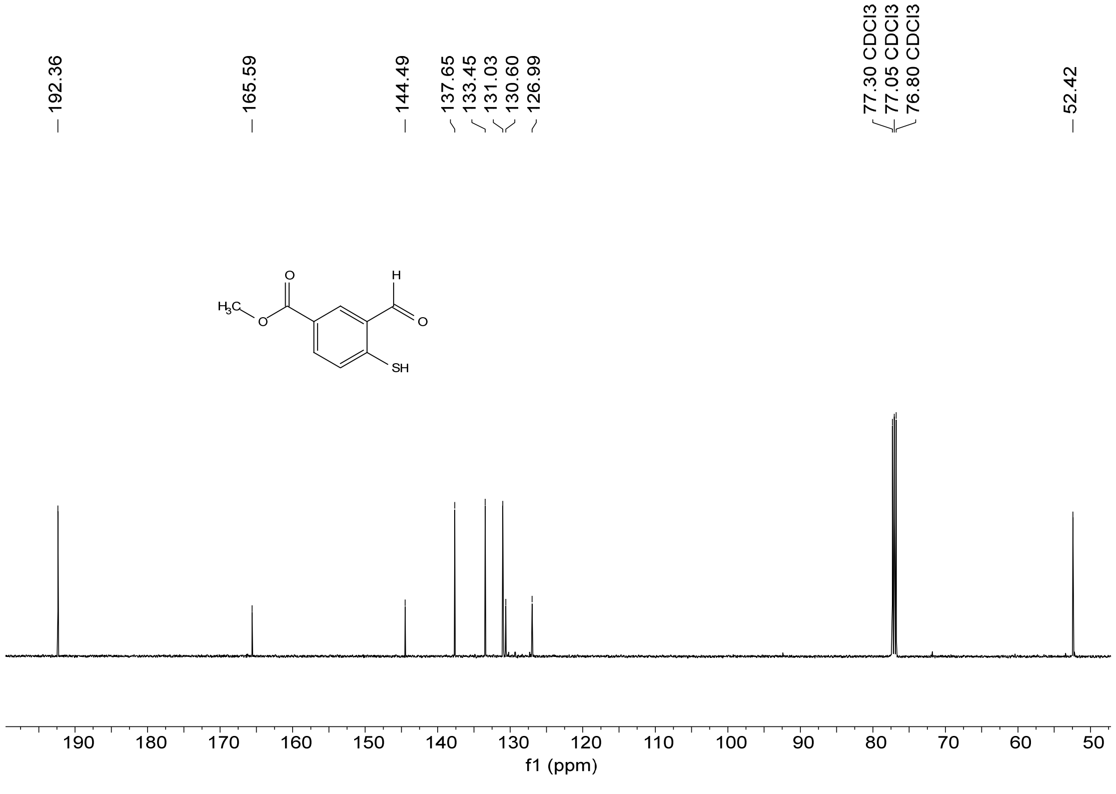

2b: ^{1}H NMR (400 MHz, Chloroform-d)

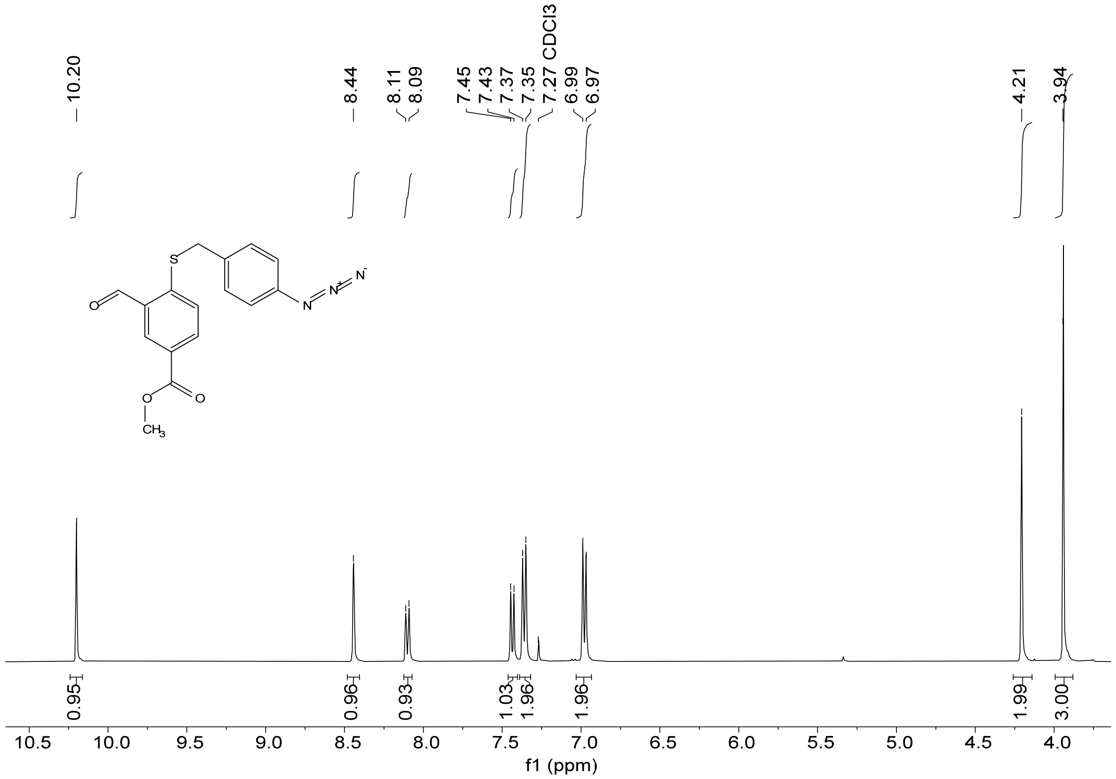

2b: ^{13}C NMR (151 MHz, Chloroform-*d*)

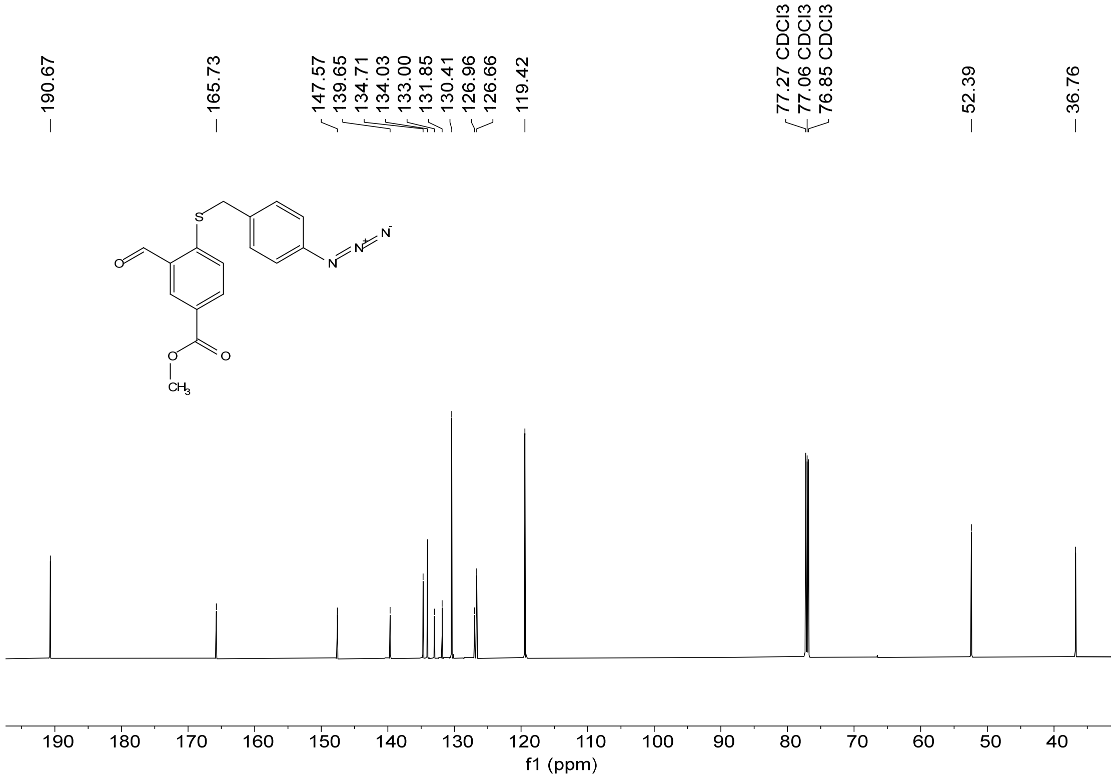

3a: ^{1}H NMR (400 MHz, Chloroform-d)

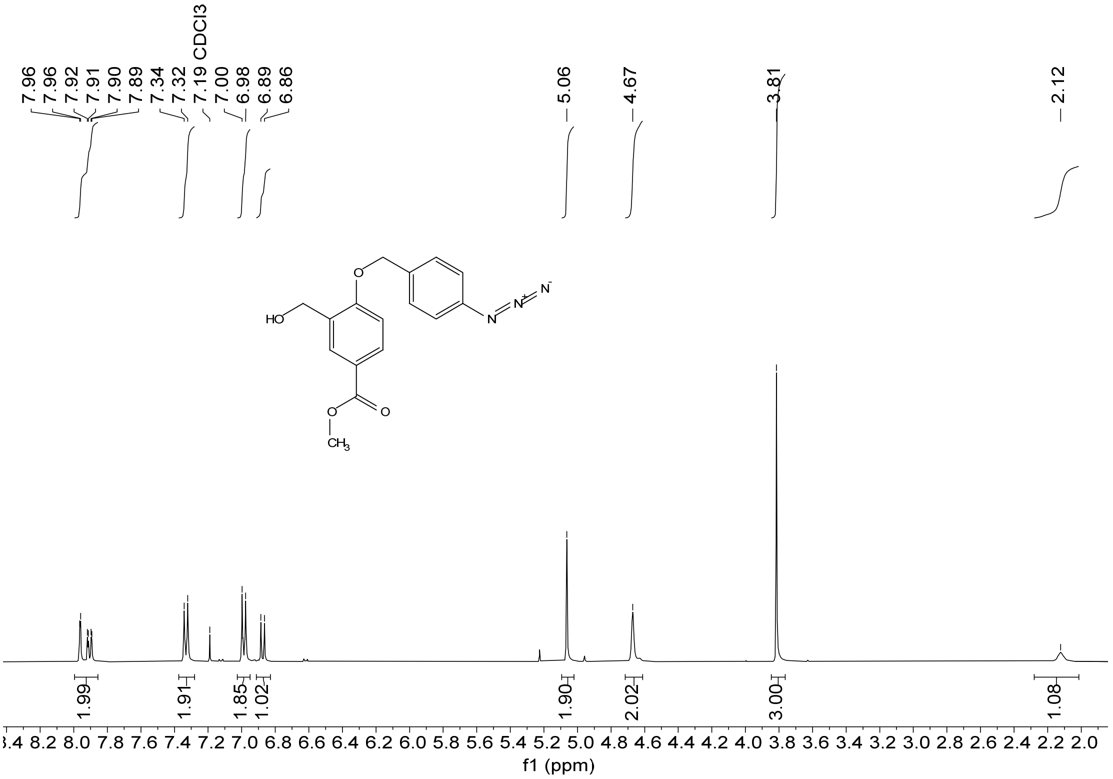

3a: ^{13}C NMR (151 MHz, Chloroform-*d*)

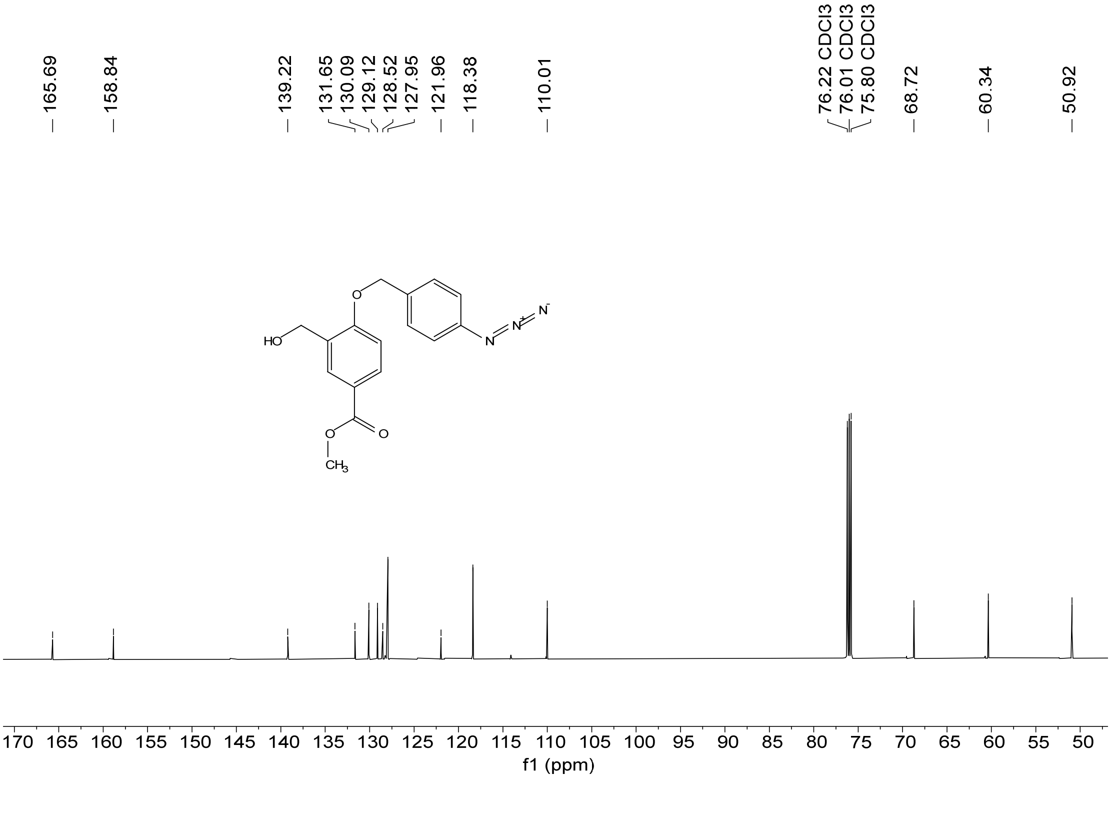

3b: ^{1}H NMR (400 MHz, Chloroform-d)

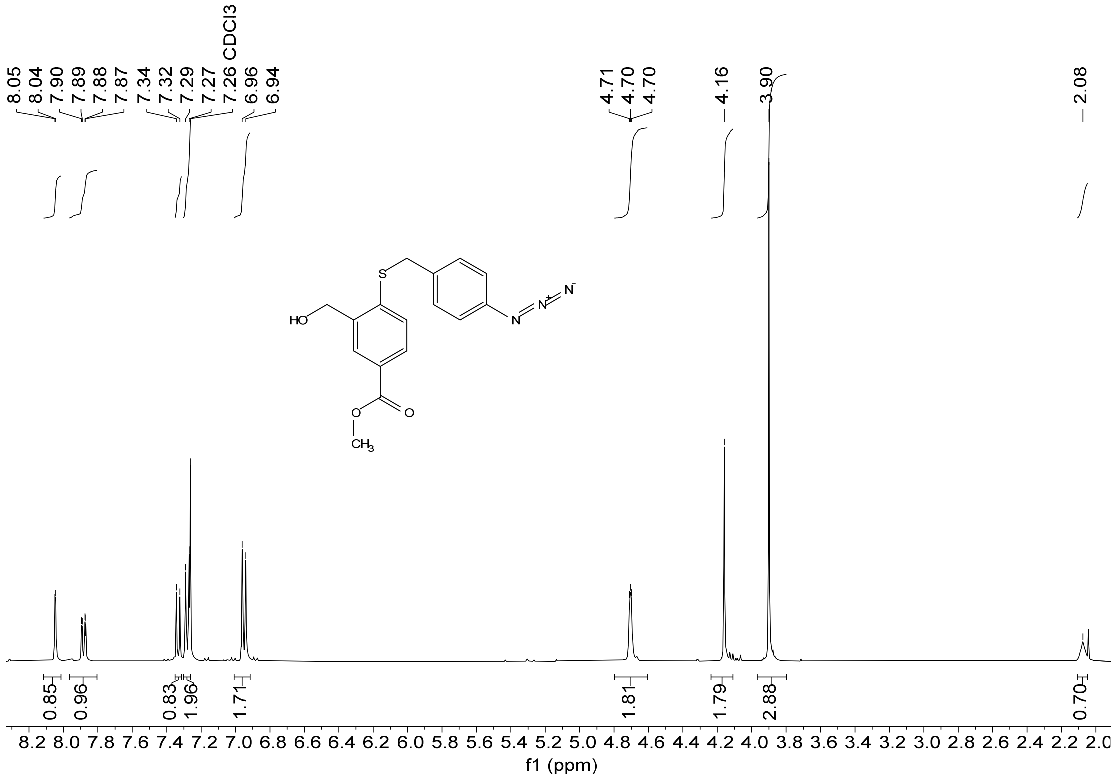

3b: ^{13}C NMR (151 MHz, Chloroform-*d*)

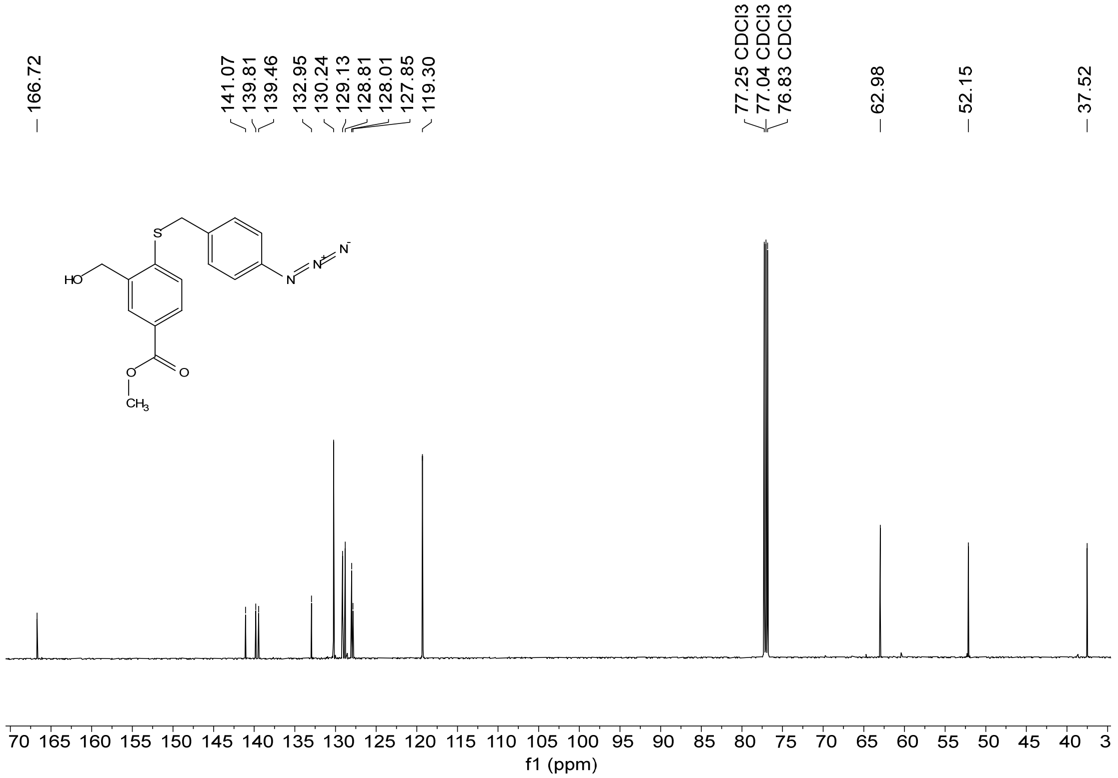

4a: ^{1}H NMR (400 MHz, Chloroform-d)

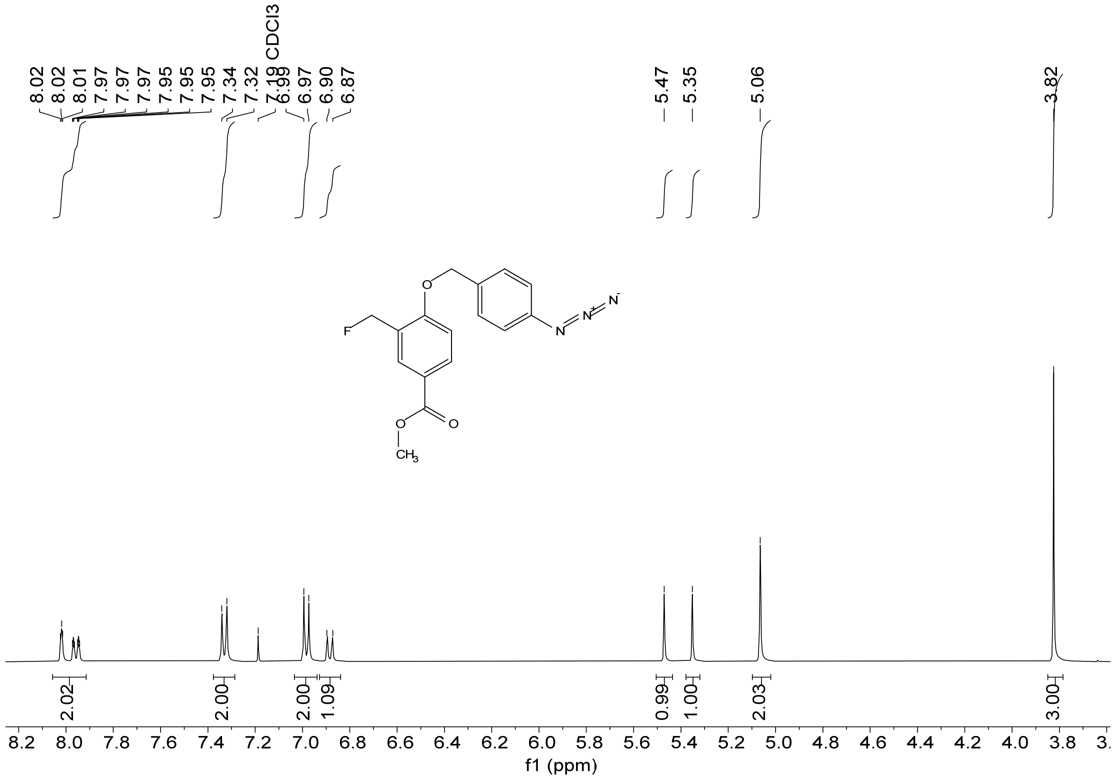

4a: ^{13}C NMR (151 MHz, Chloroform-*d*)

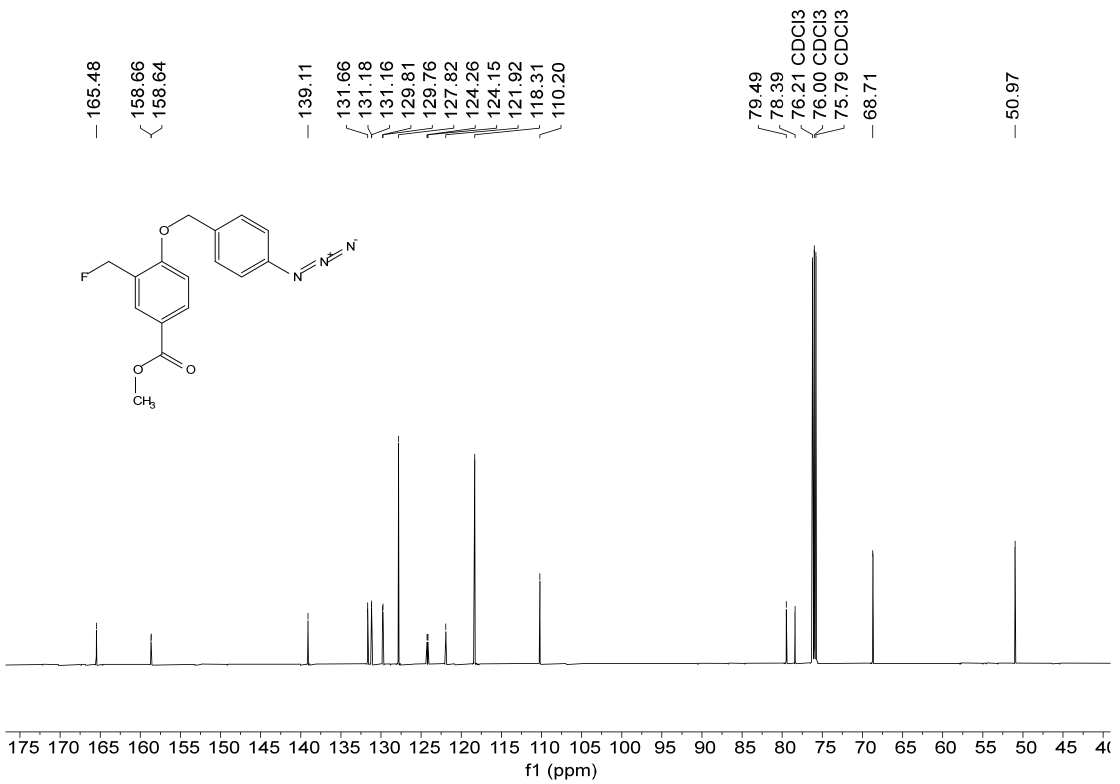

4b: ^{1}H NMR (400 MHz, Chloroform-d)

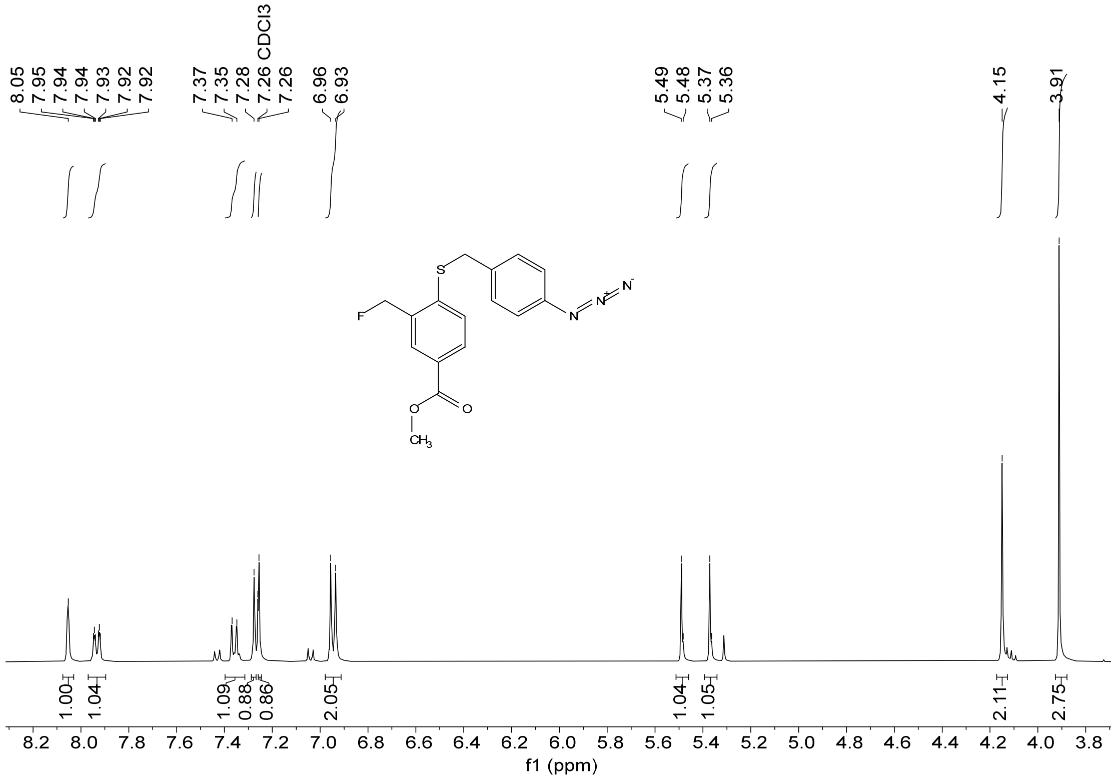

4b: ^{13}C NMR (151 MHz, Chloroform-*d*)

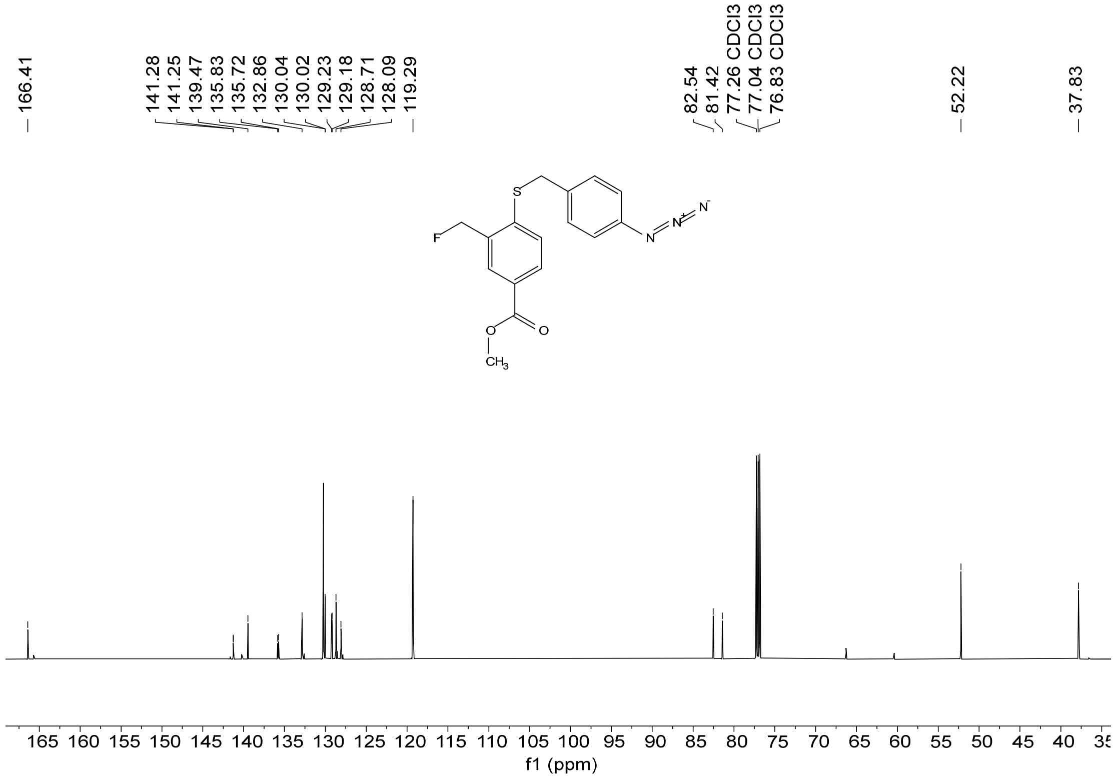

4d: ^{1}H NMR (400 MHz, Chloroform-d)

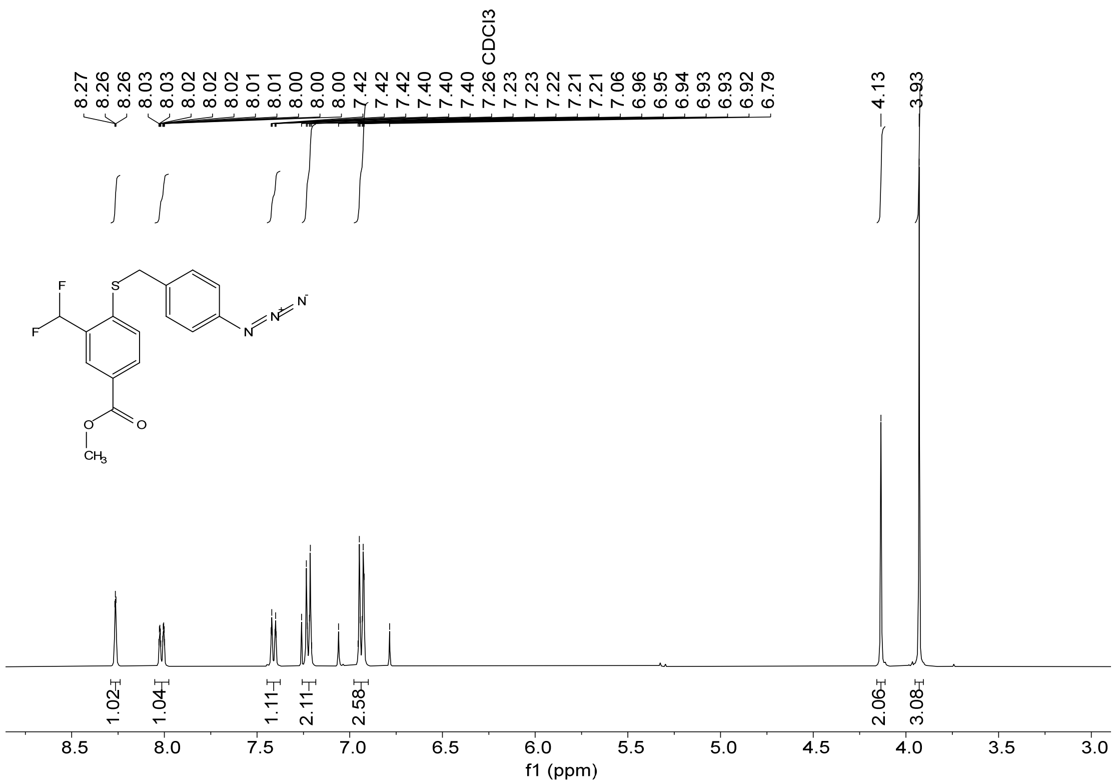

4d: ^{13}C NMR (126 MHz, Chloroform-*d*)

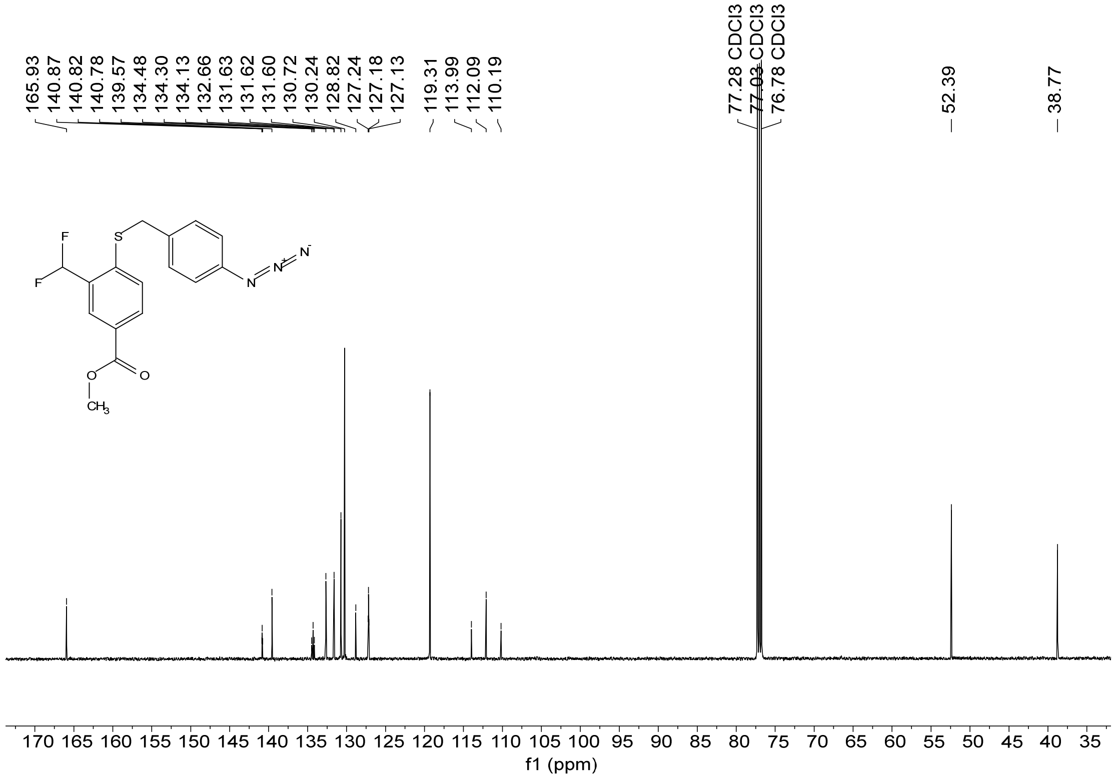

4d: ^{19}F NMR (471 MHz, Chloroform-*d*)

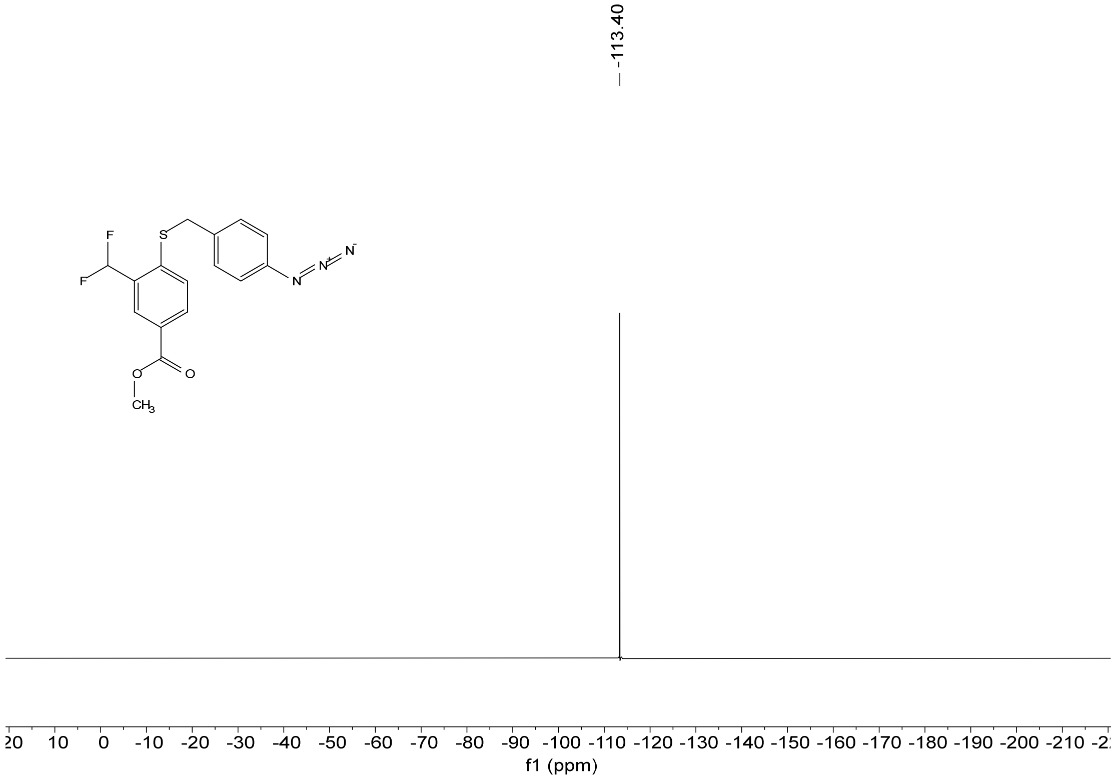

6a: ^{1}H NMR (400 MHz, Chloroform-d)

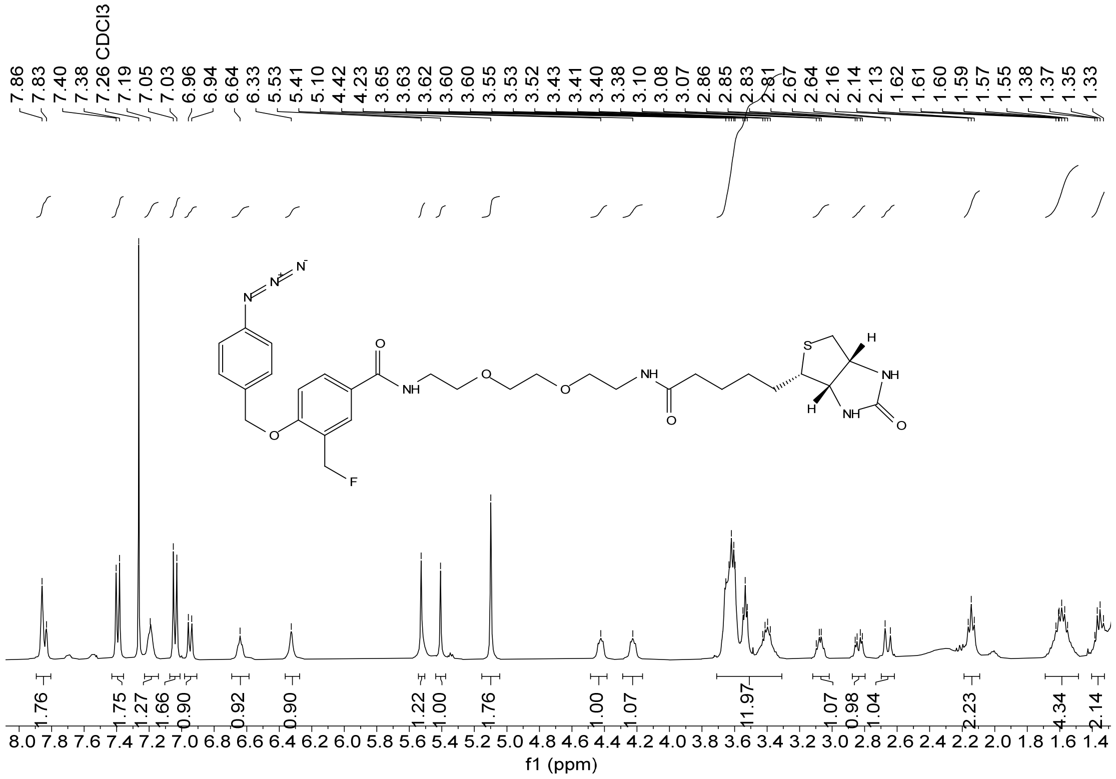

6a: ^{13}C NMR (151 MHz, Chloroform-*d*)

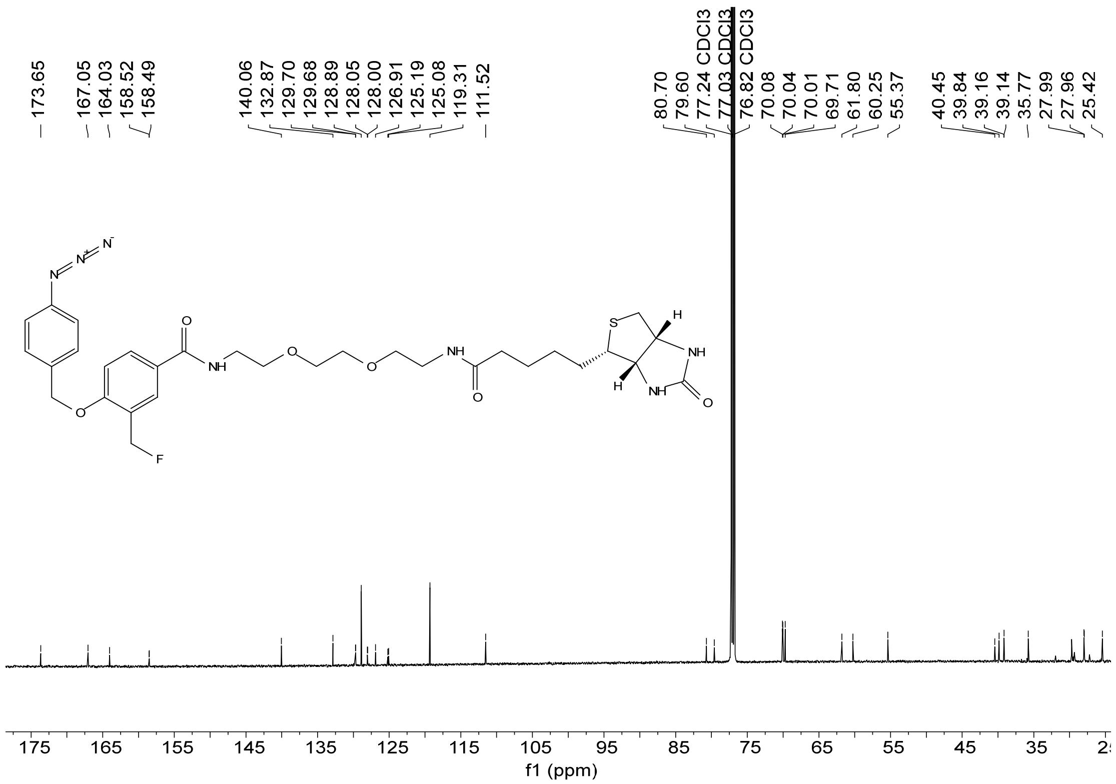

6b: ^{1}H NMR (400 MHz, Chloroform-d)

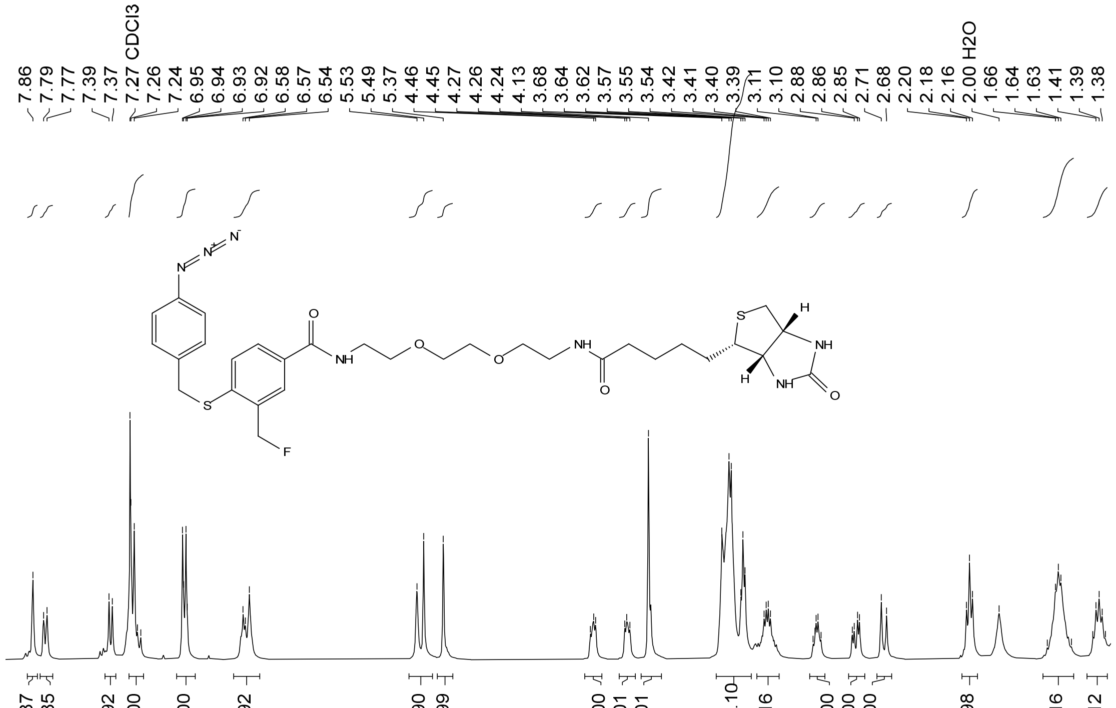

6b: ^{13}C NMR (126 MHz, Chloroform-*d*)

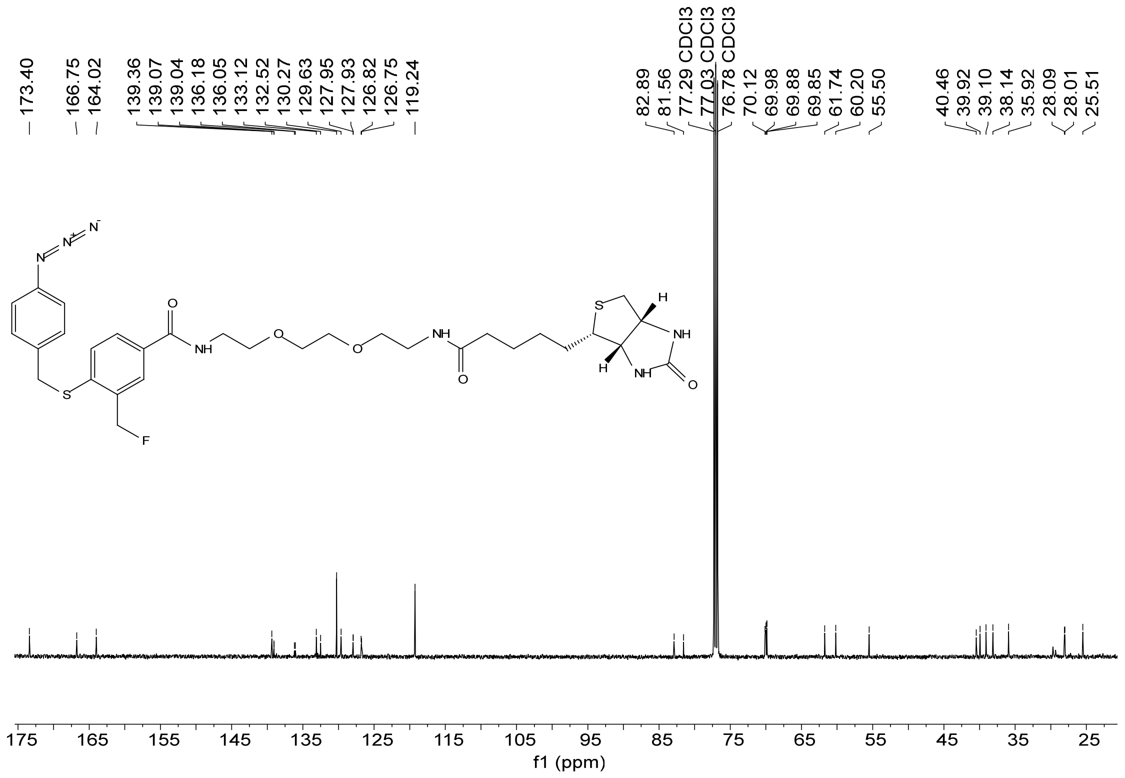

6c: ^{1}H NMR (400 MHz, Chloroform-d)

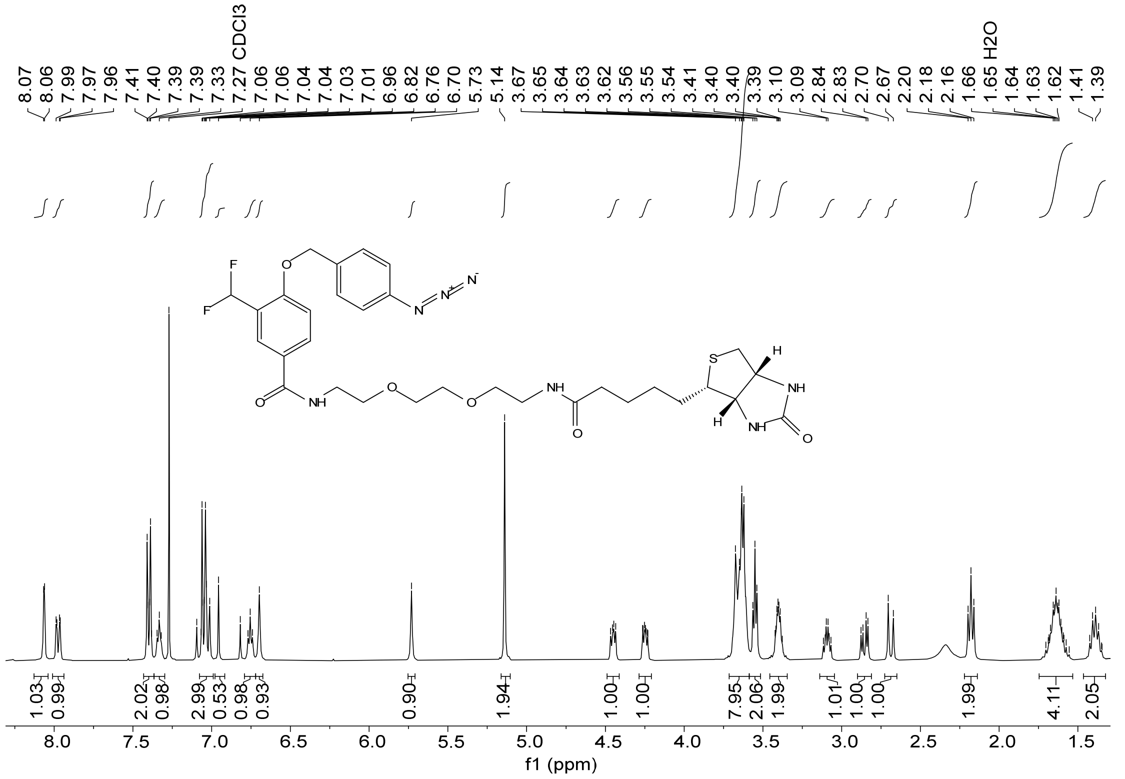

6c: ^{13}C NMR (126 MHz, Chloroform-*d*)

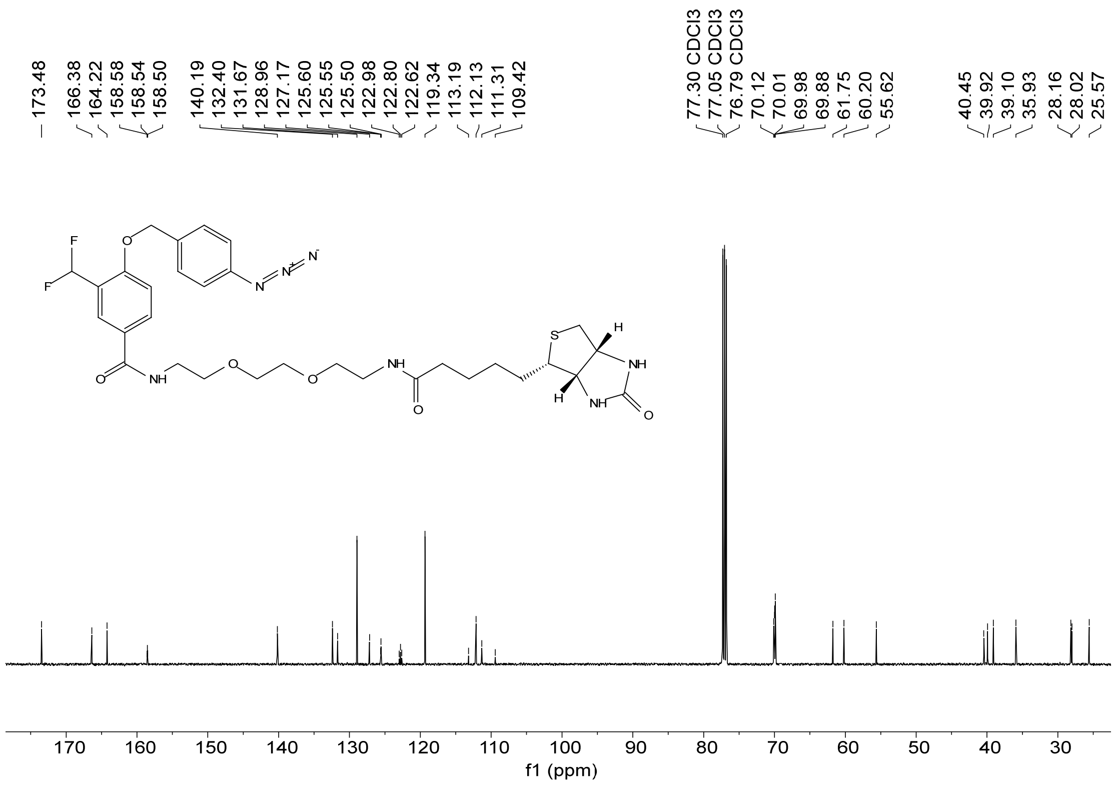

6c: ^{19}F NMR (471 MHz, Chloroform-*d*)

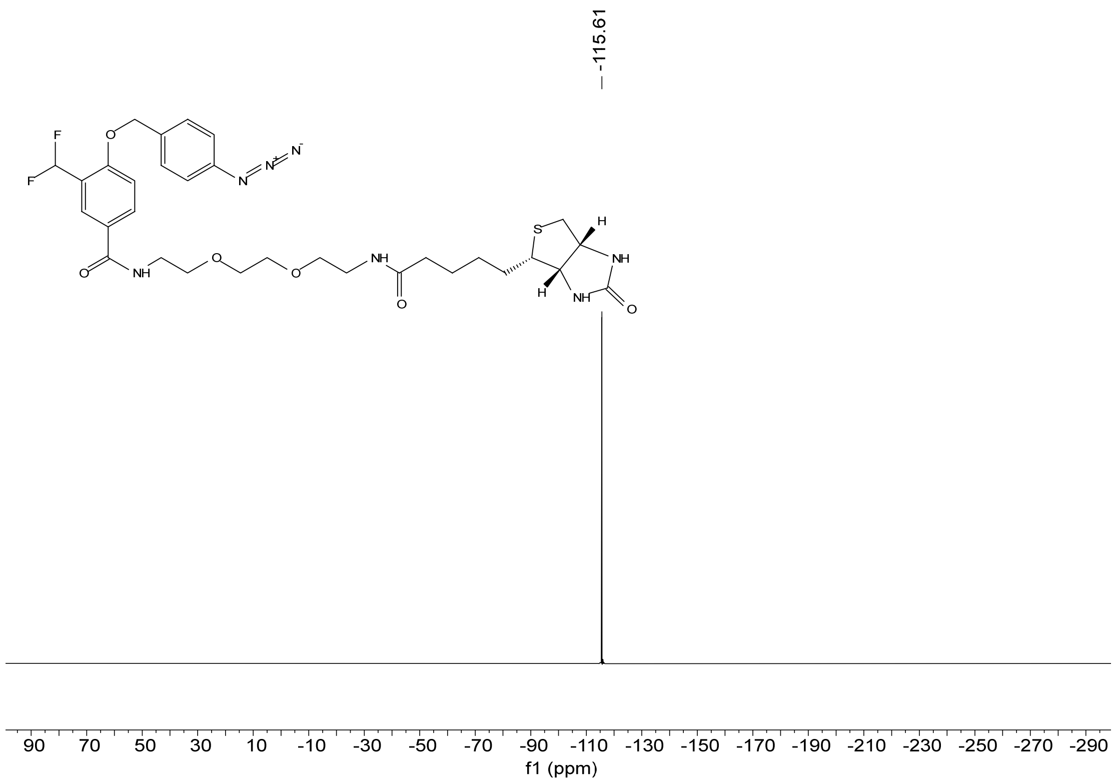

6d: ^{1}H NMR (400 MHz, Chloroform-d)

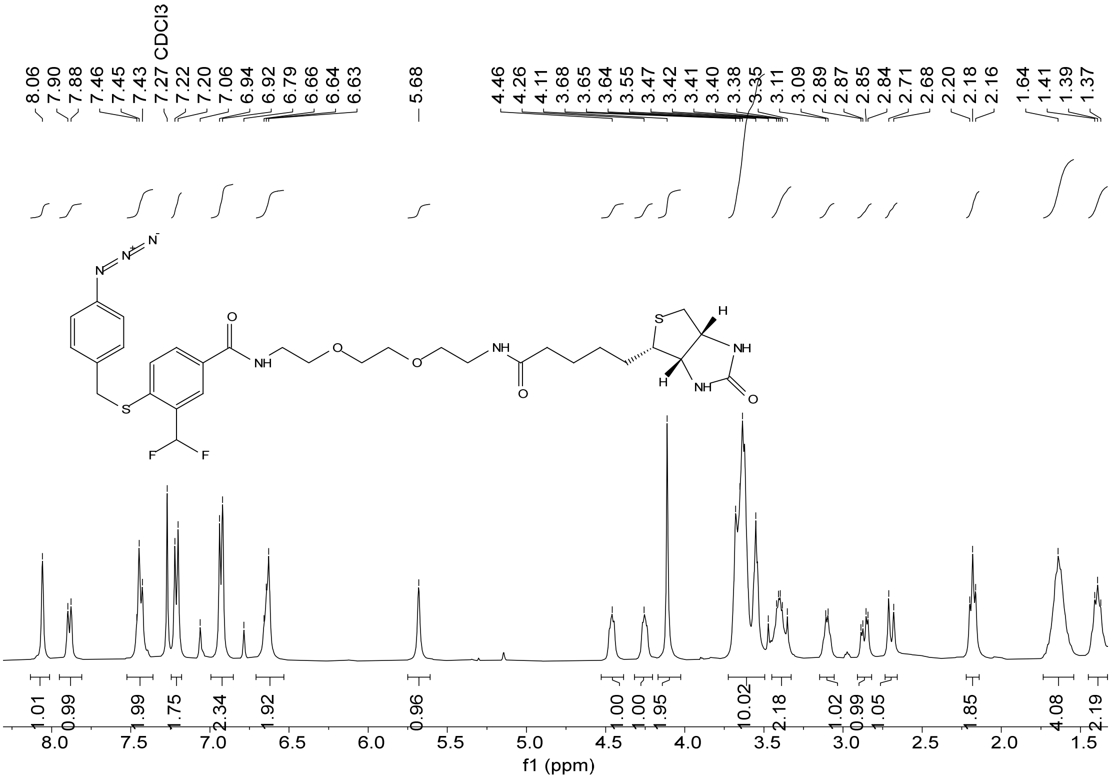

6d: ^{13}C NMR (151 MHz, Chloroform-*d*)

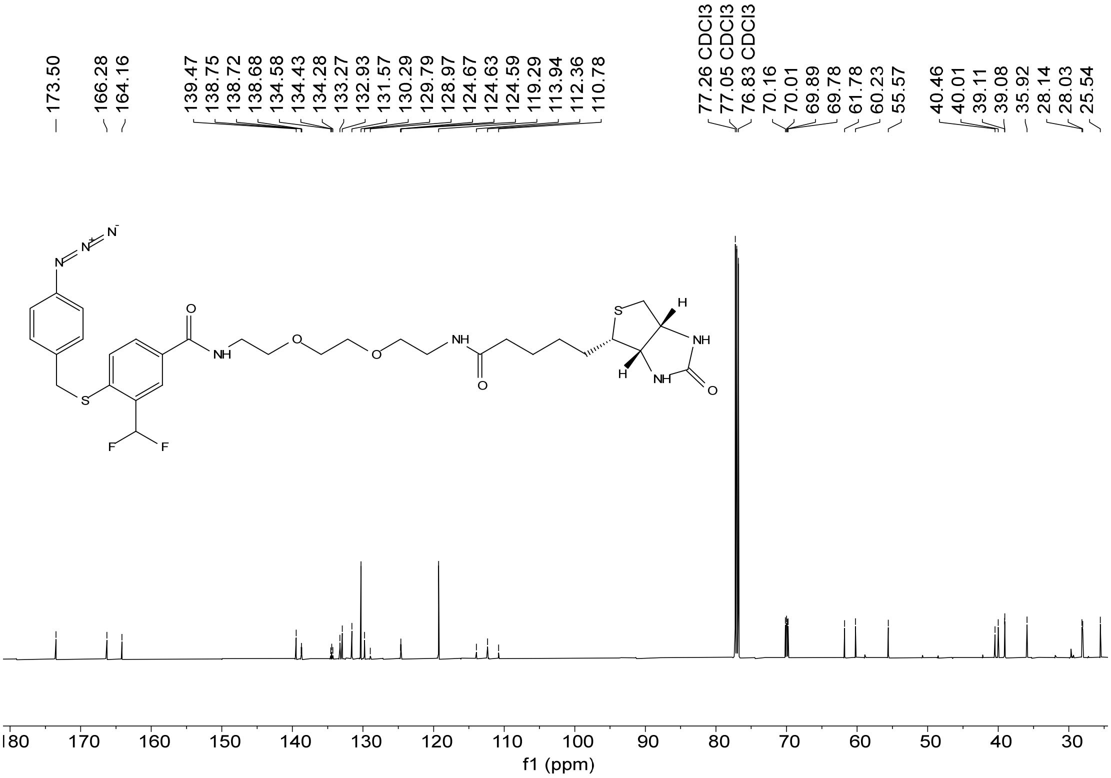

6d: ^{19}F NMR (565 MHz, Chloroform-*d*)

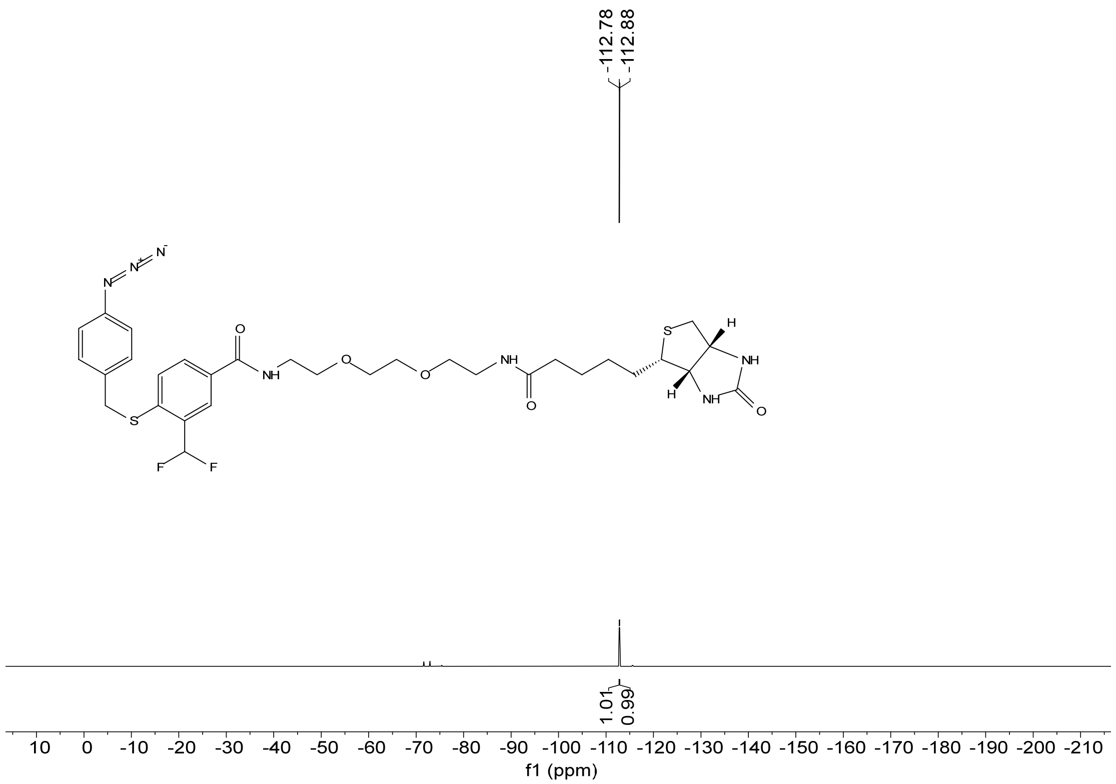

6d': ^{1}H NMR (400 MHz, Chloroform-d)

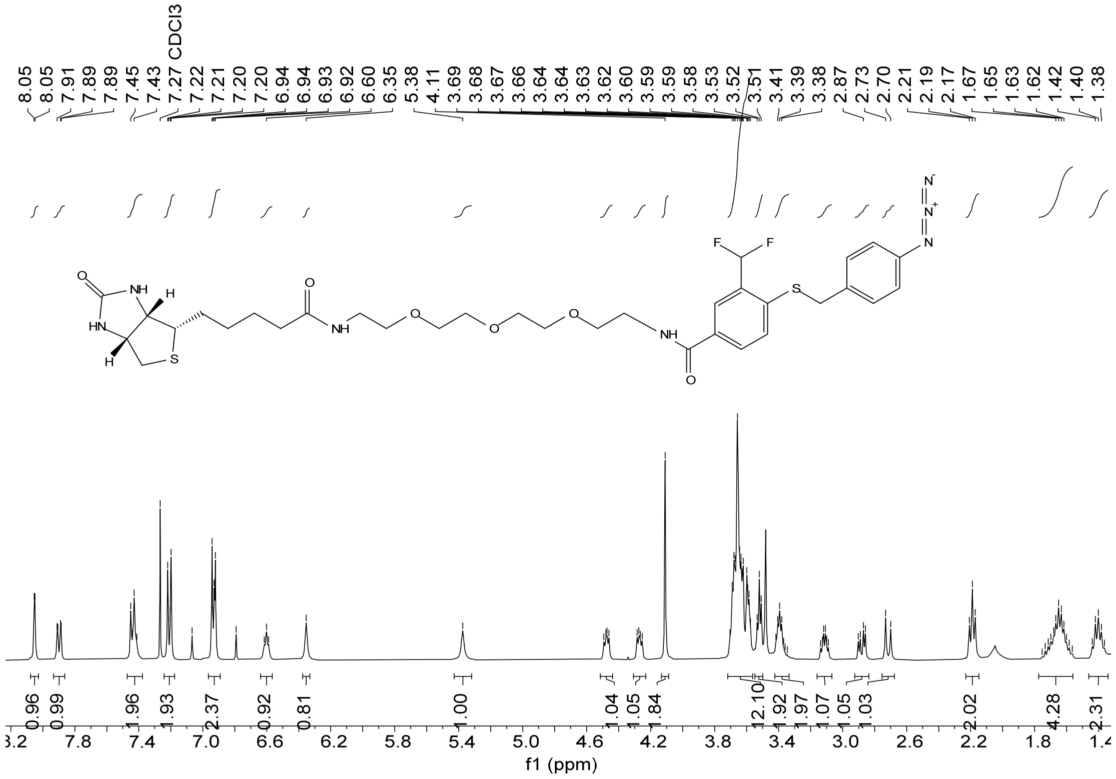

6d': ^{13}C NMR (126 MHz, Chloroform-*d*)

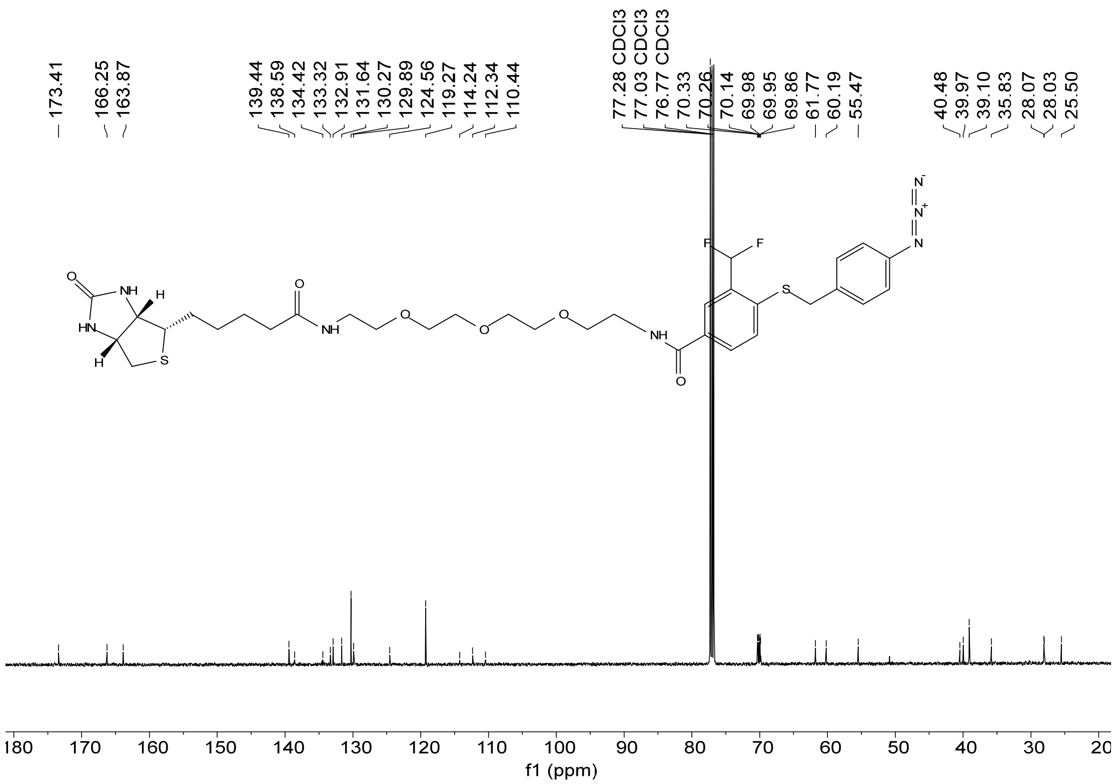

6d': ^{19}F NMR (471 MHz, Chloroform-*d*)

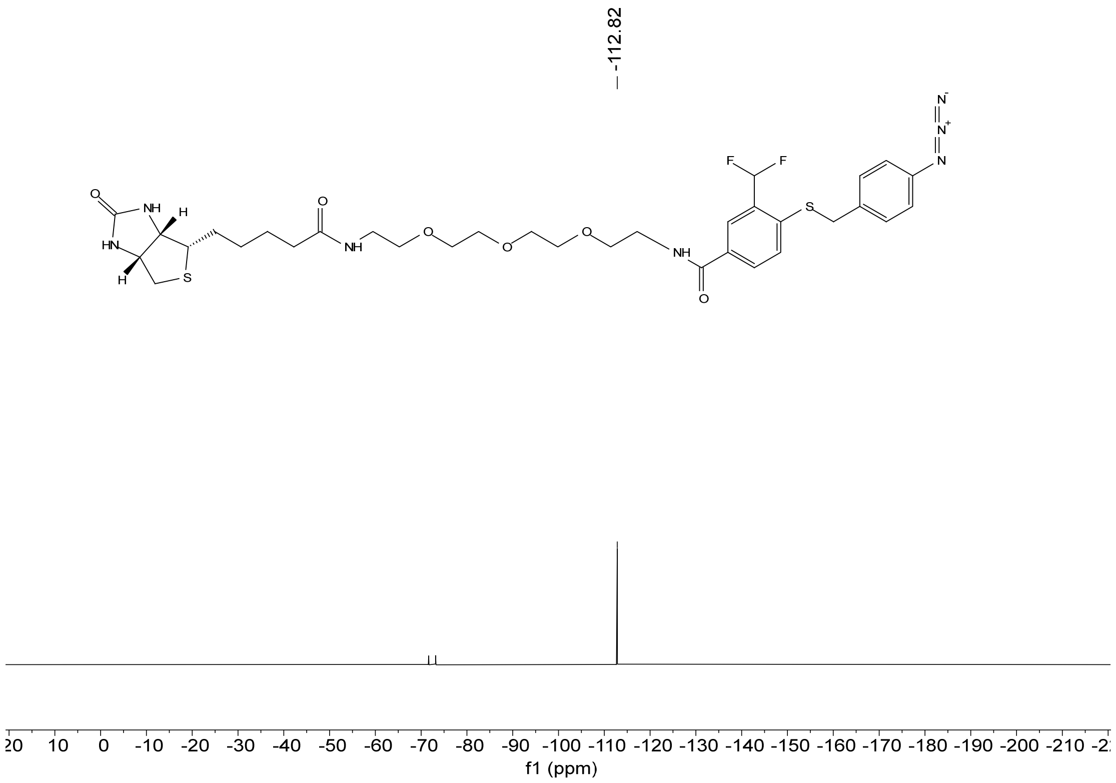

6e: ^{1}H NMR (400 MHz, Chloroform-d)

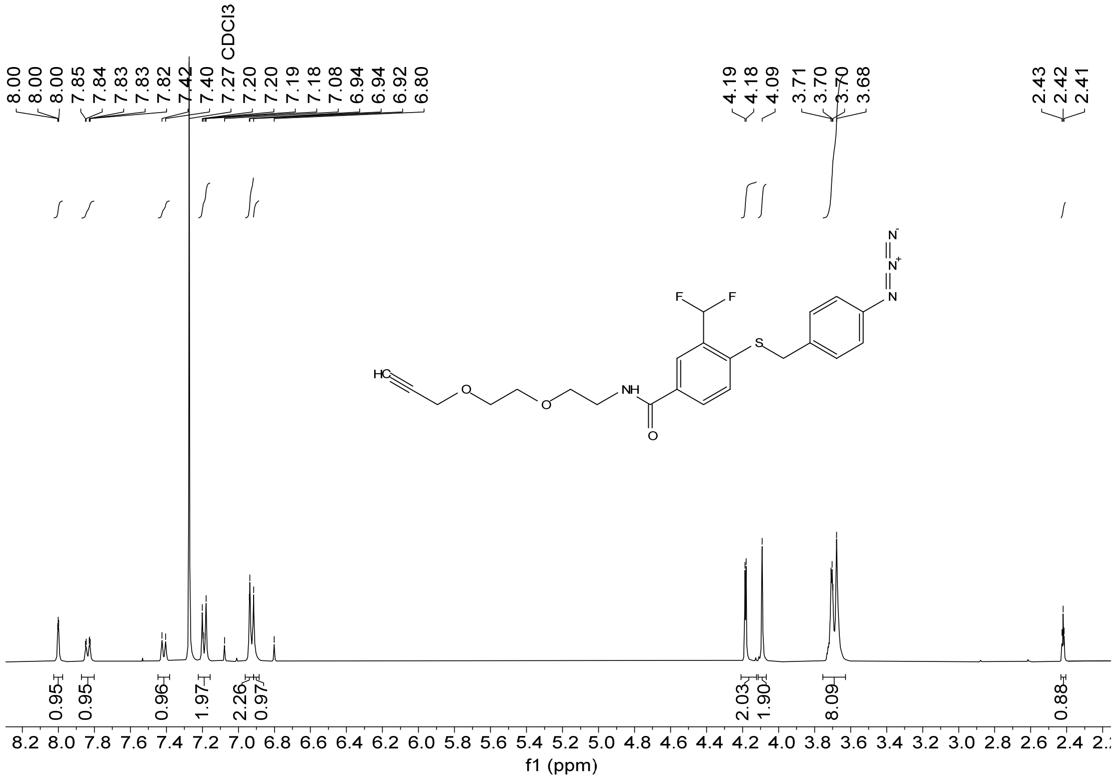

6e: ^{13}C NMR (151 MHz, Chloroform-*d*)

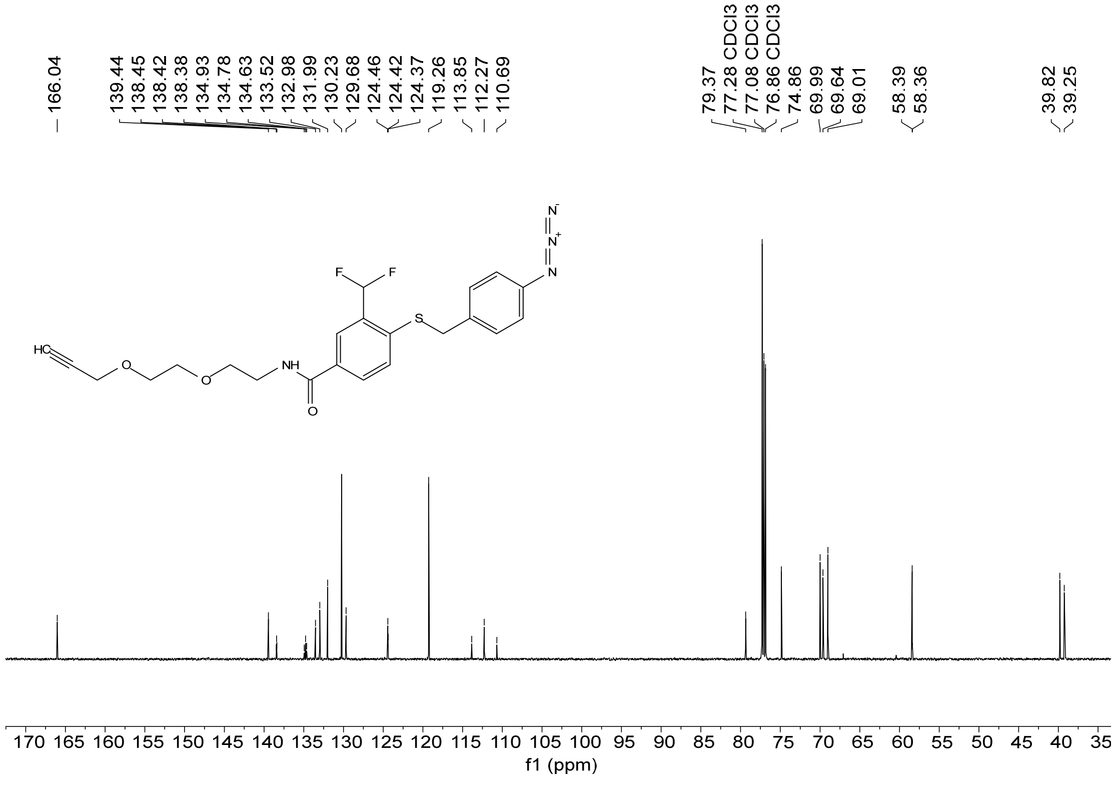

6e: ^{19}F NMR (565 MHz, Chloroform-*d*)

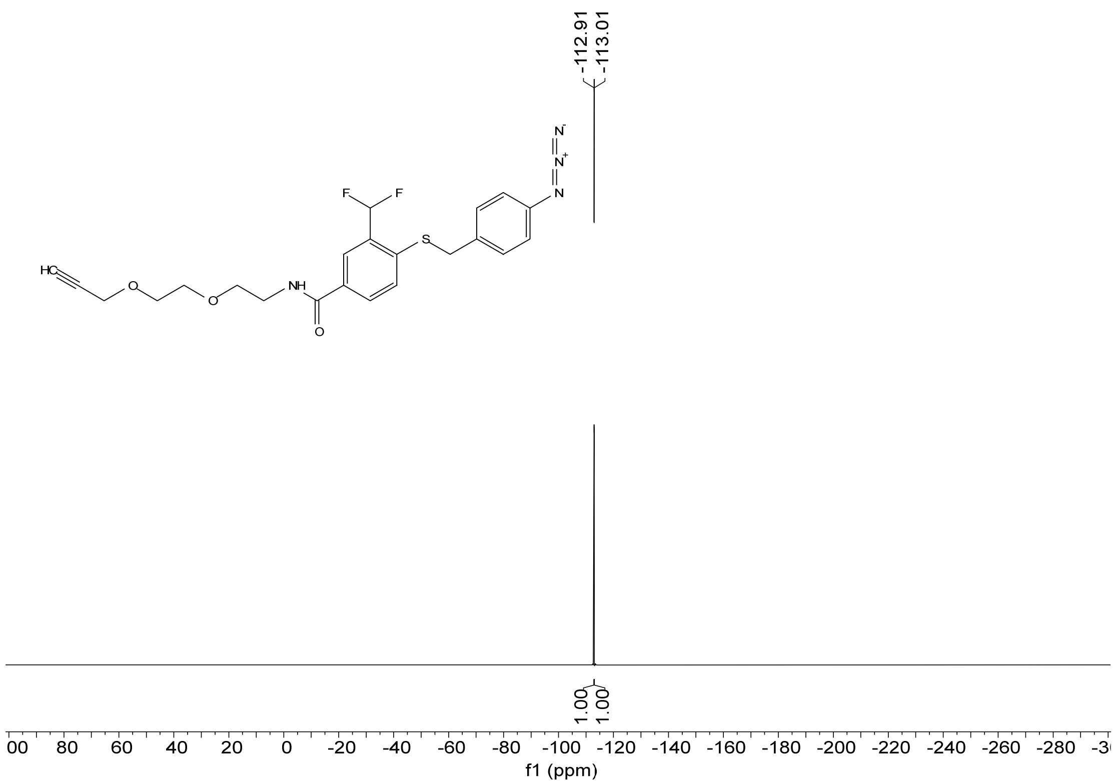

2bb: ^{1}H NMR (400 MHz, Chloroform-d)

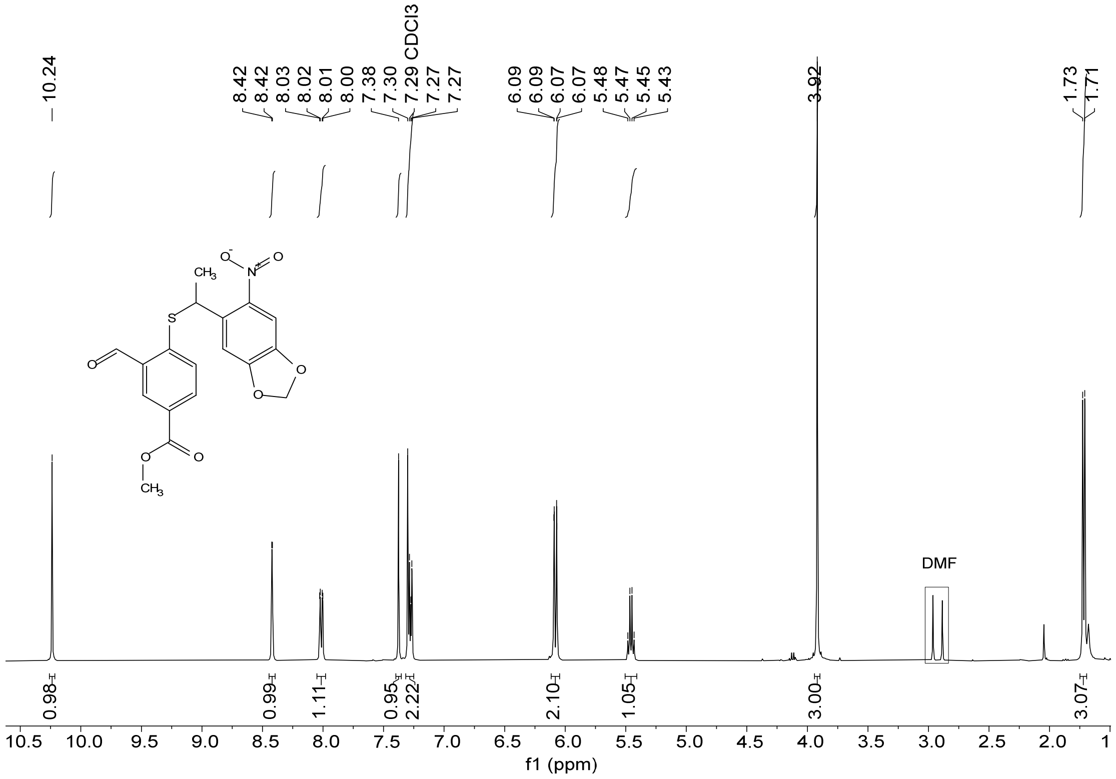

2bb: ^{13}C NMR (126 MHz, Chloroform-*d*)

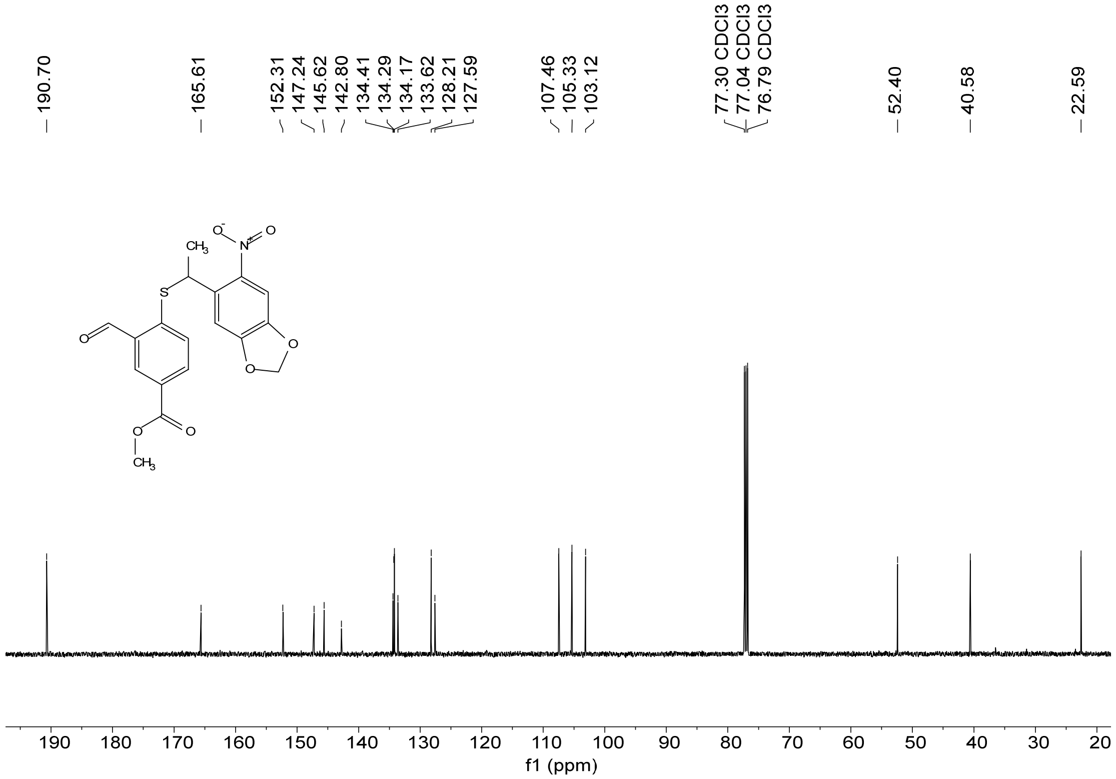

4dd: ^{1}H NMR (400 MHz, Chloroform-d)

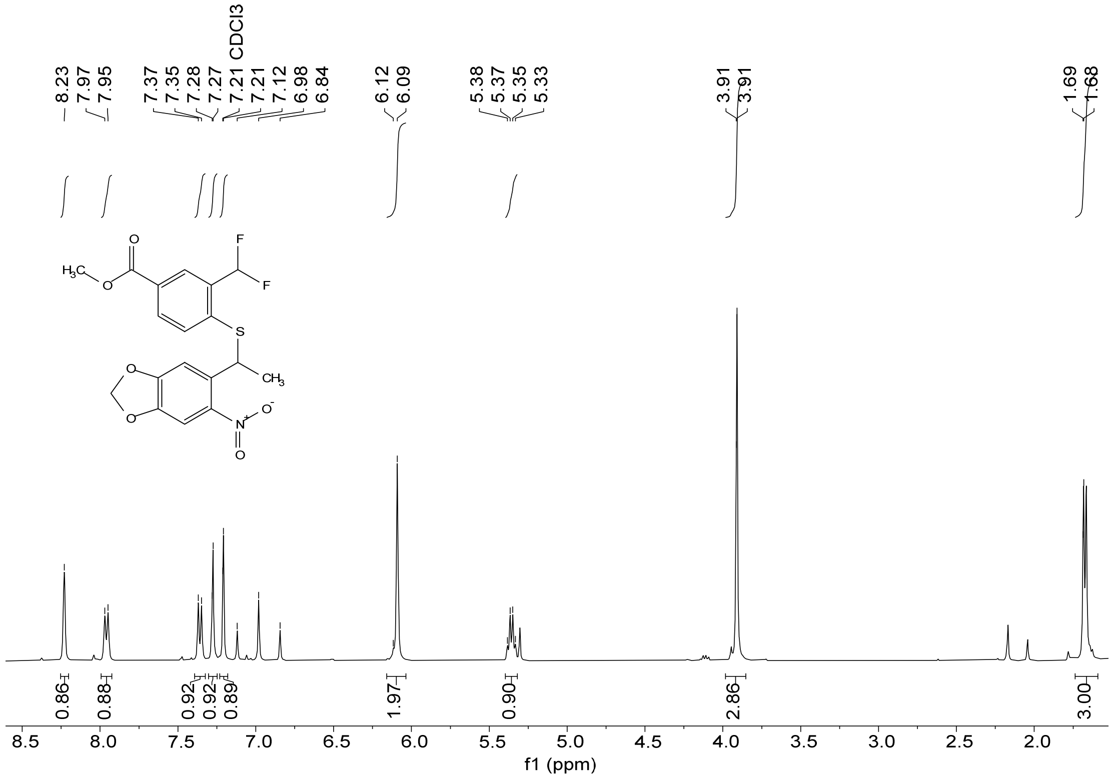

4dd: ^{13}C NMR (126 MHz, Chloroform-*d*)

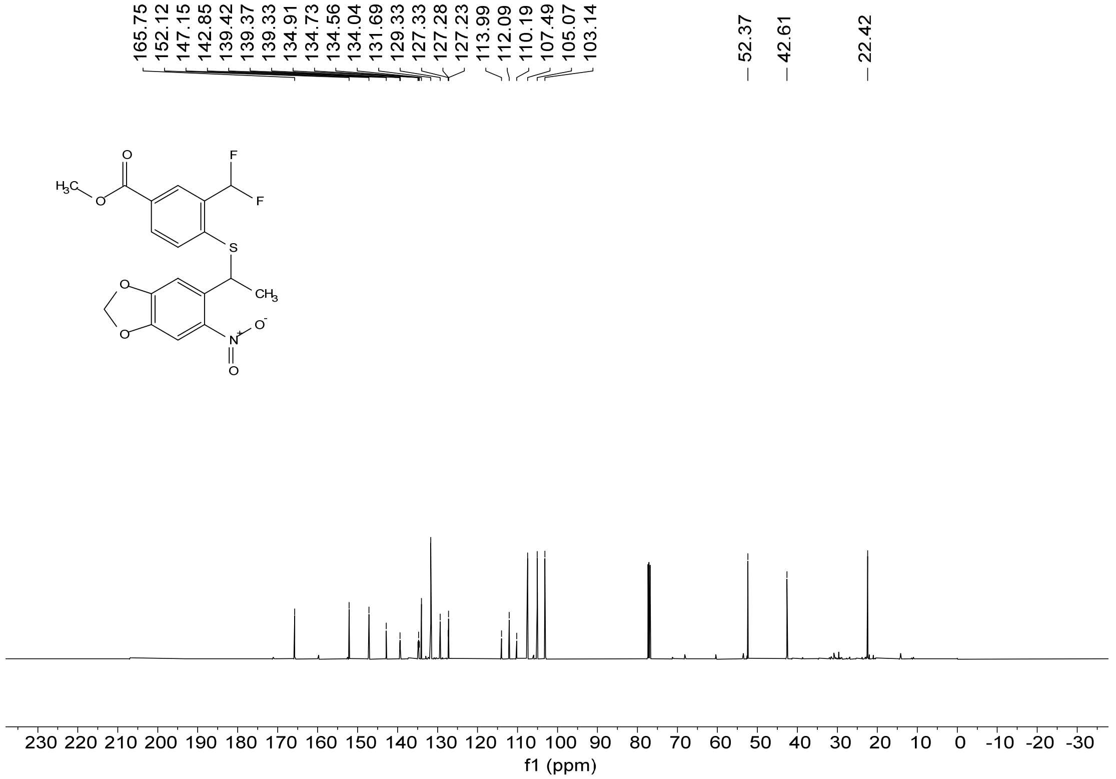

6dd: ^{1}H NMR (400 MHz, Chloroform-d)

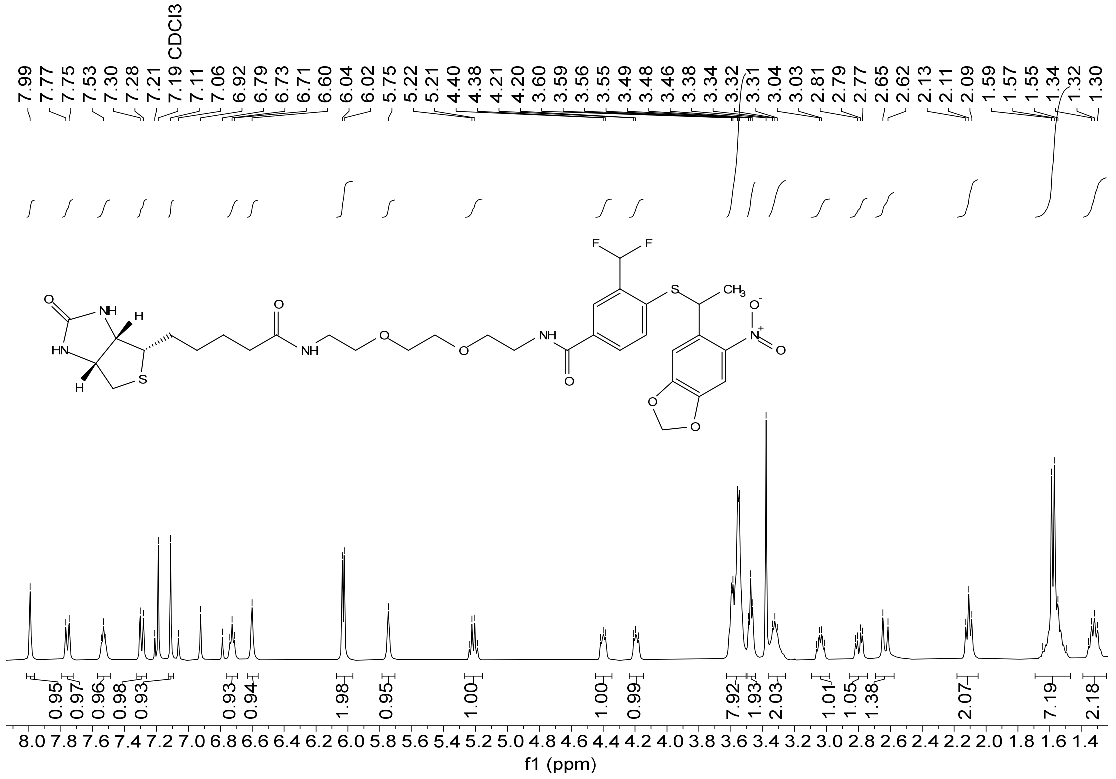

6dd: ^{13}C NMR (126 MHz, Chloroform-*d*)

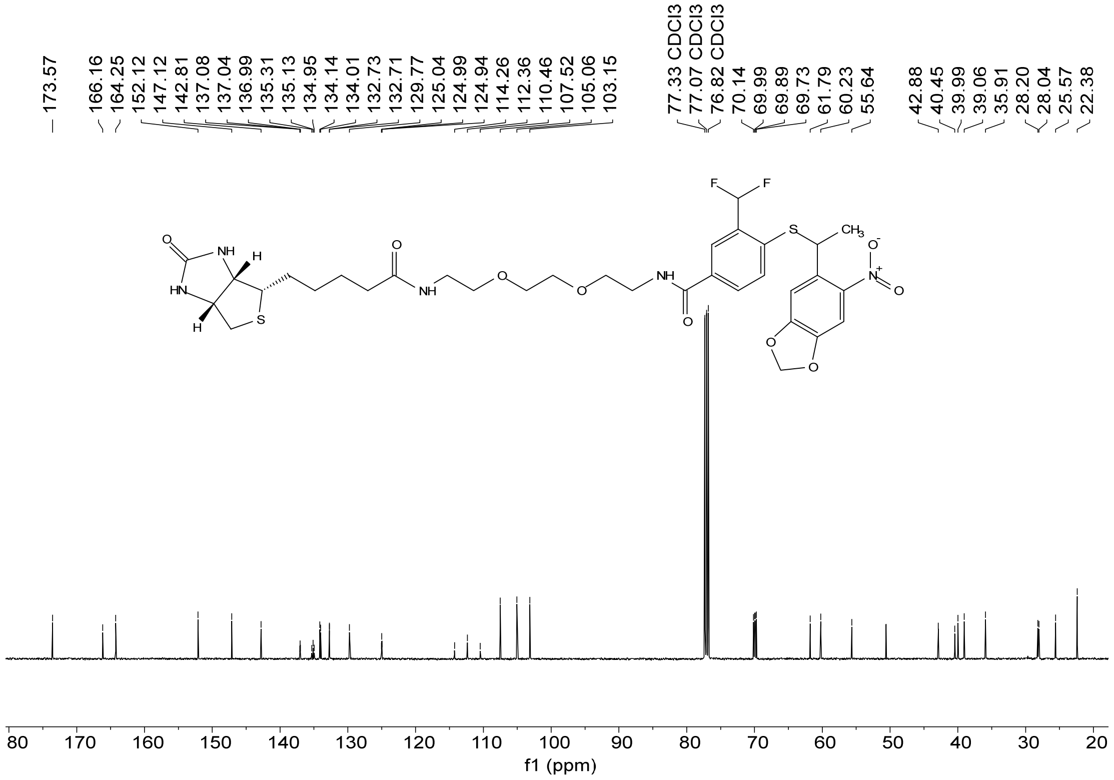

BSA MS2 SPECTRA

Potential **SF2** labeling sites:

Q440

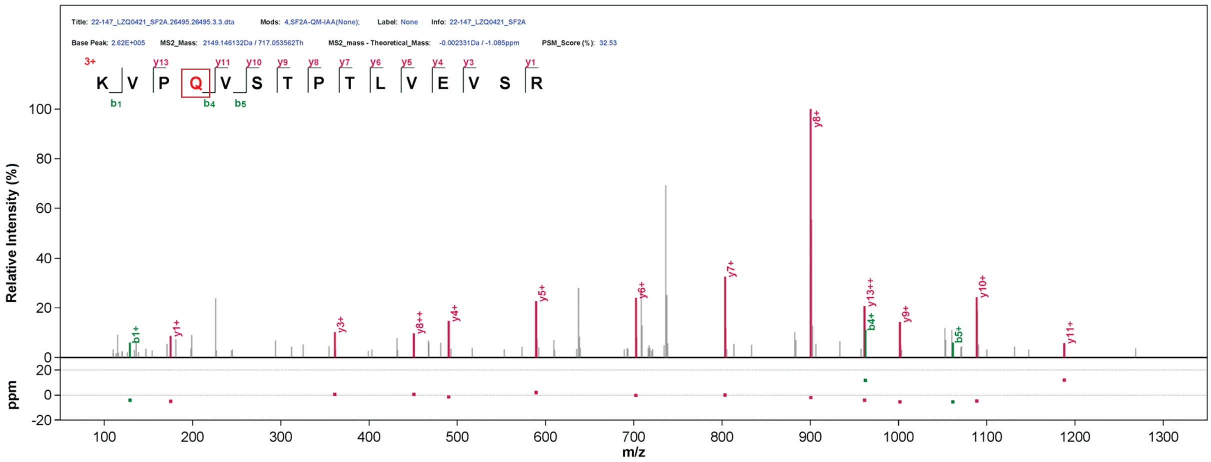

K437

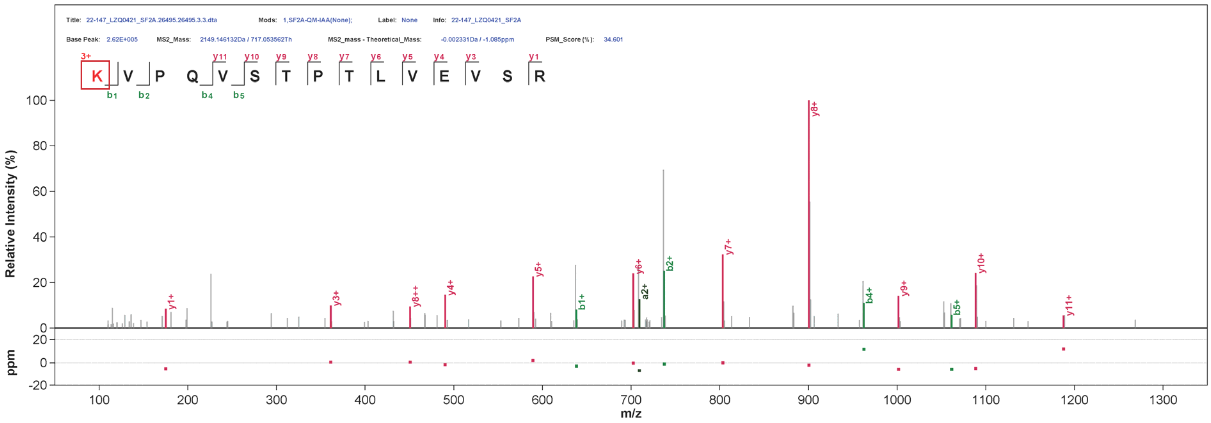

K156, W158

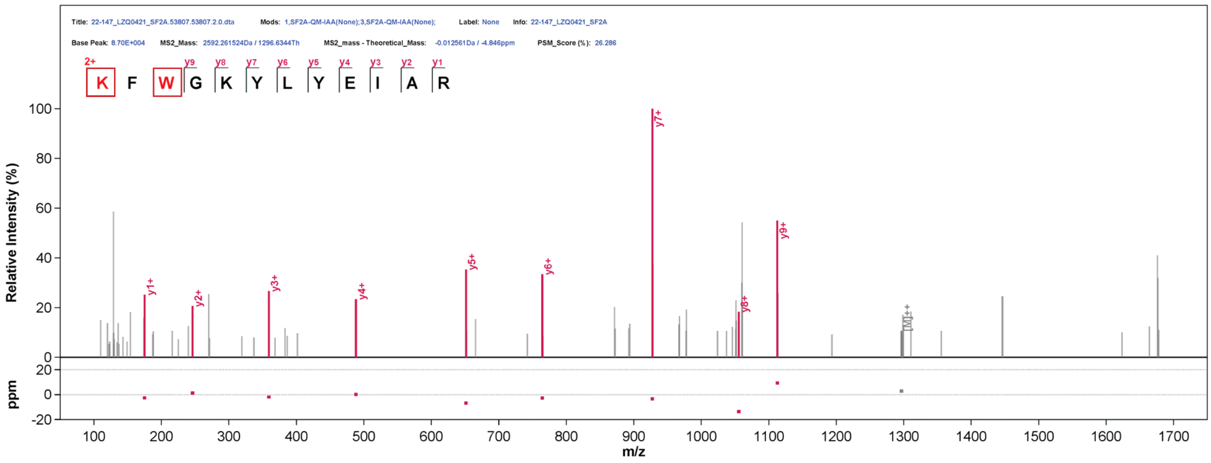

Y54, Q56

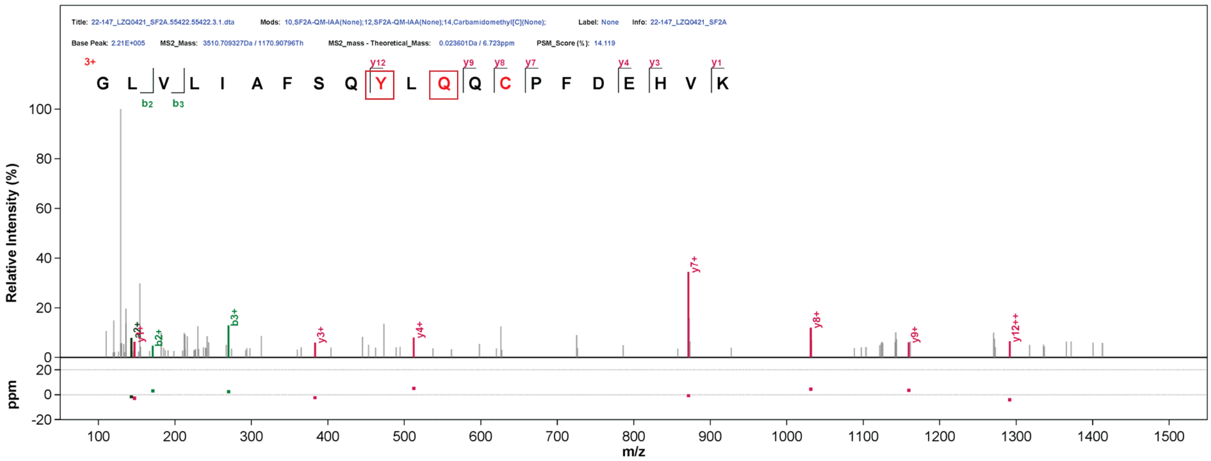

Q57 (or Q56)

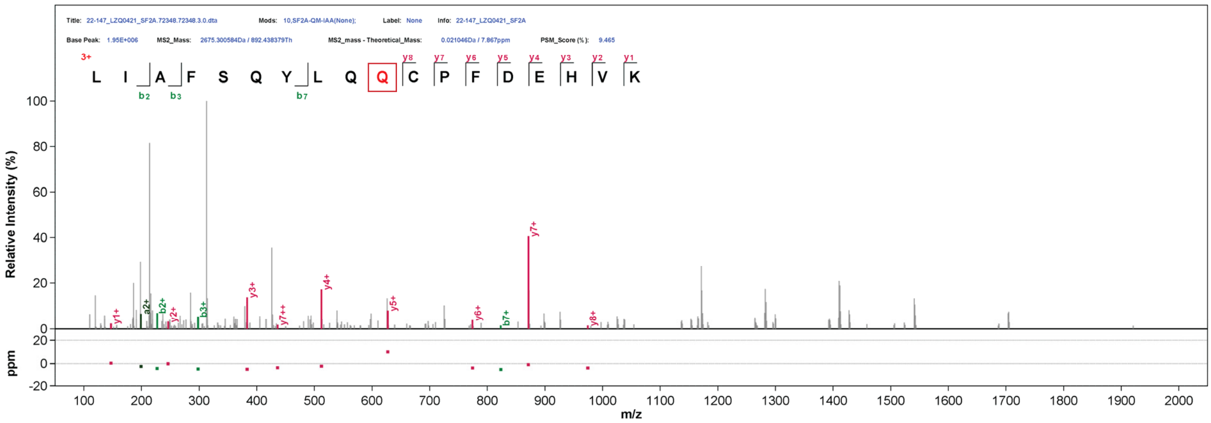

K160, Y161

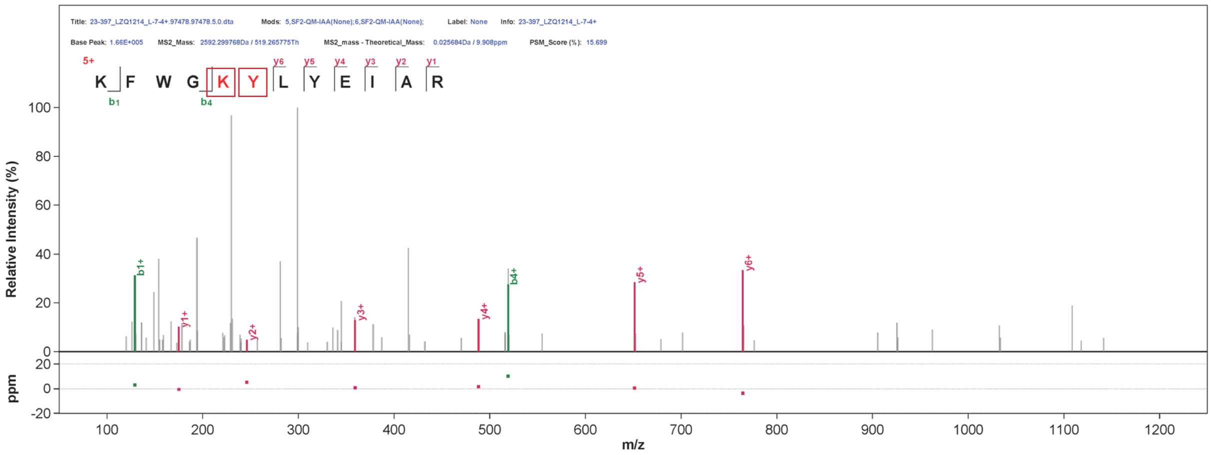

W158, K160

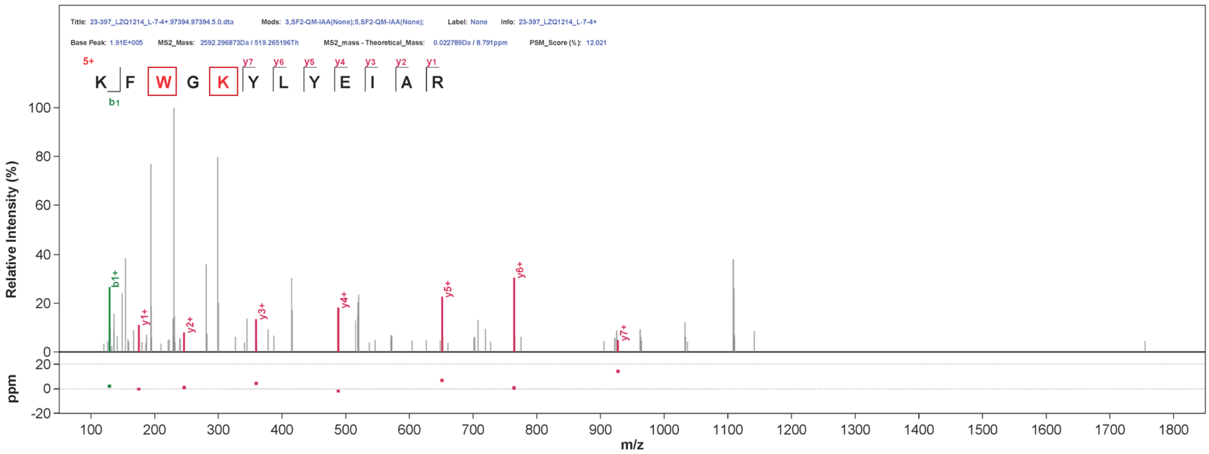

K140 (or K138)

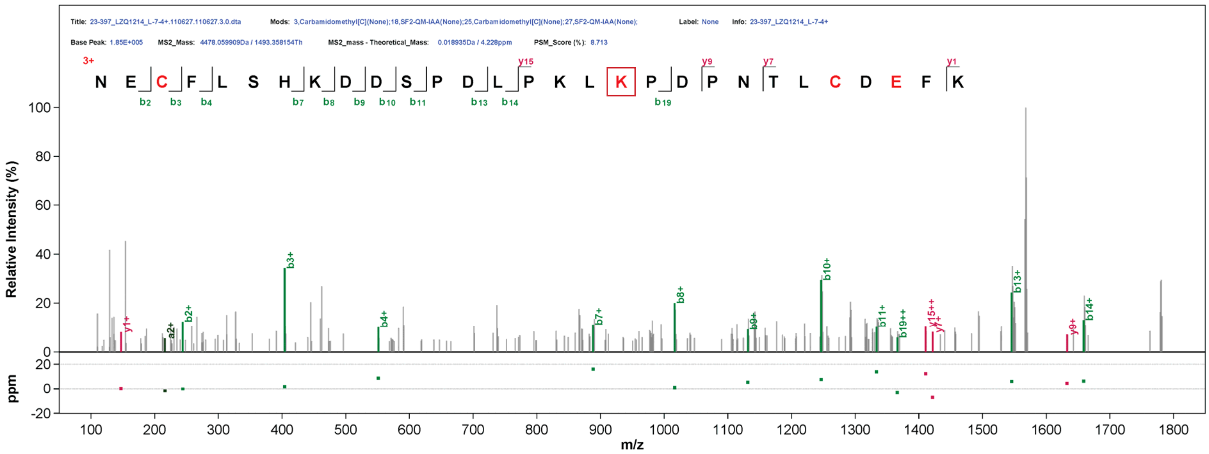

E308, K309

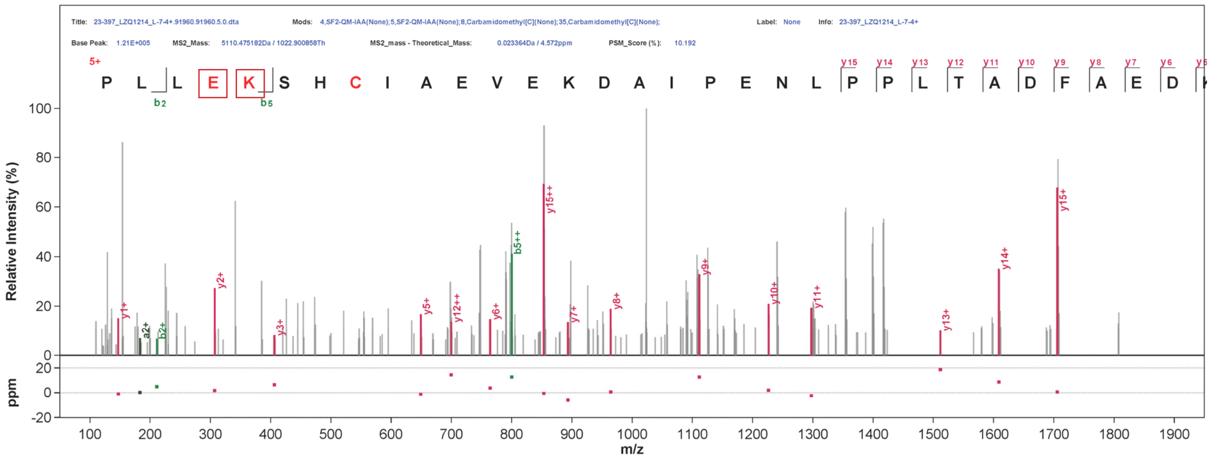

N182

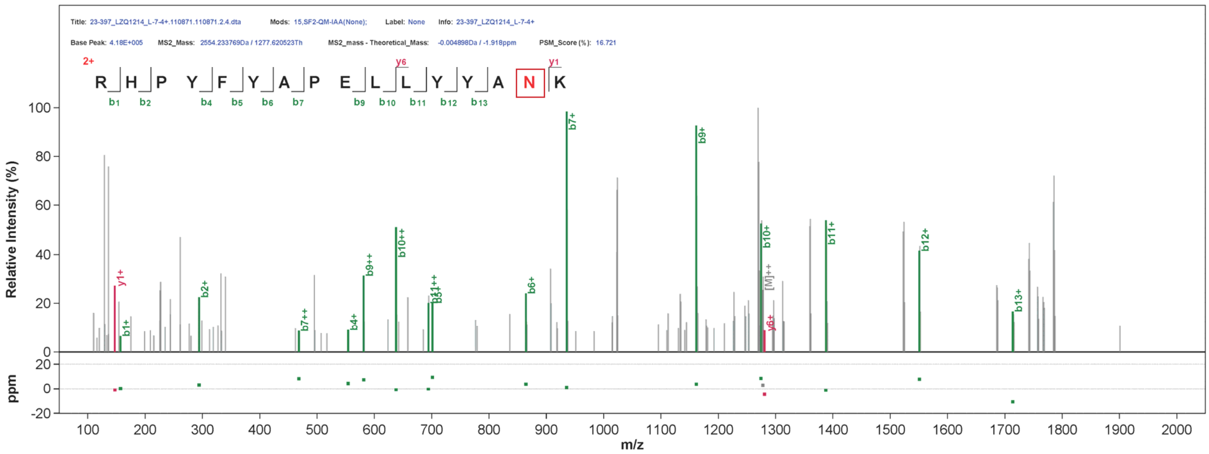

D80

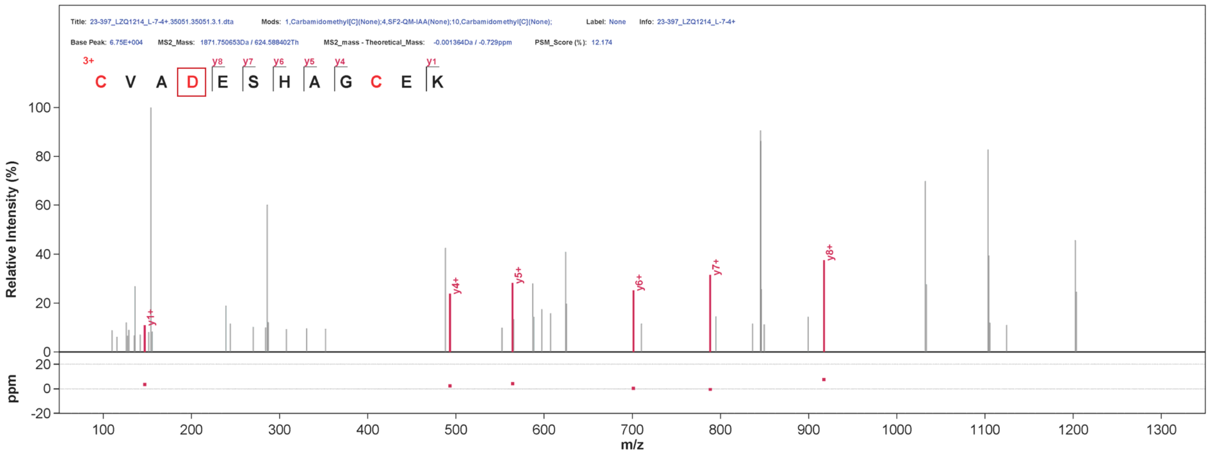

K140, D142

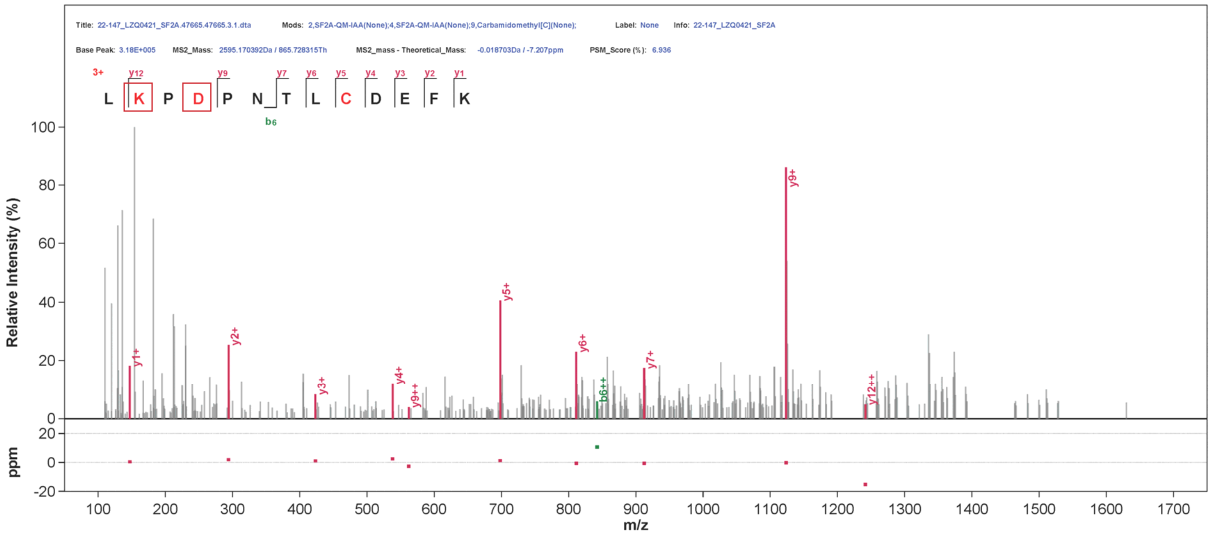

T501

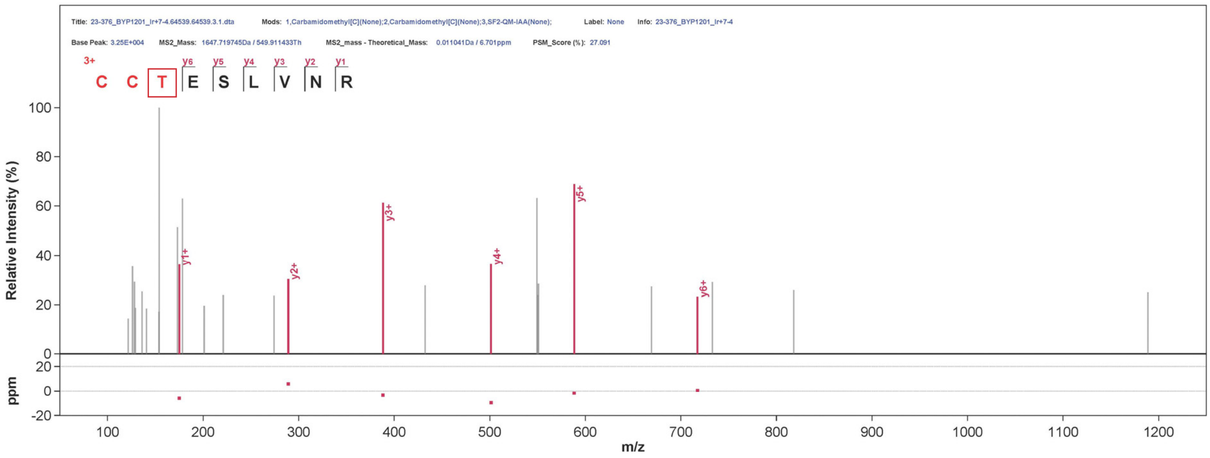

K65

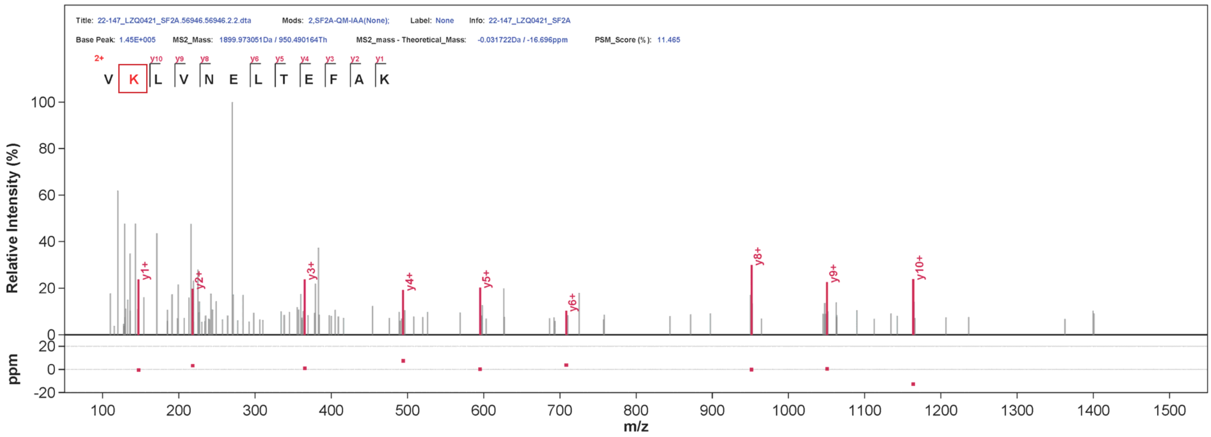

Potential side labeling (by arylazide) sites:

Y424

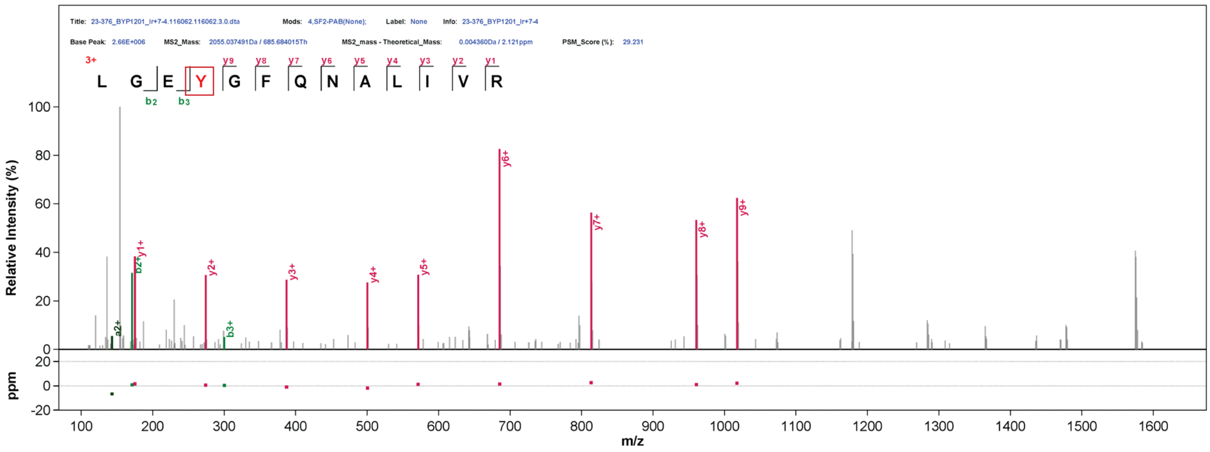

K498

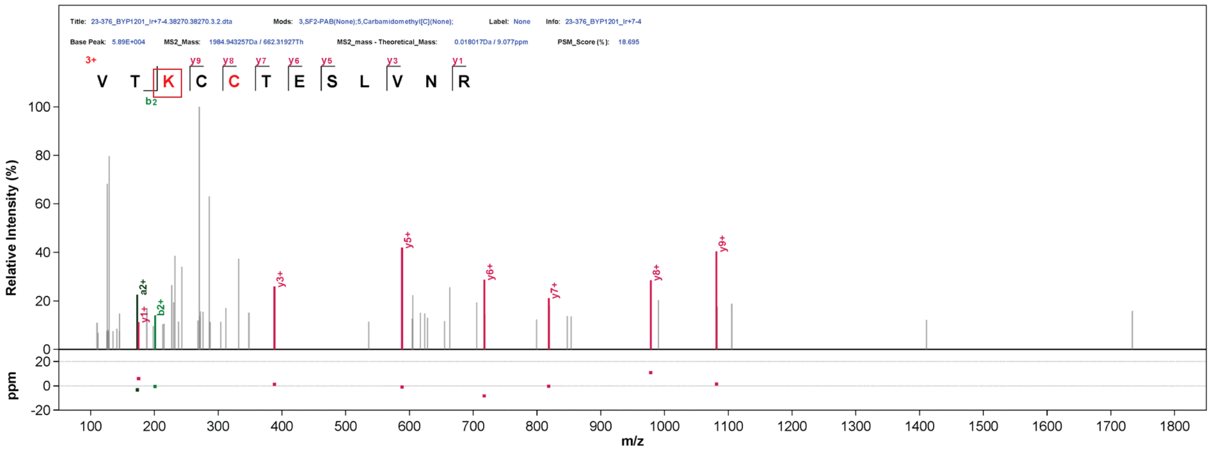

Y161

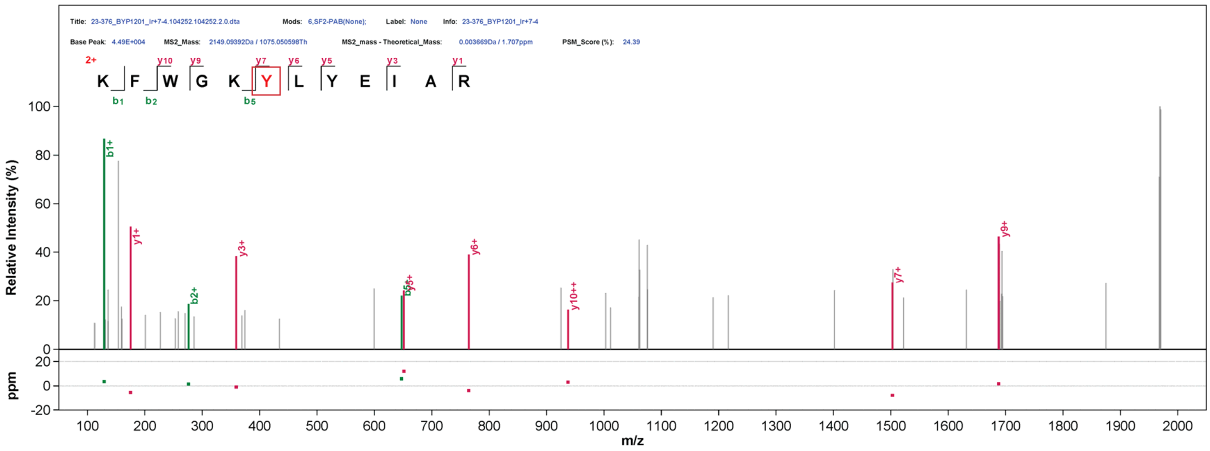

K156

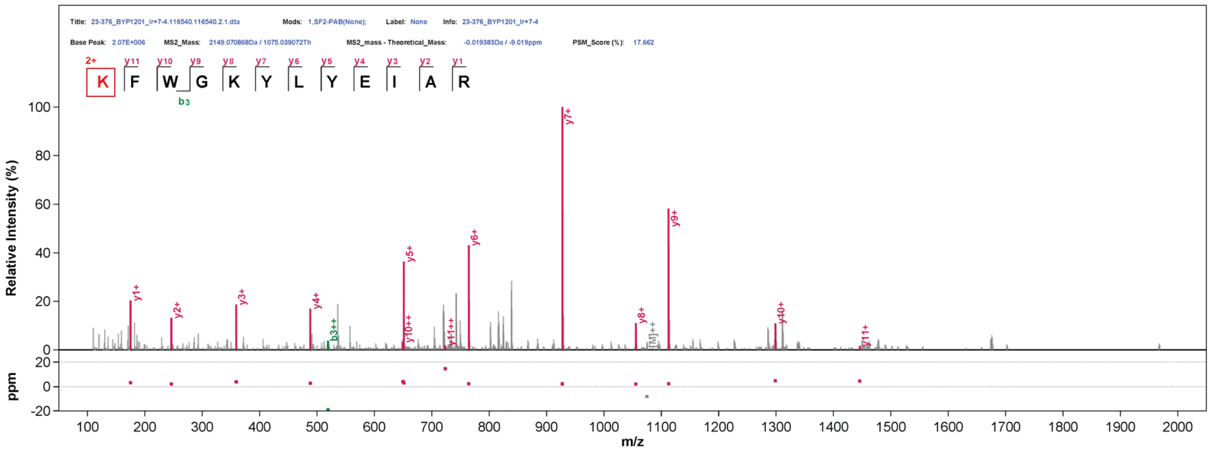

REFERENCES

1. Wang, H. et al. Selective mitochondrial protein labeling enabled by biocompatible photocatalytic reactions inside live cells. *JACS Au* **1**, 1066-1075 (2021).
2. Huang, Z. et al. Bioorthogonal photocatalytic decaging-enabled mitochondrial proteomics. *J. Am. Chem. Soc.* **143**, 18714-18720 (2021).
3. Liu, H., Lu, H.-H., Zhuang, J. & Thayumanavan, S. Three-component dynamic covalent chemistry: from Janus small molecules to functional polymers. *J. Am. Chem. Soc.* **143**, 20735-20746 (2021).
4. Del Vecchio, A. et al. Carbon isotope labeling of carbamates by late-stage [11C], [13C] and [14C]carbon dioxide incorporation. *Chem. Commun.* **56**, 11677-11680 (2020).
5. Zhu, H. et al. Imaging and profiling of proteins under oxidative conditions in cells and tissues by hydrogen-peroxide-responsive labeling. *J. Am. Chem. Soc.* **142**, 15711-15721 (2020).
6. Smaga, L.P., Pino, N.W., Ibarra, G.E., Krishnamurthy, V. & Chan, J. A Photoactivatable formaldehyde donor with fluorescence monitoring reveals threshold to arrest cell migration. *J. Am. Chem. Soc.* **142**, 680-684 (2020).
7. Hansen, F.M. et al. Mitochondrial phosphoproteomes are functionally specialized across tissues. *Life Sci. Alliance* **7** (2024).
