# Supplementary material for: Bioorthogonal photocatalytic proximity labeling in primary living samples
Source: Nat Commun. 2024 Mar 28;15:2712. doi: 10.1038/s41467-024-46985-3 (PMC10978841; doi:10.1038/s41467-024-46985-3)
Supplement: Supplementary file 3 — Description of Additional Supplementary Files [file 41467_2024_46985_MOESM3_ESM.pdf]

<u>Description of Additional Supplementary Files</u>

Supplementary Data 1. CAT-S proteomic data for cell lines

Supplementary Data 2. Additional analysis for mitochondrial protein discovery

Supplementary Data 3. CAT-S proteomic data for mouse tissues

Supplementary Data 4. CAT-S proteomic data for obese-diabetic kidney

Supplementary Data 5. CAT-S proteomic data for human primary T cells

Supplementary Software. tSNE visualization for CAT-S dat
