# Supplementary material for: Bioorthogonal photocatalytic proximity labeling in primary living samples
Source: Nat Commun. 2024 Mar 28;15:2712. doi: 10.1038/s41467-024-46985-3 (PMC10978841; doi:10.1038/s41467-024-46985-3)
Supplement: Supplementary file 10 — Reporting Summary [file 41467_2024_46985_MOESM10_ESM.pdf]

Corresponding author(s): Peng R. Chen, Xinyuan Fan

Last updated by author(s): Feb 29, 2024

Reporting Summary

Nature Portfolio wishes to improve the reproducibility of the work that we publish. This form provides structure for consistency and transparency in reporting. For further information on Nature Portfolio policies, see our Editorial Policies and the Editorial Policy Checklist.

Statistics

For all statistical analyses, confirm that the following items are present in the figure legend, table legend, main text, or Methods section.

n/a	Confirmed	
☐	☒	The exact sample size (*n*) for each experimental group/condition, given as a discrete number and unit of measurement
☐	☒	A statement on whether measurements were taken from distinct samples or whether the same sample was measured repeatedly
☐	☒	The statistical test(s) used AND whether they are one- or two-sided<br>*Only common tests should be described solely by name; describe more complex techniques in the Methods section.*
☒	☐	A description of all covariates tested
☐	☒	A description of any assumptions or corrections, such as tests of normality and adjustment for multiple comparisons
☐	☒	A full description of the statistical parameters including central tendency (e.g. means) or other basic estimates (e.g. regression coefficient) AND variation (e.g. standard deviation) or associated estimates of uncertainty (e.g. confidence intervals)
☐	☒	For null hypothesis testing, the test statistic (e.g. *F*, *t*, *r*) with confidence intervals, effect sizes, degrees of freedom and *P* value noted<br>*Give P values as exact values whenever suitable.*
☒	☐	For Bayesian analysis, information on the choice of priors and Markov chain Monte Carlo settings
☒	☐	For hierarchical and complex designs, identification of the appropriate level for tests and full reporting of outcomes
☐	☒	Estimates of effect sizes (e.g. Cohen's *d*, Pearson's *r*), indicating how they were calculated

Our web collection on statistics for biologists contains articles on many of the points above.

Software and code

Policy information about availability of computer code

Data collection	Image Lab Touch (v2.3.0, BioRad) was used to acquire all blot images. ZEN software (v2.1, ZEISS) was used to acquire fluorescent images. Flow cytometry data was acquired on LSRFortessa cell analyzer supplemented with BD FACSDiva (v7.0, BD biosciences) software. Plate reader data was acquired with Gen5 (v3.11.19, BioTek) software. The proteomic LC-MS/MS data was acquired with Thermo Xcalibur (v4.1.50). FT-HRMS data were acquired with ftmsControl (v2.2.0, Bruker). NMR spectra were acquired with IconNMR (v5.0.7, Bruker).
Data analysis	Image Lab (v6.0.0, BioRad) was used for processing and quantifying blot images. ZEN blue edition (v3.2, ZEISS) was used for processing fluorescent images. MS-based proteomic data were searched using MaxQuant (v1.6.10) or pFind (v1.3.5) softwares. Probe-modified peptide data was interpreted using pBuild (v3.0). A custom Python (v3.8) script including the packages scikit-learn (v1.1.3), matplotlib (v3.6.2), and NumPy(v1.23.4) was used for TSNE analysis, available on GitHub at https://github.com/hefei8alex/CAT-S_scripts. MestReNova (v9.0.1, Mestrelab) was used to analyze NMR spectra. GraphPad Prism (v8.0.2) was used to analyze numerical data and generate plots. Flow cytometry data was analyzed using FlowJo v10 (FlowJo, LLC).

For manuscripts utilizing custom algorithms or software that are central to the research but not yet described in published literature, software must be made available to editors and reviewers. We strongly encourage code deposition in a community repository (e.g. GitHub). See the Nature Portfolio guidelines for submitting code & software for further information.

Data

Policy information about availability of data

All manuscripts must include a data availability statement. This statement should provide the following information, where applicable:

- Accession codes, unique identifiers, or web links for publicly available datasets
- A description of any restrictions on data availability
- For clinical datasets or third party data, please ensure that the statement adheres to our policy

Data supporting the findings of this study are available in the Article and Supplementary Information. Source data are provided with this paper. The mass spectrometry proteomics data generated in this study have been deposited to the ProteomeXchange Consortium via the PRIDE partner repository with the dataset identifier PXD045791 [https://www.ebi.ac.uk/pride/archive/projects/PXD045791]. Protein annotation information were obtained from UniProt (https://www.uniprot.org) and MitoCarta (https://www.broadinstitute.org/mitocarta) databases. Biological function and pathway information were obtained from Gene Ontology database (https://geneontology.org). Protein-protein interaction information were obtained from STRING database (https://cn.string-db.org). Previously published mouse tissue proteomics datasets for cross analysis is available on ProteomeXchange via identifier PXD030062 [https://www.ebi.ac.uk/pride/archive/projects/PXD030062].

Research involving human participants, their data, or biological material

Policy information about studies with human participants or human data. See also policy information about sex, gender (identity/presentation), and sexual orientation and race, ethnicity and racism.

Reporting on sex and gender	Sex and gender were not considered for cell line and publicly available datasets. PBMCs samples were sourced from men only.
Reporting on race, ethnicity, or other socially relevant groupings	This information is not available for human samples used in this study.
Population characteristics	This information is not available for human samples used in this study.
Recruitment	N/A
Ethics oversight	Ethics committee of Shanghai Zhaxin hospital (No. LP202006).

Note that full information on the approval of the study protocol must also be provided in the manuscript.

Field-specific reporting

Please select the one below that is the best fit for your research. If you are not sure, read the appropriate sections before making your selection.

☒ Life sciences ☐ Behavioural & social sciences ☐ Ecological, evolutionary & environmental sciences

For a reference copy of the document with all sections, see nature.com/documents/nr-reporting-summary-flat.pdf

Life sciences study design

All studies must disclose on these points even when the disclosure is negative.

Sample size	The sample sizes were not predetermined by statistical method, and were empirically set to n = 2 or 3 biological independent replicates (Nature. 569, 509-513 (2019); Cell. 180, 373-386 (2020)). All CAT-S proteomic experiments were performed using 3 biologically independent samples. Animal study for validation of regulated proteins was performed using 3 pairs of diabetic and nondiabetic mice. All cell imaging results were representatives of at least 5 independent fields of view. Colocalization analysis were performed using at least 12 individual cells from 5-10 fields of view. Sample size for each experiment is indicated in the figure legend.
Data exclusions	There were no data exclusions.
Replication	All experiments were confirmed with multiple biological replicates as indicated in the Figure legends, and the representative results are shown.
Randomization	All samples and cells were randomly allocated into experimental groups.
Blinding	Investigator was blinded when pairing diabetic and nondiabetic mice. Investigator was blinded to group allocation during imaging validation of "mito orphans" and controls. For other experiments, the investigators were not blinded to sample identity, since the data was from objective quantitative methods so subjective bias was not relevant.

Reporting for specific materials, systems and methods

We require information from authors about some types of materials, experimental systems and methods used in many studies. Here, indicate whether each material, system or method listed is relevant to your study. If you are not sure if a list item applies to your research, read the appropriate section before selecting a response.

Materials & experimental systems

n/a	Involved in the study
☐	☒ Antibodies
☐	☒ Eukaryotic cell lines
☒	☐ Palaeontology and archaeology
☐	☒ Animals and other organisms
☒	☐ Clinical data
☒	☐ Dual use research of concern
☒	☐ Plants

Methods

n/a	Involved in the study
☒	☐ ChIP-seq
☐	☒ Flow cytometry
☒	☐ MRI-based neuroimaging

Antibodies

Antibodies used	All the antibody used in this study are commercially available: Rabbit Anti-ACSM2A mAb (Abcam, cat#ab181865, lot#gr151518-2, clone EPR13330), Rabbit Anti-AIF mAb (Abcam, cat#ab32516, lot#1000207-5, clone E20), Rabbit Anti-Aldh3a2 mAb (Abcam, cat#ab184171, lot#gr3181301-1, clone EPR15425(B)), Rabbit Anti-CPT1B pAb (Abcam, cat#ab134988, lot#gr3442894-1), Rabbit Anti-HSP60 mAb (Abcam, cat#ab45134, lot#gr3347668-1, clone EP1006Y), Goat Anti-Mouse-AlexaFluor555 (Invitrogen, cat#A-21422, lot#2214478), Goat Anti-Rabbit-AlexaFluor488 (Invitrogen, cat#A-11008, lot#2420731), Goat Anti-Rabbit-AlexaFluor546 (Invitrogen, cat#A-11010, lot#22423676), Mouse Anti-V5 mAb (Biodragon, cat#B1005, lot#kjb2123505), Horse HRP-linked anti-mouse IgG (Cell Signaling Technology, cat#7076S, lot#36), Goat HRP-linked anti-rabbit IgG (Cell Signaling Technology, cat#7074S, lot#31), Streptavidin-AlexaFluor488 (Invitrogen, cat#S11223, lot#2480092), Streptavidin-HRP (Cell Signaling Technology, cat#3999S, lot#9), Mouse Anti-Biotin mAb(SCBT, cat#sc-101339, lot#G1422, clone 33)
Validation	Validation of all antibodies can be found on the manufacturer's website. All the validation results can be founded on the websites by searching the catalog number. Website of abcam: https://www.abcam.com/, Website of Cell Signaling Technology: https://www.cellsignal.com/, Website of Santa Cruz Biotechnology: https://www.scbt.com/, Website of Invitrogen: https://www.invitrogen.com/, Website of Biodragon: https://www.biodragon.cn/.Rabbit Anti-ACSM2A mAb was validated by western blot, immunoprecipitation based on information on the website (https://www.abcam.cn/products/primary-antibodies/acsm2a-antibody-epr13330-ab181204.html). Rabbit Anti-AIF mAb was validated by western blot, immunoprecipitation and by manufacturer provided citations (64 citations) based on information on the website (https://www.abcam.cn/products/primary-antibodies/aif-antibody-e20-mitochondrial-marker-ab32516.html). Rabbit Rabbit Anti-Aldh3a2 mAb was validated by western blot, immunoprecipitation based on information on the website (https://www.abcam.cn/products/primary-antibodies/aldehyde-dehydrogenase-10-antibody-epr15425b-ab184171.html). Rabbit Anti-CPT1B pAb was validated by western blot, immunoprecipitation and by manufacturer provided citations (23 citations) based on information on the website (https://www.abcam.cn/products/primary-antibodies/cpt1b-antibody-ab134988.html). Rabbit Anti-HSP60 mAb was validated by western blot, immunoprecipitation and by manufacturer provided citations (15 citations) based on information on the website (https://www.abcam.cn/products/primary-antibodies/hsp60-antibody-ep1006y-loading-control-ab45134.html). Goat Anti-Mouse-AlexaFluor555 was validated by immunocytochemistry, flow cytometry and by manufacturer provided citations (1071 citations) based on information on the website (https://www.thermofisher.cn/cn/zh/antibody/product/Goat-anti-Mouse-IgG-H-L-Cross-Adsorbed-Secondary-Antibody-Polyclonal/A-21422). Goat Anti-Rabbit-AlexaFluor488 was validated by Immunocytochemistry, flow cytometry and by manufacturer provided citations (8879 citations) based on information on the website (https://www.thermofisher.cn/cn/zh/antibody/product/Goat-anti-Rabbit-IgG-H-L-Cross-Adsorbed-Secondary-Antibody-Polyclonal/A-11008). Goat Anti-Rabbit-AlexaFluor546 was validated by Immunocytochemistry, Flow Cytometry and by manufacturer provided citations (978 citations) based on information on the website (https://www.thermofisher.cn/cn/zh/antibody/product/Goat-anti-Rabbit-IgG-H-L-Cross-Adsorbed-Secondary-Antibody-Polyclonal/A-11010). Horse HRP-linked anti-mouse IgG was validated by western blot and by manufacturer provided citations (7076 citations) based on information on the website (https://www.cellsignal.cn/products/secondary-antibodies/anti-mouse-igg-hrp-linked-antibody/7076). Goat HRP-linked anti-rabbit IgG was validated by western blot and by manufacturer provided citations (15295 citations) based on information on the website (https://www.cellsignal.cn/products/secondary-antibodies/anti-rabbit-igg-hrp-linked-antibody/7074). Streptavidin-AlexaFluor488 was validated by Immunocytochemistry, Flow Cytometry and by manufacturer provided citations (88 citations) based on information on the website (https://www.thermofisher.cn/order/catalog/product/S11223). Streptavidin-HRP was validated by western blot based on information on the website (https://www.cellsignal.cn/products/wb-ip-reagents/streptavidin-hrp/3999). Mouse Anti-Biotin mAb was validated by western blot, immunoprecipitation and by manufacturer provided citations (33 citations) based on information on the website (https://www.scbt.com/p/biotin-antibody-33).

Eukaryotic cell lines

Policy information about cell lines and Sex and Gender in Research

Cell line source(s)	HeLa, HEK293T and K562 cells were purchased from Cell Resource Center, Peking Union Medical College, China.
Authentication	The cell lines were frequently checked by their morphological features and the cell lines were not authenticated by the short tandem repeat (STR) profiling.

Mycoplasma contamination | All cell lines were tested to be mycoplasma-negative by the standard PCR method.

Commonly misidentified lines (See ICLAC register) | No commonly misidentified cell lines are used in this study.

Animals and other research organisms

Policy information about studies involving animals; ARRIVE guidelines recommended for reporting animal research, and Sex and Gender in Research

Laboratory animals	C57BL/6J (B6) mice (female, 6-8-week, Cat# 219) were purchased from Beijing Vital River Laboratory Animal Technology (Beijing), China. db/db obese-diabetic mice (male, 7-8-week) and m/m nondiabetic mice (male, 7-8-week) were purchased from Cavens Laboratory Animal Technology (Changzhou), China. All the mice were housed at 18-24 degree Celsius ?with 40-70% humidity and a 14-h light/10-h dark cycle.
Wild animals	No wild animals were used in this study.
Reporting on sex	Sex was not considered in the study design.
Field-collected samples	The study did not involve samples collected from the field.
Ethics oversight	All animal studies were approved by the Institutional Animal Care and Use Committee of Peking University (CCME-ChenP-3).

Note that full information on the approval of the study protocol must also be provided in the manuscript.

Plants

Seed stocks	*Report on the source of all seed stocks or other plant material used. If applicable, state the seed stock centre and catalogue number. If plant specimens were collected from the field, describe the collection location, date and sampling procedures.*
Novel plant genotypes	*Describe the methods by which all novel plant genotypes were produced. This includes those generated by transgenic approaches, gene editing, chemical/radiation-based mutagenesis and hybridization. For transgenic lines, describe the transformation method, the number of independent lines analyzed and the generation upon which experiments were performed. For gene-edited lines, describe the editor used, the endogenous sequence targeted for editing, the targeting guide RNA sequence (if applicable) and how the editor was applied.*
Authentication	*Describe any authentication procedures for each seed stock used or novel genotype generated. Describe any experiments used to assess the effect of a mutation and, where applicable, how potential secondary effects (e.g. second site T-DNA insertions, mosiacism, off-target gene editing) were examined.*

Flow Cytometry

Plots

Confirm that:

☒ The axis labels state the marker and fluorochrome used (e.g. CD4-FITC).

☒ The axis scales are clearly visible. Include numbers along axes only for bottom left plot of group (a 'group' is an analysis of identical markers).

☒ All plots are contour plots with outliers or pseudocolor plots.

☒ A numerical value for number of cells or percentage (with statistics) is provided.

Methodology

Sample preparation	Sample preparation is described in detail in the method section of "Mitochondrial membrane potential (MMP) assay" in Supplementary Information.
Instrument	LSRFortessa cell analyzer (BD biosciences)
Software	BD FACSDiva for data collection; Flowjo (v10) for data analysis.
Cell population abundance	The fluorescent marker for analysis was intracellular JC-1 probe. About 10,000 cells were recorded for each test group.
Gating strategy	Single cells were gated on their FSC/SSC, FSC/FSC-H distributions. "MMP retained" and "MMP loss" populations were gated based on JC-1 aggregate (PE channel) and JC-1 monomer (FITC channel) fluorescence.

☒ Tick this box to confirm that a figure exemplifying the gating strategy is provided in the Supplementary Information.
